# Physical, rheological and microscopic properties of AAT nanomaterial/crumb rubber powder composite-modified asphalt and SBS-modified asphalt

**DOI:** 10.1371/journal.pone.0284813

**Published:** 2024-01-11

**Authors:** Lu Sun, Wenqing Zhong, Ziwei Xiao, Hui Qi

**Affiliations:** 1 Department of Civil Engineering Technology, Rochester Institute of Technology, Environmental Management and Safety, Rochester, NY, United States of America; 2 College of Architectural Science and Engineering, Yangzhou University, Yangzhou, 225009, Jiangsu, China; 3 College of Traffic and Transportation, Chongqing Jiaotong University, Chongqing, 400074, China; 4 School of Qilu Transportation, Shandong University, Jinan, 250002, China; 5 Shandong High-Speed Group, Co. Ltd., Jinan, 250101, China; Shandong University of Technology, CHINA

## Abstract

This research was based on a nano-AAT (American Advanced Technology)-modified asphalt to which CRP (crumb rubber powder), a rubber waste, was introduced to explore the influence of CRP on AAT performance. The changes in the performance of AAT-modified asphalt after the addition of CRP were analyzed. The rubber powder with the raw material of waste tire was added to the asphalt instead of SBS modifier. While achieving waste recycling, the asphalt material has good performance. Physical analysis methods, rheological performance tests, rolling thin-film oven tests and Fourier transform infrared spectroscopy tests were used to investigate the performance of the composite-modified asphalt. The rheological properties of the composite-modified asphalt were analyzed by means of DSR, BBR and MSCR tests, and the microscopic mechanism of the modified asphalt was investigated by means of FTIR tests. The optimal nano-AAT-composite-modified formulation A3C3 (AAT-3.5%SBS-3%CRP) was selected by evaluating the overall performance. Additionally, the performances of the AAT/CRP-composite-modified asphalt and SBS-modified asphalt were compared using physical indicators, the rutting factor, creep flexibility and the stiffness modulus. The results show that the A3C3-modified asphalt had better stiffness, high-temperature (HT) performance and aging resistance than the SBS-modified asphalt, but it was less effective at low temperatures (LTs). According to FTIR, the absorption curves of A3C3 and SBS are essentially equal, with A3C3 only having a variation at 1104 cm^−1^.

## 1. Introduction

Modified bitumen has undergone years of development. New studies keep coming up with new kinds of asphalt modifiers, with improved methods for modifying asphalt. This makes different kinds of modified asphalt systems critical in building roads. With the constant improvement in asphalt modification and the need for long-lasting road, polymer modification and nanomaterial-modification are increasingly being explored in research and development, and have been proven to improve asphalt’s durability. SBS indicates a styrene–butadiene–styrene triblock copolymer [1]. It can form a multidimensional network structure inside asphalt and effectively fix asphalt’s shortcomings via rutting at HT and cracking at LT, so it is widely used for asphalt modification [2]. However, after long-term practical application, SBS-modified asphalt is still subject to polymer cracking and has an insufficient capacity for long-term engineering service, with potential problems after long-term service.

Nanomaterials can improve polymer-modified asphalt, particularly in terms of extending its service life. Nanomaterials, due to their size effect, can produce functions that are not available with conventional materials, and their excellent age resistance and ability to purify exhaust gases provides the foundation for their use in asphalt. Ven De Ven et al. used nano-montmorillonite clay was added to modified asphalt and tested as the binding material in the mix, and it was found to be effective in improving the aging resistance, HT rutting resistance and mechanical properties of asphalt materials [3]. Sun and his associates [4,5] conducted a pioneer work in identifying the advantages of nanomaterials in asphalt modification and proved nanomaterial modified asphalt pavement performance in large-scale road test in several freeways. They also conducted a comprehensive and extensive study on nanomaterial modification and nano-composite material modification, on mechanical properties of asphalt mixtures [6–18]. Nano-montmorillonite with a special layered structure can be used to block contact be-tween the asphalt and the outside to improve the ageing resistance of asphalt materials [19]. Xu et al. [20] discovered that ZnO nanoparticles could effectively enhance the resistance of asphalt to UV aging and improve its HT rheological properties. The incorporation of nanomaterials improved the long-term serviceability of the asphalt material, but it was deficient in terms of viscoelastic properties and cracking resistance. Blending nanomaterials with polymers can lead to high-performing modified asphalt materials. Lu et al. [21] analyzed the stability and rheological properties of a composite-modified asphalt comprising nano-montmorillonite and SBS with multiple admixtures and found that the addition of nano-montmorillonite enhanced the high-temperature rutting resistance and storage stability of the SBS-modified asphalt but degraded its low-temperature performance. Wang et al. [22] analyzed the mechanism of different carbon nanotubes in SBS-modified asphalt using microscopic analysis equipment and found that carbon nanotubes have a beneficial effect on its HT performance, anti-aging properties and service life. Hakeem et al. [23] used a combination of microscopic analytical instruments to assess the effects of nano-sesame straw ash (NSSA) and rice straw ash (RSA) on high-strength concrete (HSC). The ash was discovered to be useful in eliminating carbon and unburned organic debris, resulting in increased mechanical strength for HSC. After the polymer and nanomaterials reach a state of co-integration with the asphalt material, they tightly bind to it, conferring damage resistance in several ways. However, high-performance modified asphalt is still subject to the influence of many factors, such as cost and process, when it is put to use as pavement. To reduce the cost of such projects, waste rubber powder is often used in place of some of the polymer modifiers (SBS, etc.) due to its ability to improve the overall performance of the asphalt material at a lower cost [24]. Most current waste rubber powder comes from waste tires, which by their very nature are rubber, difficult to degrade and decompose and are prone to producing harmful gases. The recycling of waste rubber powder from used tires into modifiers that can be used in road materials can significantly reduce its environmental impact [25]. Liu et al. [26], in a study on macroscopic physical change in composite modification using various doses of SBS and rubber powder, found that the rubber powder effectively enhanced the SBS-modified asphalt’s stiffness, resistance to HT deformation and elastic recovery. Liang et al. [27] analyzed the rheological properties and storage stability of the SBS–rubber powder-composite-modified asphalt using DSR and fluorescence electron microscopy, and the results show that the combination of the two materials conferred good viscoelastic properties and storage stability on the asphalt. Qian et al. [28] conducted a multifaceted and multilevel mechanistic analysis of asphalt modified with rubber powder and SBS. This showed the mechanism of action of the dispersed rubber and SBS copolymer in asphalt and showed that the SBS copolymer and rubber provided good storage stability for asphalt binders. Qian et al. [29] mixed SiO_2_ nanoparticles and rubber powder into an asphalt matrix for a composite modification study and found that the nano-SiO_2_ and rubber powder formed a good barrier structure inside the asphalt, which effectively enhanced the anti-UV aging performance of the asphalt material.

Several studies have been carried out using different polymers and nanomaterials mixed with asphalt. Before the modified asphalt material is ready to be put into use, it is found that construction workability and project cost are still very critical issues. This study is based on the development of AAT nanomaterial modified asphalt, which is a nanocomposite-modified material formed by a combination of (5%) SBS, (1%) polymer A and (0.5%) nanoparticle B. It has significant age resistance and HT properties, as well as good cracking resistance. A variety of polymers and nanomaterials were combined to improve the overall performance of the asphalt material. Good asphalt materials need to be tested in engineering applications. To improve AAT’s suitability for engineering applications, it is necessary to reduce its cost to a certain extent without compromising its overall performance by replacing part of the SBS with inexpensive crumb rubber powder [30]. The use of CRP complements the performance of the modified asphalt while allowing the waste to be recycled, reducing its environmental impact. Therefore, the performance of AAT nanomaterial modified asphalt based on SBS and CRP was compared under the modulation of multiple doping groups to select the optimal AAT/CRP composite modification solution.

In this study, the stiffness, softness, ductility and viscosity of AAT/CRP composite-modified asphalt was evaluated using four physical property indicators. The high- and low-temperature performance of AAT/CRP-composite-modified asphalt was evaluated using dynamic shear rheometry (DSR) and bending beam rheometry (BBR). To evaluate the HT creep performance of AAT/CRP-composite-modified asphalt, mass storage was determined using RTFOT and multiple-stress creep performance (MSCR). Finally, the functional group of the AAT/CRP-composite-modified asphalt was studied using Fourier transform infrared spectroscopy (FTIR). The performance of the AAT nanomaterial modified asphalt was also evaluated by comparing SBS with CRP.

## 2. Raw materials

### 2.1. Overview

The base asphalt was a 70 AH base asphalt supplied by China Shandong Hi–Speed Infrastructure Construction Limited (Shandong, China), and its physical properties are shown in Table 1 below. The CRP used was 60-mesh CRP (The relevant indicators are shown in Table 2 below) produced by Xinlei Mining Co. Ltd. in Shijiazhuang, Hebei, China. To avoid errors in the comparison of asphalt performance, SBS modifiers (The relevant indicators are shown in Table 3 below) provided by the China Shandong Expressway Group were used. The physical properties of the SBS-modified asphalt are shown in Table 4 below. The abbreviated names of the modified asphalt materials are indicated in Table 5 for ease of reference.

**Table 1:** Physical properties of 70 AH base asphalt.

Indicators	Units	Results	Standard Range	Specification
Penetration @ 25°C	dmm	69.7	60~80	ASTM D5
Softening point @ 5°C	°C	49.5	>46	ASTM D36
Viscosity @ 135°C	Pa.s	0.427	<3.0	ASTM D4402
Ductility @ 10°C and 50 mm/min	mm	276	>200	ASTM D113

**Table 2:** Material properties of CRP.

Indicators	Units	Results	Range
Ash content	%	7.7	≤8
Tenor content	%	0.03	≤0.08
Fiber content	%	0	≤0.1
Calcium carbonate content	%	4.3	≤5
Crumb rubber powder density	kg/m3	1.16	≈1.15
2.00mm sieve pass rate	%	100	≥100
Carbon black content	%	29	≥26
volume density	kg/m3	369	260–460

**Table 3:** Material properties of SBS.

Indicators	Units	Results	Range
Molecular structure	-	line style	-
Block ratio	-	30/70	-
Volatile content	%	0.13	≤0.2
Melt flow index	-	0.11	0.01–0.50
Shore’ s hardness	A	71	≥68

**Table 4:** Physical properties of SBS-modified asphalt.

Indicators	Units	Results	Standard Range	Specification
Penetration @ 25°C	dmm	50.8	40~60	ASTM D5
Softening point @ 5°C	°C	67.7	>60	ASTM D36
Viscosity @ 135°C	Pa.s	1.557	<3.0	ASTM D4402
Ductility @ 5°C and 50 mm/min	mm	255	>200	ASTM D113

**Table 5:** Abbreviations for modified asphalt binder.

Materials	AAT (%)	SBS (%)	CRP (%)	Abbreviations
1	0	4.5	0	S
2	1.5	2.5	0	A1
3	1.5	3.0	0	A2
4	1.5	3.5	0	A3
5	1.5	2.5	3	A1C3
6	1.5	3.0	3	A2C3
7	1.5	3.5	3	A3C3
8	1.5	3.5	2	A3C2
9	1.5	3.5	1	A3C1

### 2.2. Preparation process

The preparation process for the nano-AAT-composite-modified asphalt consisted of the following three steps [31]. The specific steps are shown in Table 6.

**Table 6:** Preparation procedure.

Step1	We weighed the corresponding masses of A polymer particles, B nanoparticles, SBS particles and 60-mesh CRP, then mixed them with the base asphalt by hand and placed them in an oven at 180°C for 30 min to dissolve them.
Step2	The samples underwent high-speed shearing at a constant temperature of 180°C for 45 min at a shear rate of 5000 r·min^−1^.
Step3	After mixing, the samples were placed in an oven at 180°C for 30 min for developmental defoaming.

## 3. Tests

### 3.1. Physical property

The physical properties of nano-AAT-composite-modified asphalt and SBS-modified asphalt were compared in accordance with the testing requirements of the ASTM American Standard, using 25°C penetration tests, 5°C ductility tests and 5°C softening point tests for their relevant basic road properties [32–34].

### 3.2. Viscosity

A good-flowing asphalt material provides better preconditions for subsequent diversification studies. ASTM standards were used to run the Brookfield viscosity test on the modified asphalt to determine its viscosity [35]. The Brookfield viscosity test was carried out at 135°C. The viscosity at this temperature was close to that of the actual project and was used to evaluate the HT viscosity of the modified asphalt and the ease of construction.

### 3.3. Dynamic Shear Rheometer (DSR)

A temperature scan test was carried out to determine the HT rheological properties of asphalt using a dynamic shear rheometer, and the rutting factor (G*/Sinδ) was used to assess the level of rutting resistance of the modified asphalt binder. The Superpave specifications state that good HT performance can be defined as a rutting factor (G*/Sinδ) > 1.0 kPa. To investigate the conventional viscoelastic mechanical behaviors of modified asphalt, the test temperature was controlled from 52°C to 82°C, and specimens were prepared using 25 mm parallel plate samples [36]. The frequency was set to 10 rad / s (1.59 Hz), and the strain control was selected, and the strain level was set to 10%.

### 3.4. Bending Beam Rheometer (BBR)

Asphalt materials in LT environments usually exhibit certain brittle qualities and insufficient viscoelastic properties. To evaluate the cracking resistance of modified asphalt in a sub-zero-temperature environment, BBR tests were carried out under the SHRP programmer [37]. The LT properties of the modified bitumen bond were analyzed by determining the low-temperature modulus of stiffness S and creep rate m. The test temperatures of −12°C and −18°C were used for this study, and the data generated at 60 s were selected to meet the Superpave specification requirements for a modulus of stiffness S < 300 Mpa and m > 0.3 at 60 s.

### 3.5. Rolling Thin-Film Oven Test (RTFOT)

As the storage, mixing, transportation and paving of asphalt is often affected by various environmental factors, it is prone to a series of physical and chemical changes, which in turn lead to changes in its properties. The evaluation of short-term thermos-oxidative aging performance changes produced in real engineering was carried out using the RTFOT. The corresponding procedure was carried out according to ASTM D2872-19; the test temperature was maintained at around 163°C, and the asphalt was heated for 75 to 85 min [38].

### 3.6. Multiple-Stress Creep Recovery (MSCR)

The MSCR test is based on a dynamic shear rheometer, which can be used to analyze the HT viscoelastic properties of asphalt in relation to its delayed elastic recovery under applied stress. According to AASHTO T 350–2019, two stress levels, 0.1 kPa and 3.2 kPa, were selected for continuous testing in DSR stress control mode to simulate both light and heavy traffic loading conditions at a test temperature of 64°C [39]. The creep and recovery curves of asphalt under different stresses were collected throughout the tests, and from this, the strain recovery rate R and the irrecoverable creep flexibility J_nr_ were calculated.

### 3.7. Fourier Transform Infrared Spectroscopy (FT-IR)

Given the mechanism of asphalt modification and the effect of aging on its properties, changes in the chemical composition of its materials can be studied at the microscopic level. FTIR tests allow for the analysis of subtle chemical bonding and functional group changes, effectively facilitating the analysis of the microscopic mechanism of the material. The quantitative analysis of asphalt properties is achieved by comparing the chemical structure of various asphalt materials in infrared spectra and analyzing the changes in the absorption of the characteristic peaks of each spectrum [40]. After the thermal oxygen aging of the modified asphalt material, the sulfoxide characteristic peak at 1030cm^-1^ will change to a certain extent, to quantitatively determine whether new chemical functional groups appear. The sulfoxide index (SI) of modified asphalt before and after aging were calculated to quantitatively analyze the corresponding characteristic peak changes in the infrared spectrum. The specific calculation method is shown in the Formula (1).


$$\mathrm{SI}=\frac{A_{1030}}{A_{1461}}$$ (1)

In the formula:

A_1030_——The characteristic absorption peak area of sulfoxide group at 1030cm^-1^;

A_1461_——The characteristic absorption peak area of methylene group at 1461cm^-1^.

## 4. Results and analysis

### 4.1. Physical property

The findings in Table 7 show that adjusting the SBS concentration in the AAT-modified asphalt resulted in a drop in all physical attributes. When the quantity of SBS was reduced to 2.5%, the softening point in A1, which represents HT performance, declined by over 20.1%, and the ductile tensile value, which represents LT performance, plummeted by nearly 40%. When the amount of SBS was reduced to 3.5%, both A3 and SBS-modified asphalt had better penetration hardness; however, in terms of the softening point, A3 still decreased by nearly 15.4% compared to SBS, and in terms of LT ductility, A3 had a difference of nearly 25.1% compared to SBS. As a result of the SBS content adjustment, the hardness and softness of the AAT-modified asphalt differed significantly from those of the SBS-modified asphalt. The AAT-modified asphalt was complemented with a low-cost CRP to provide high overall performance at a cheaper cost.

**Table 7:** Physical properties of SBS-modified asphalt and AAT-modified asphalt.

Materials	25°C Penetration (dmm)	Softening Point (°C)	5°C Ductility (mm)
S	50.8	67.7	255
AAT	40.3	71.4	228
A1	53.2	54.1	153
A2	51.5	56.2	185
A3	50.1	57.3	191
A1C3	49.8	57.0	197
A2C3	45.7	60.3	207
A3C3	42.5	69.2	222
A3C2	45.6	67.1	206
A3C1	48.9	64.9	196

Note: To facilitate subsequent presentation, a brief description of each recipe is given.

**Fig 1:**
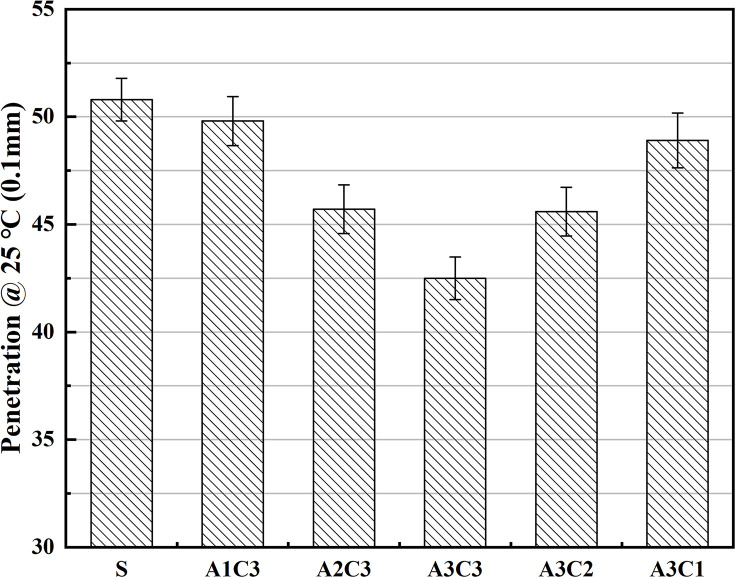
Differences in hardness.

**Fig 2:**
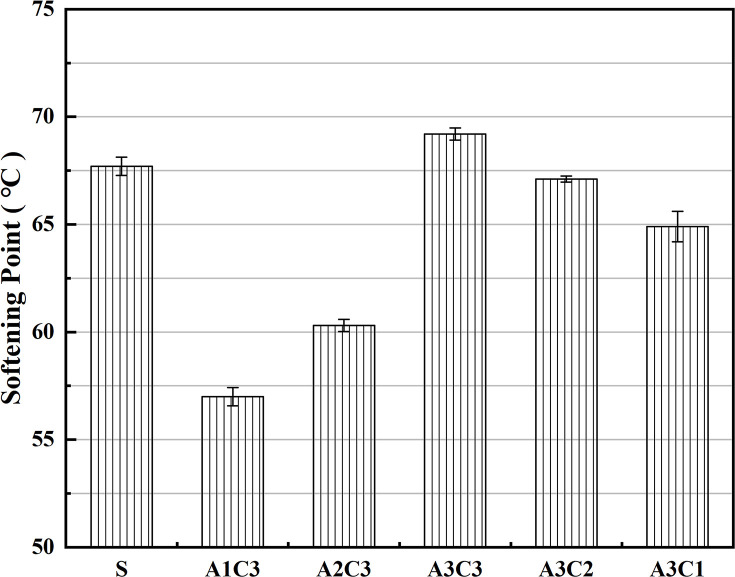
Differences in HT performance.

**Fig 3:**
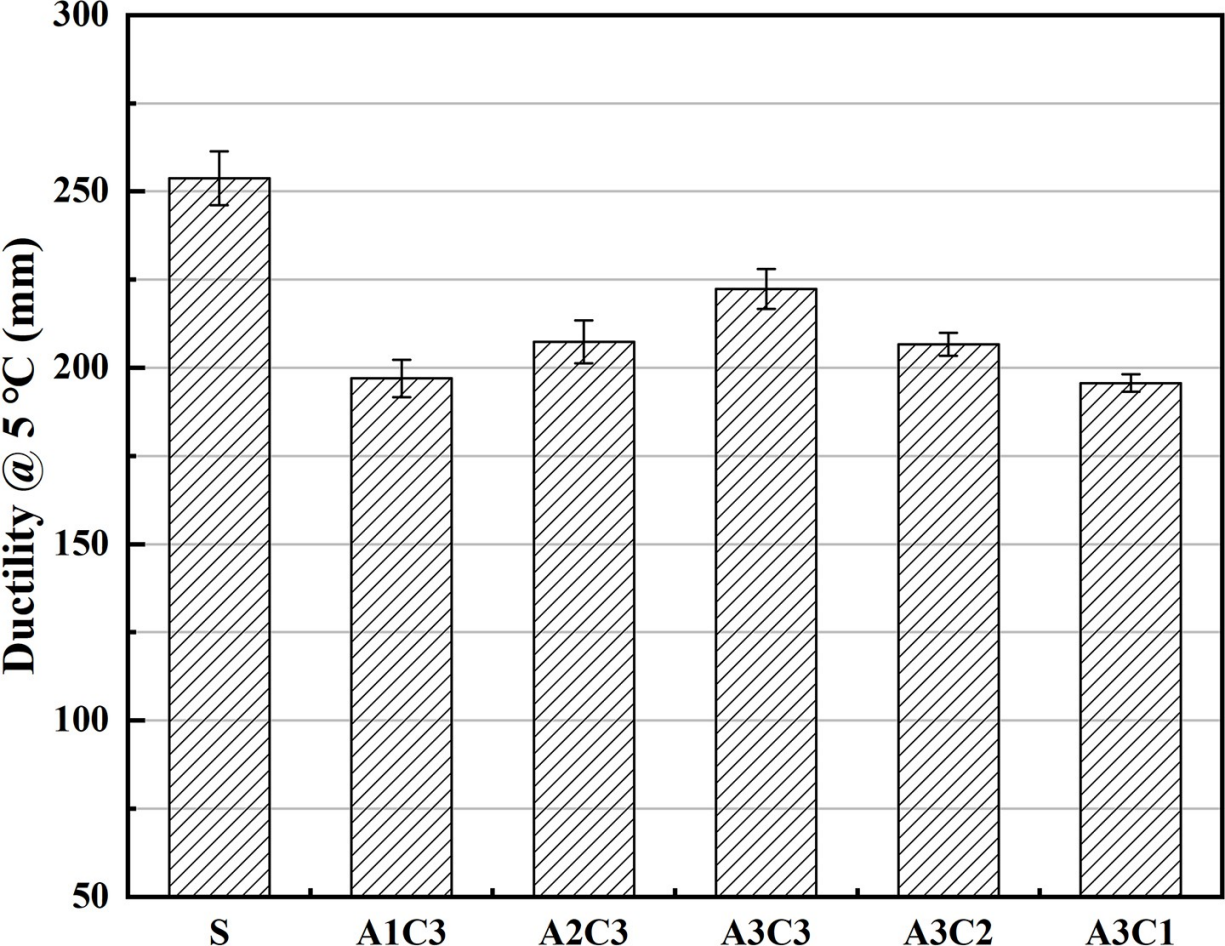
Differences in ductility.

As shown in Figs 1–3, the hardness, softening point and ductility of AAT nanomaterial modified asphalt were improved after the addition of CRP. Regardless of the amount of SBS or CRP added, the physical qualities of the modified asphalt improved, with increasing values as the doses went up. With SBS, at a doping level of 3.5%, the modified asphalt was significantly improved in terms of its hardness and softening point. Although the addition of CRP to the asphalt did not produce as great a performance boost as SBS, it met expectations. The AAT-composite-modified asphalt binders functioned best overall when the asphalt contained both 3.5% SBS and 3% CRP (i.e., A3C3). In terms of penetration, A3C3 outperformed SBS by over 16.4%, indicating that A3C3 has a higher hardness than SBS, which led to A3C3’s superior HT performance. The change in the softening point indicates that A3C3 improved the asphalt’s performance by almost 3.5% compared to SBS, demonstrating that A3C3 has superior HT characteristics and softness to SBS and confirming the hardness hypothesis. Although there was still a difference in ductility between A3C3 and SBS, both had a ductility of more than 220 mm at LT. In terms of fundamental physical qualities, the asphalt modified with CRP demonstrated increased quality, while this improvement was not as evident as that seen with the addition of SBS [41]. Simultaneously, a comparison between the modified asphalts shows that the impact of the addition of 3% CRP is physically equivalent to that of 1% SBS [42–44].

### 4.2. Rotational viscosity

**Fig 4:**
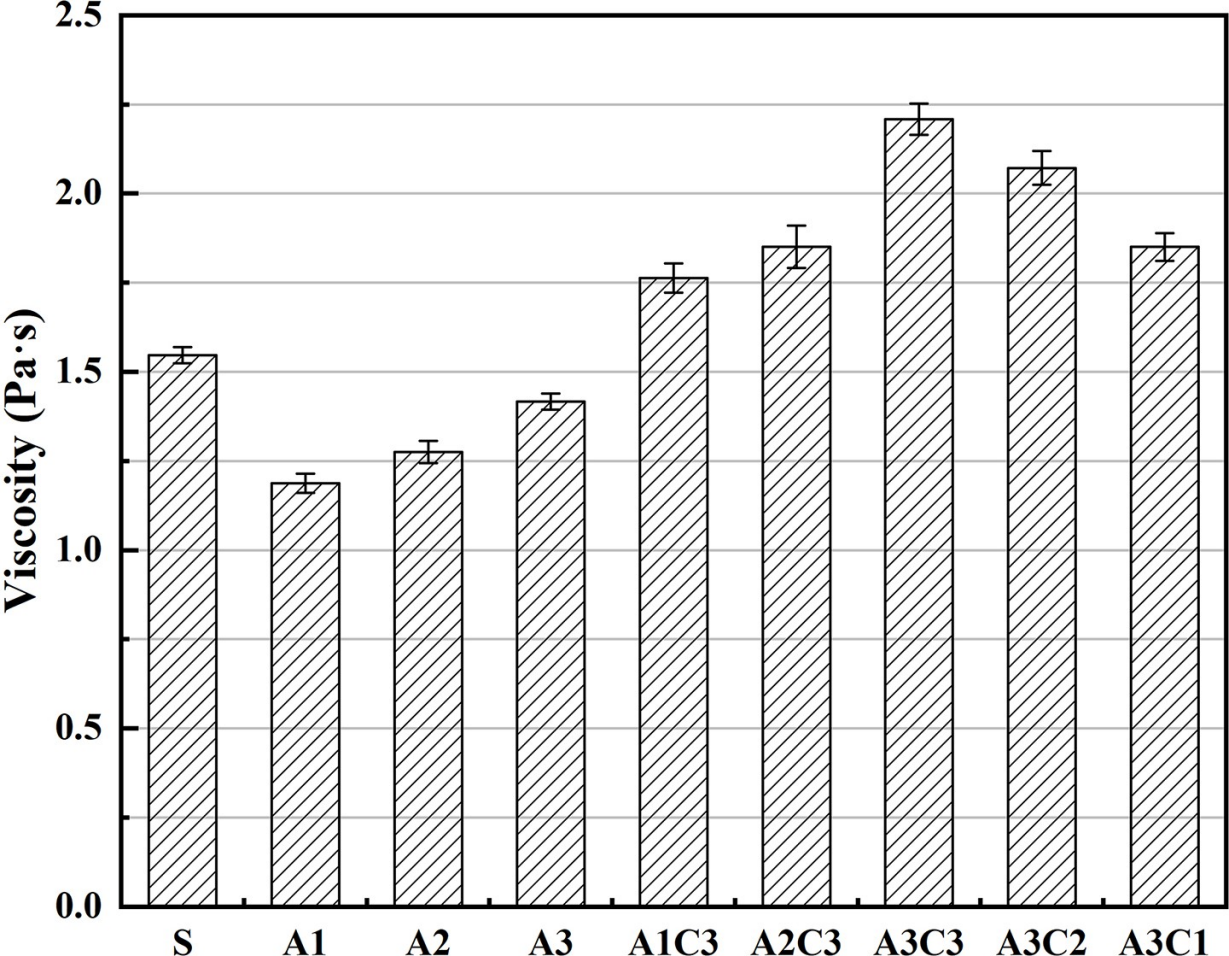
Difference in viscosity.

The viscosity of all modified asphalts increased with the addition of CRP, as shown in Fig 4. The viscosity of asphalt A1–A3 increased by an average of roughly 50.6% following CRP addition at 3% CRP; at 3.5% SBS, the average improvement in asphalt viscosity per 1% CRP was approximately 19.4%. A3C3 still maintained the maximum viscosity at a value of less than 3Pa·s, which fulfilled the project’s mixing criteria. The high viscosity of A3C3 implies that it has strong HT characteristics, which is consistent with the softening point data. The HT performance of asphalt has a direct effect on the road performance of the mixture, and the HT rutting resistance of the mixture performance is strongly linked to the HT performance of asphalt [45]. Good asphalt consistency helps in its subsequent mixing with the aggregates and other components and improves the binding of materials within the mix. The fact that a good viscosity may coexist with strong HT performance indicates that A3C3 has greater promise than SBS at the mix level.

### 4.3. Dynamic Shear Rheometer (DSR)

**Fig 5:**
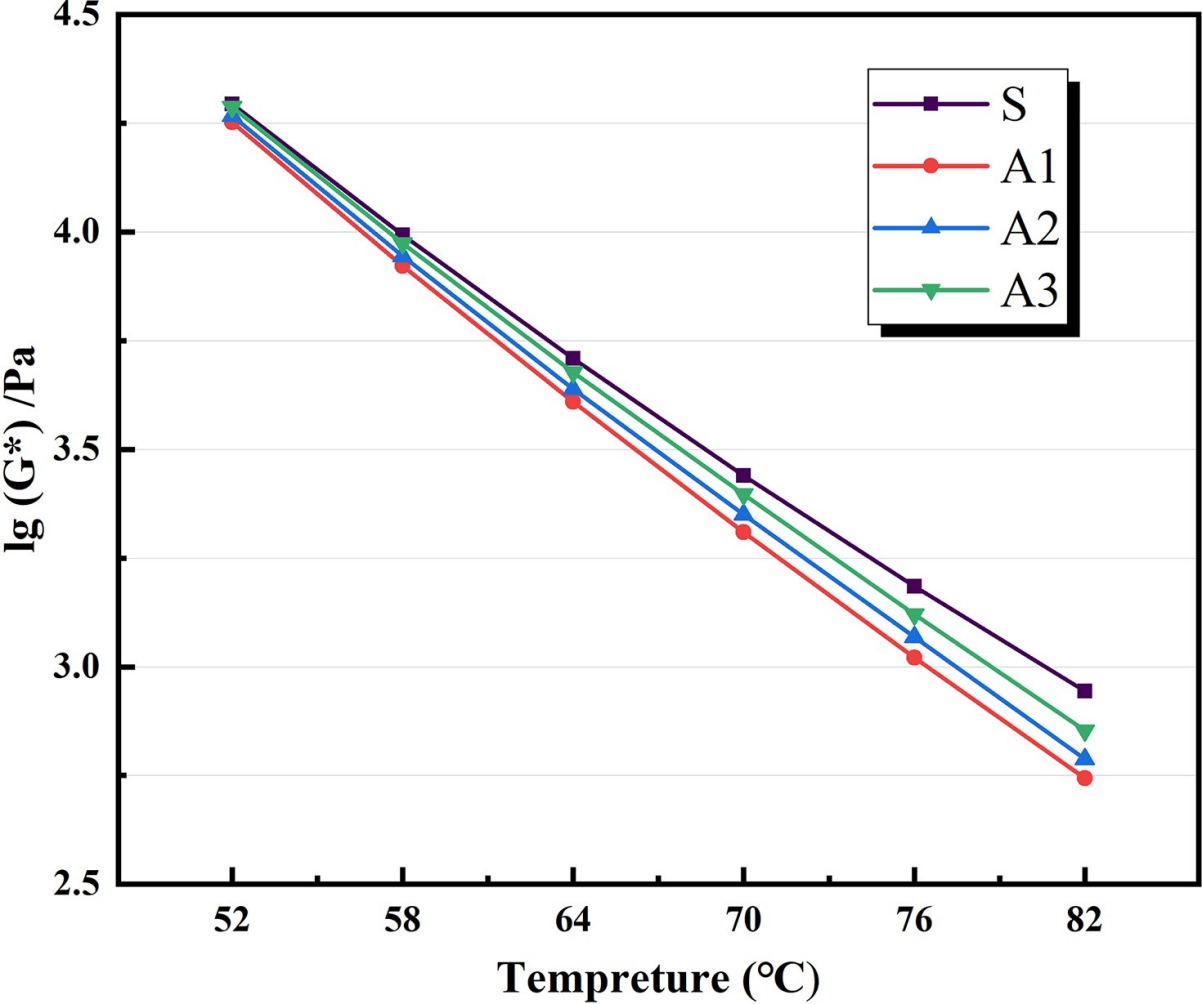
Complex modulus without CRP.

**Fig 6:**
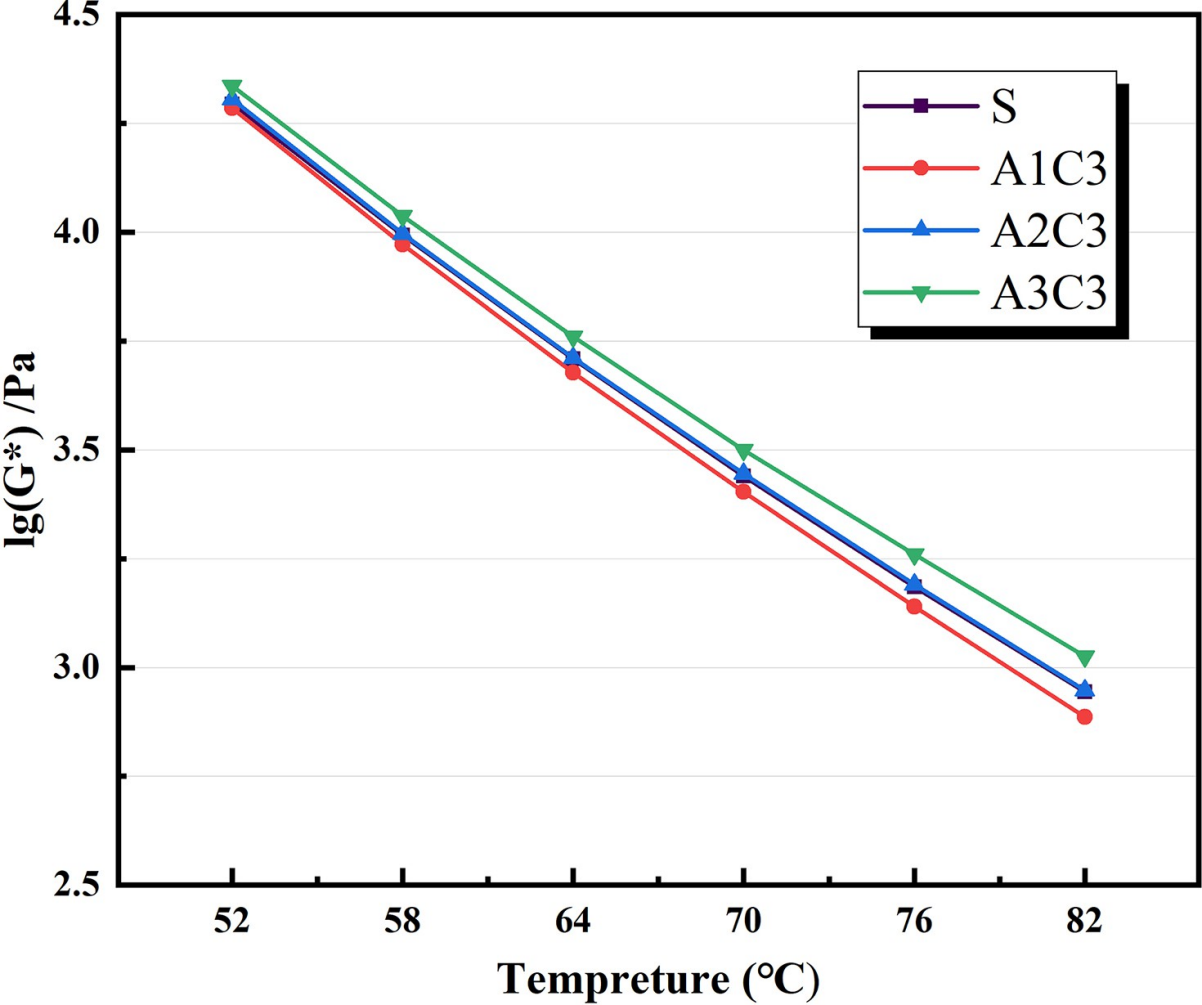
Complex modulus for SBS variations.

**Fig 7:**
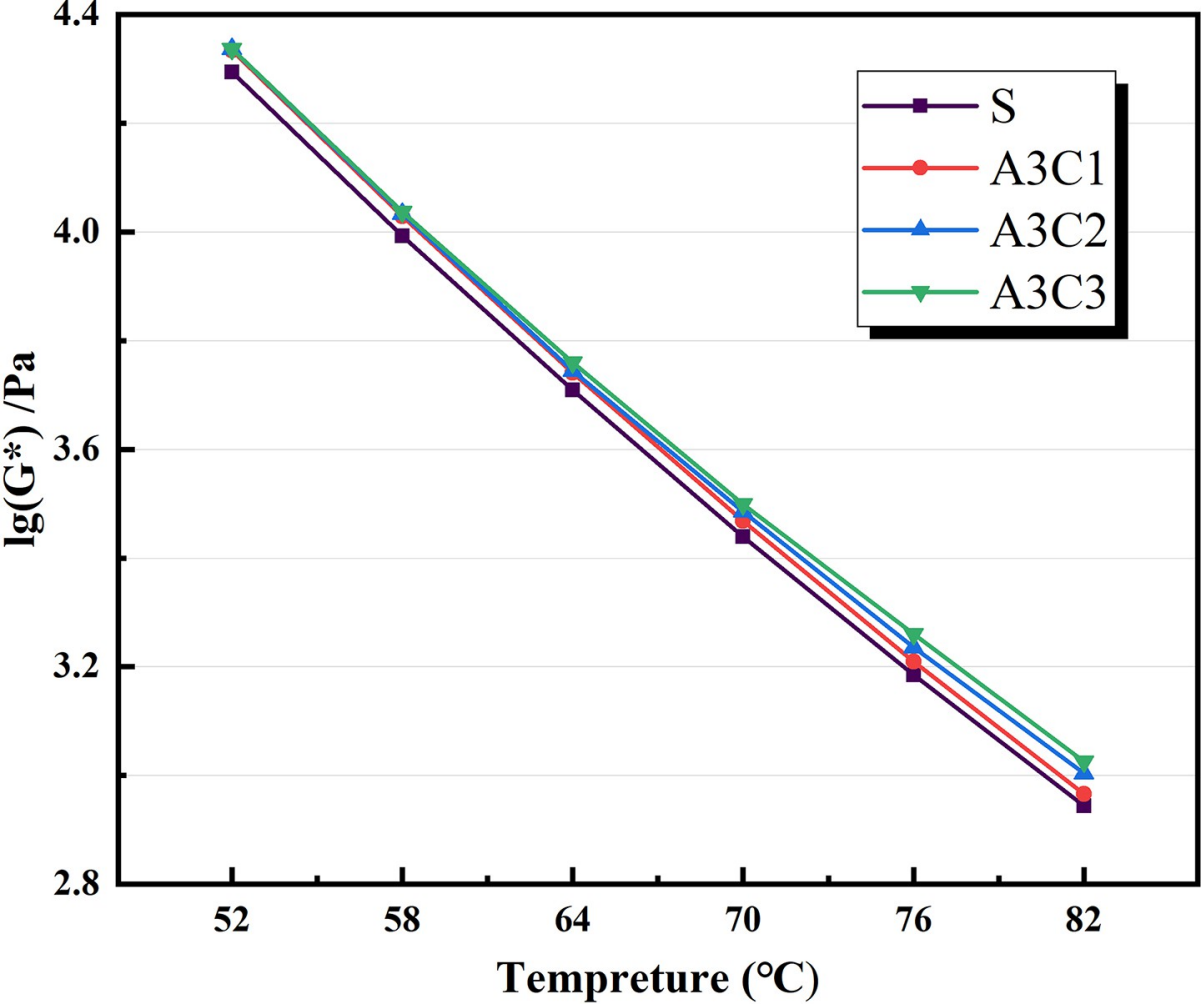
Complex modulus for CRP variations.

**Fig 8:**
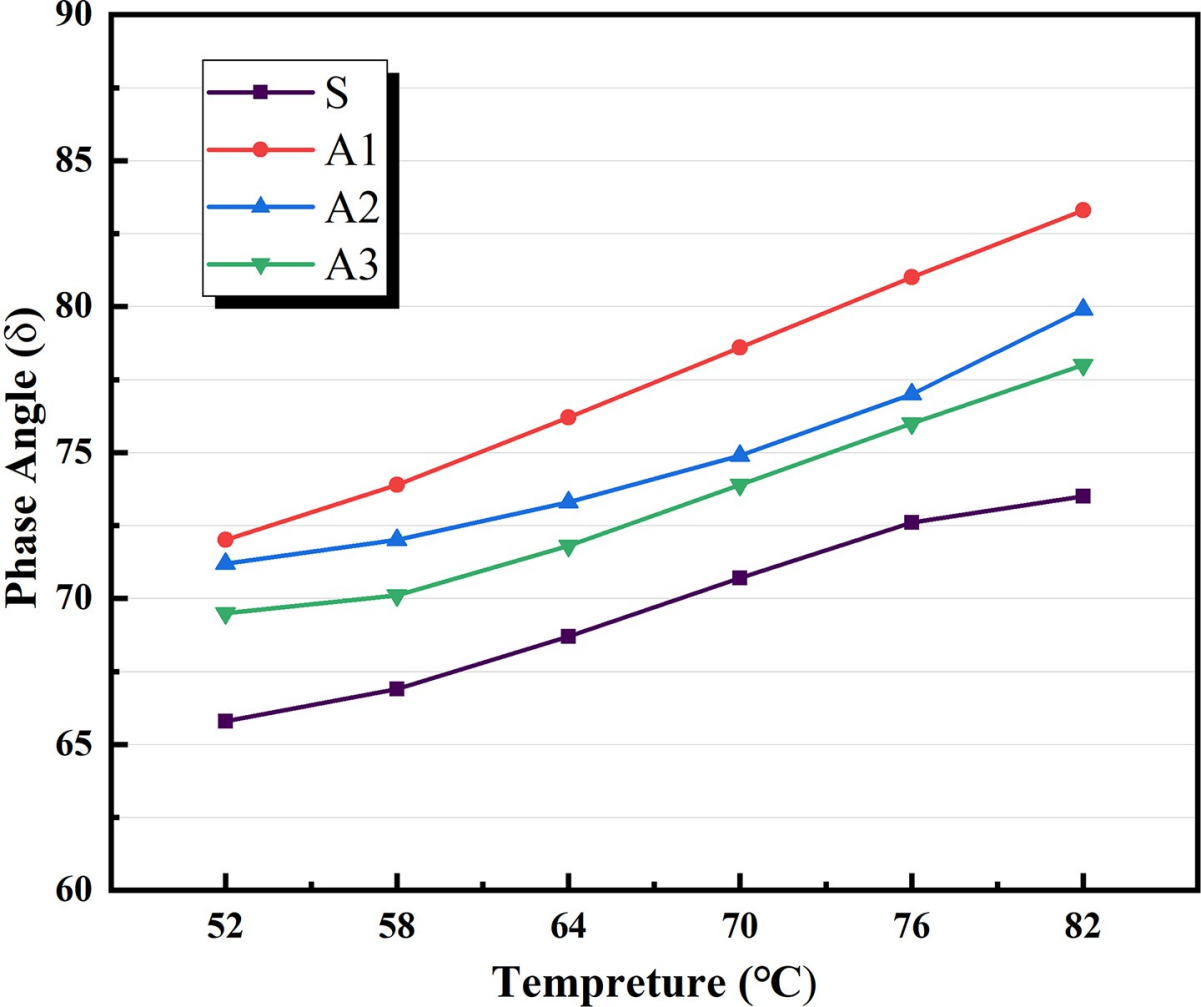
Phase angle without CRP.

**Fig 9:**
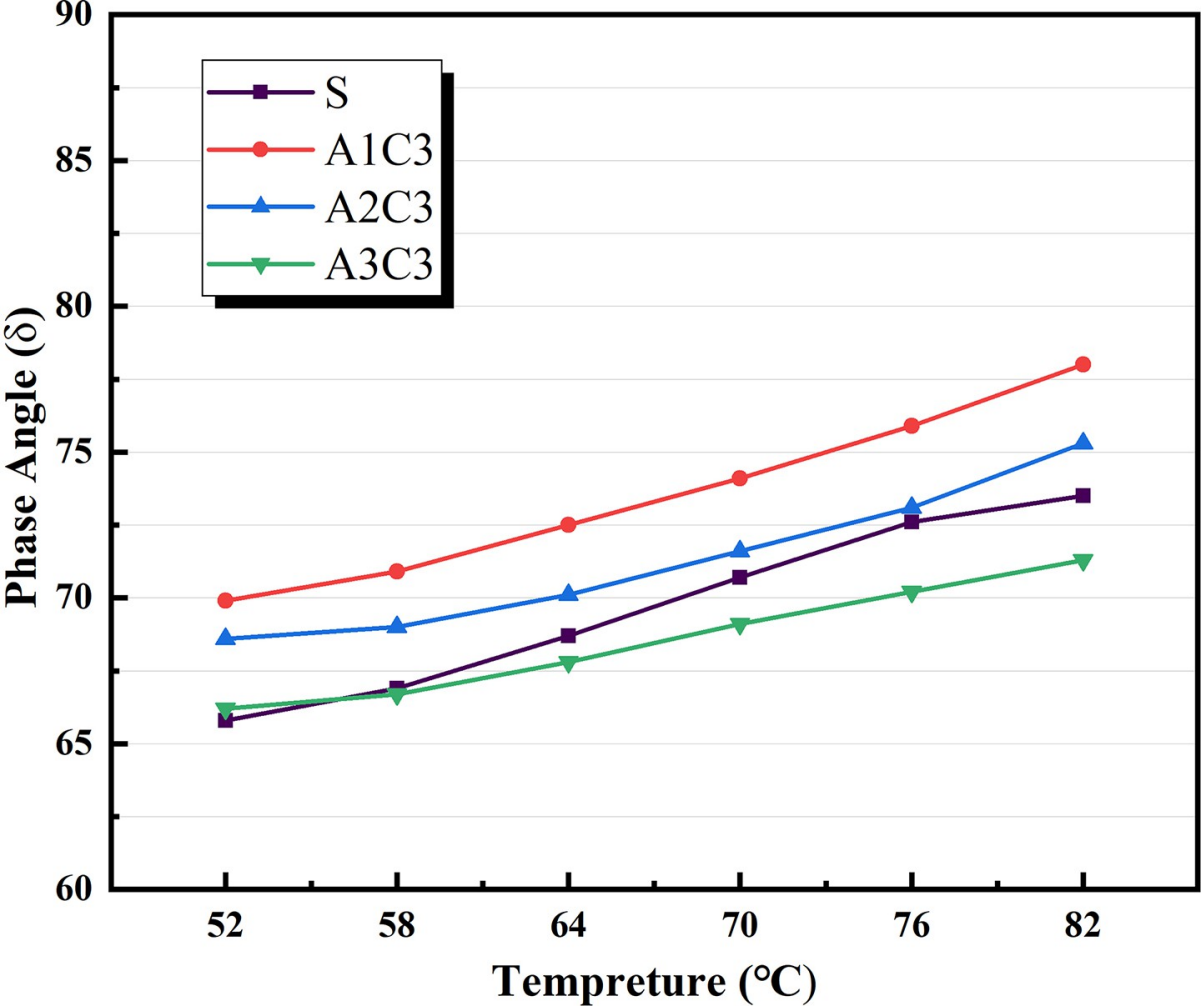
Phase angle for SBS variations.

**Fig 10:**
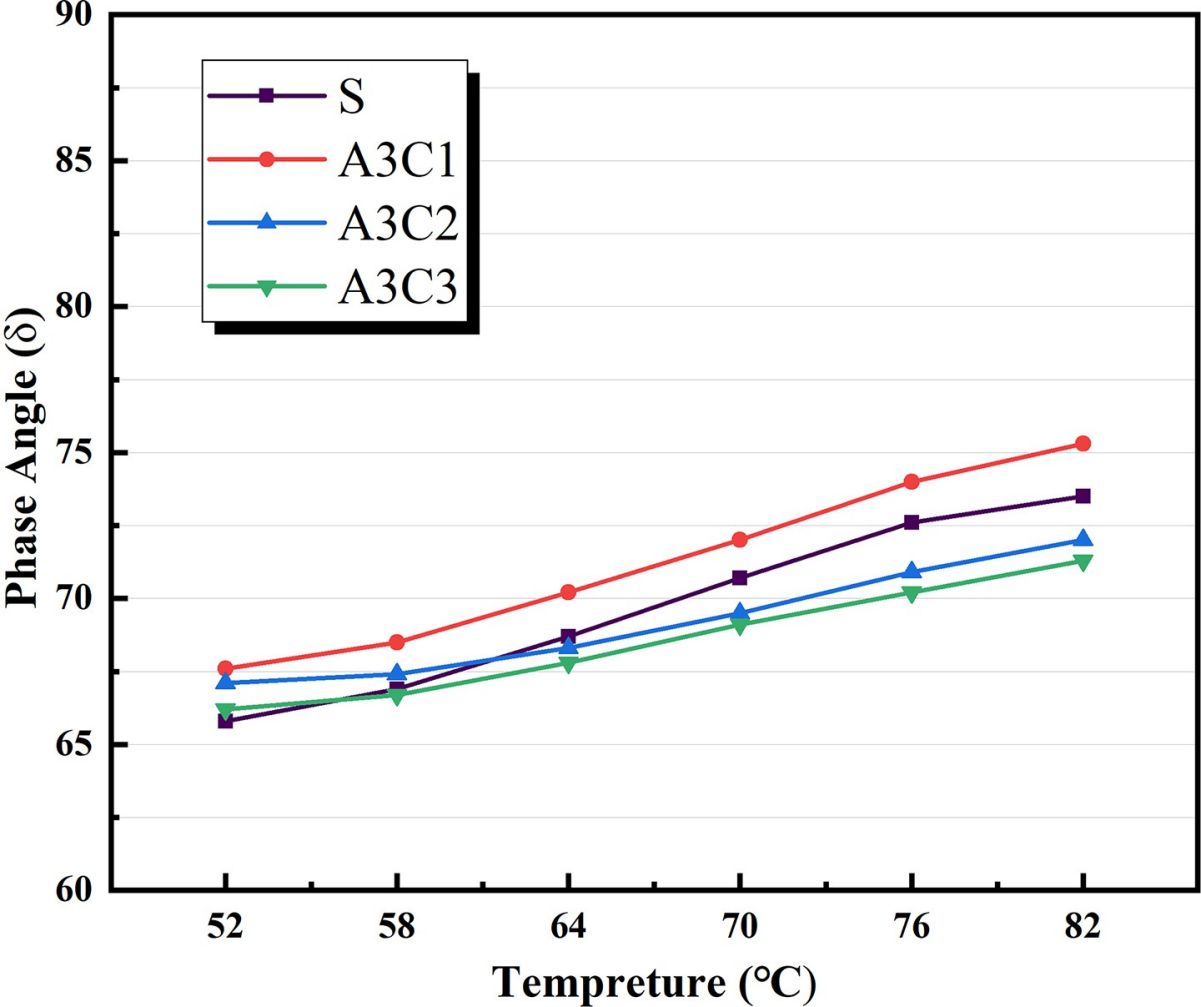
Phase angle for CRP variations.

**Fig 11:**
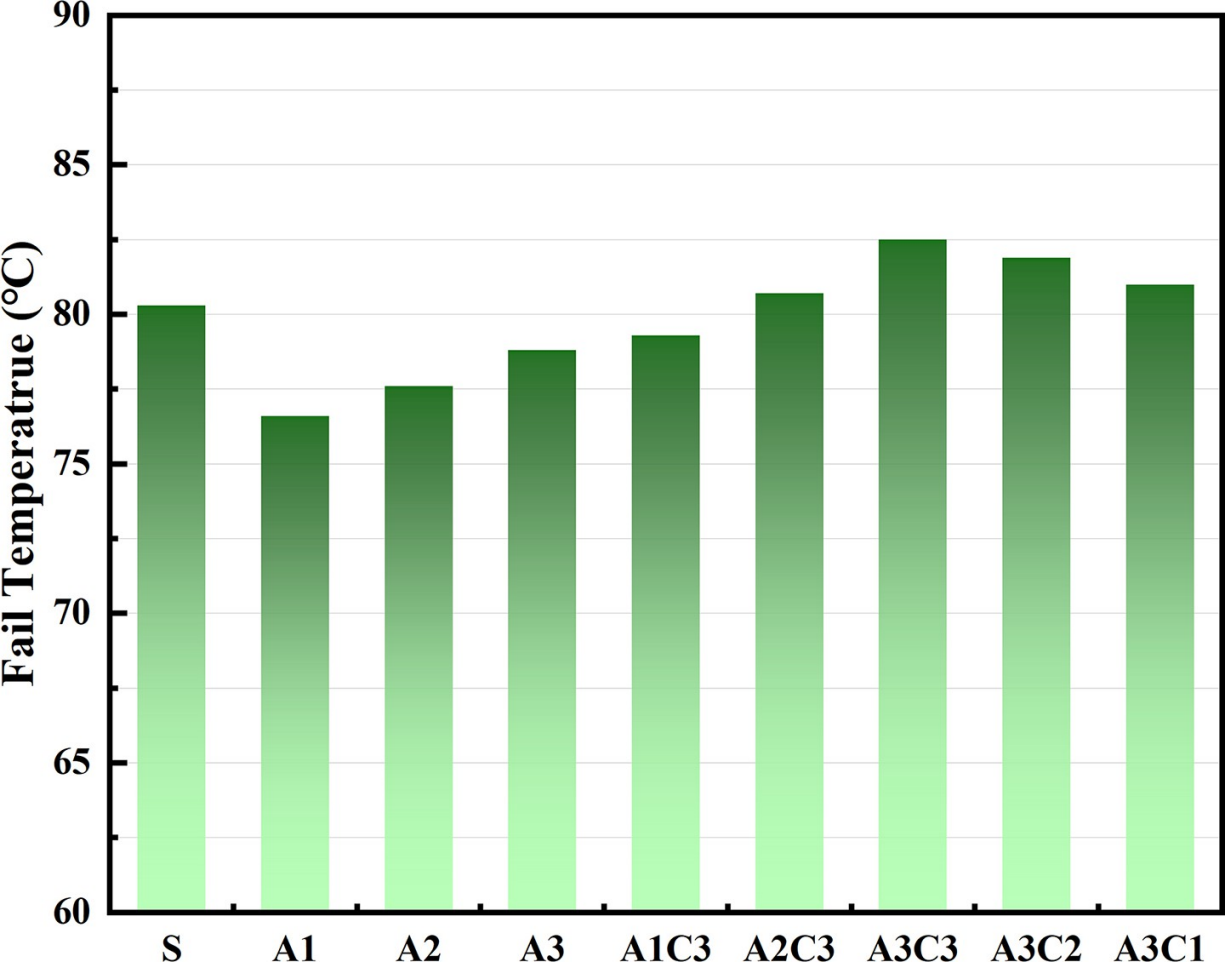
HT failure temperature of each asphalt binder.

Based on the physical attributes listed above, a preliminary assessment of the AATnanomaterial modified asphalt was made: the HT performance of the AAT nanomaterial modified asphalt was gradually improved by the addition of CRP. Figs 5–7 show how the complex modulus (G*) of different modified asphalts changes with temperature. It was found that A3C3 had the highest complex modulus (G*) at all temperatures. One of the most critical factors in determining asphalt materials’ HT rutting resistance is their complex modulus. A3C3 had a larger complex modulus (G*) than SBS, indicating that it can improve asphalt resistance to HT rutting deformation. The phase angles (δ) for several modified asphalts are shown in Figs 8–10. The stronger the viscoelastic responsiveness and stability performance of the asphalt material, the narrower the phase angle (δ) [46]. According to the graph, A3C3 had the shortest phase angle (δ) and was smaller than SBS, showing that A3C3 has better viscoelastic qualities than SBS. In terms of the complex modulus (G*) and phase angle (δ), A3C3 outperformed SBS at a HT. At the same time, CRP was still less efficient than SBS in terms of the magnitude of the improvement in HT performance. In terms of the complex modulus (G*), the mechanism by which the 1% SBS and 3% CRP were thought to perform at HT was confirmed [43]. The HT failure temperatures for each asphalt material are depicted in Fig 11, as determined by the Superpave standard for asphalt with a rutting factor greater than 1.0 kPa prior to aging. This indicator plays an important role in evaluating the HT aspects of modified asphalt in PG grades. The graph clearly shows that the failure temperature of A3C3 exceeded 82°C, whereas that of SBS was only 80°C, demonstrating that A3C3-modified asphalt has a higher HT rating than SBS-modified asphalt.

### 4.4. Bending Beam Rheometer (BBR)

**Fig 12:**
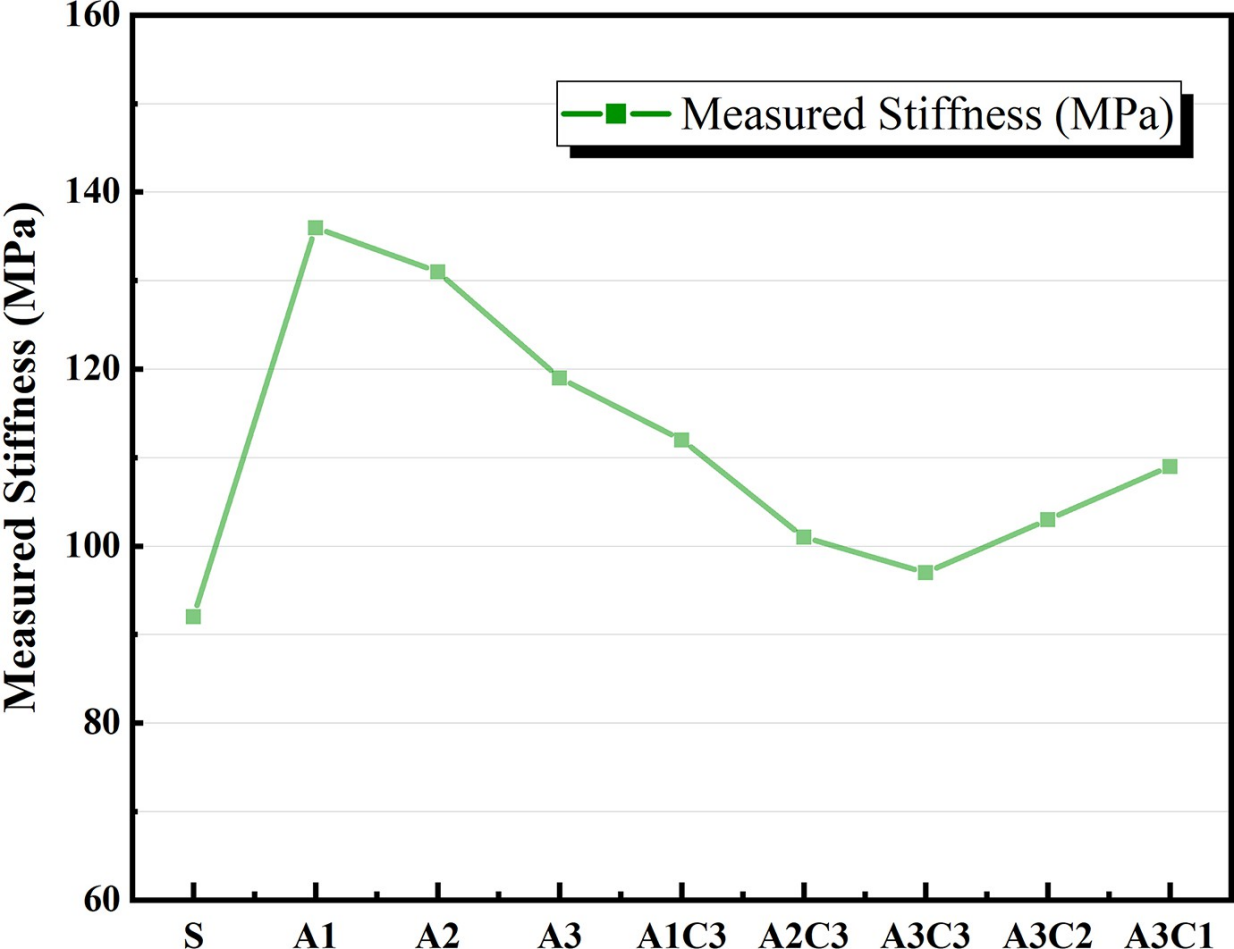
Measured Stiffness at −12°C.

**Fig 13:**
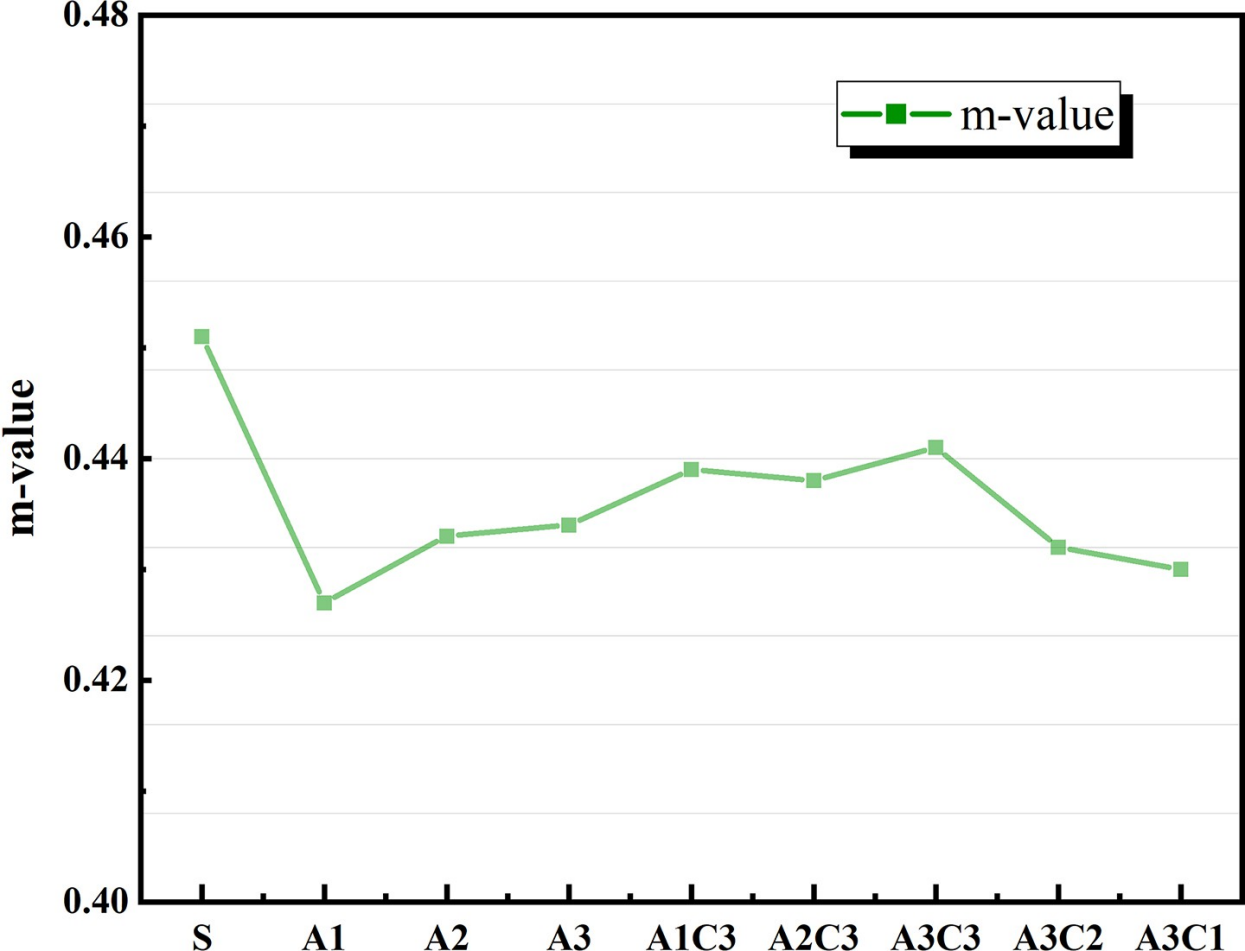
m-value at −12°C.

**Fig 14:**
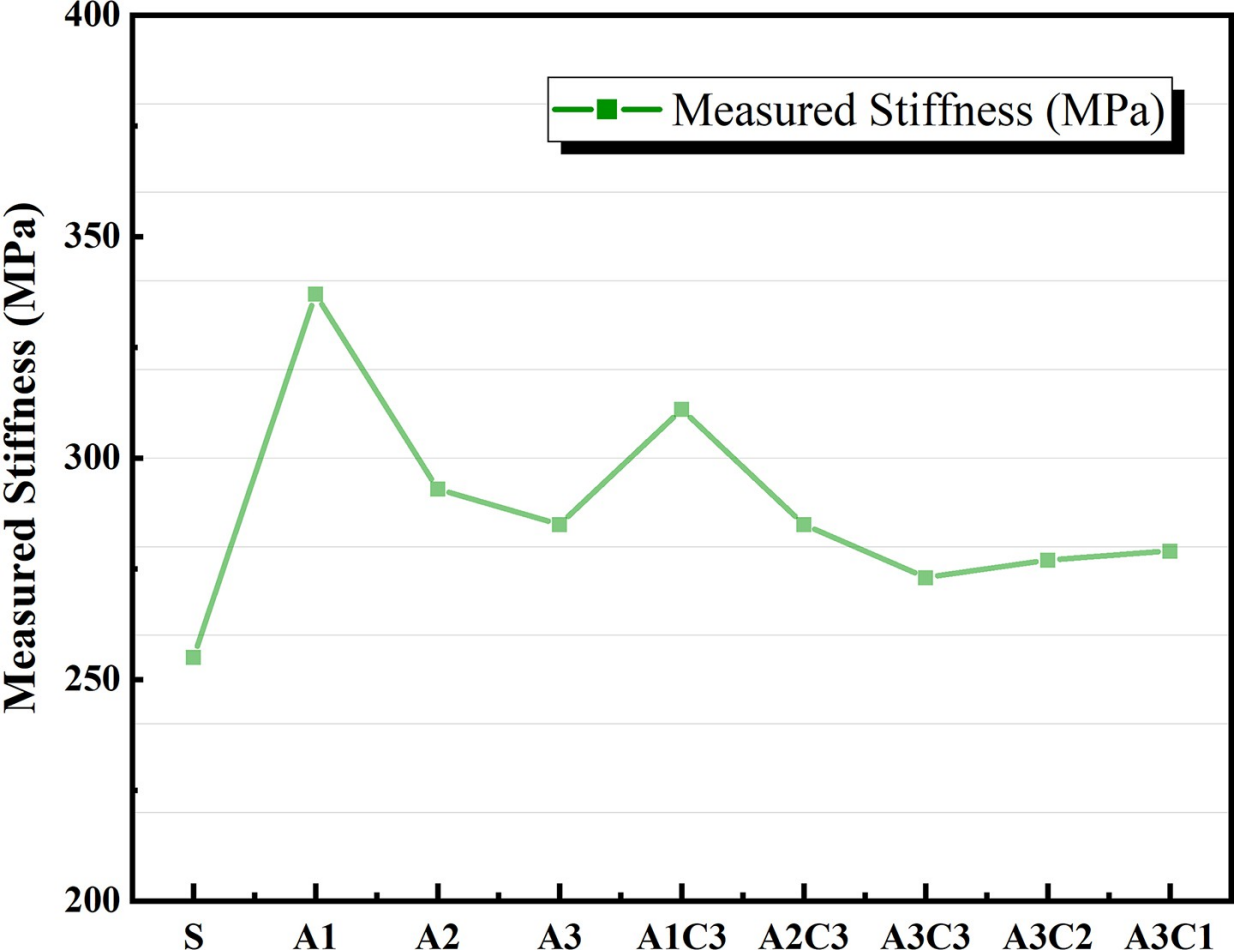
Measured Stiffness at -18°C.

**Fig 15:**
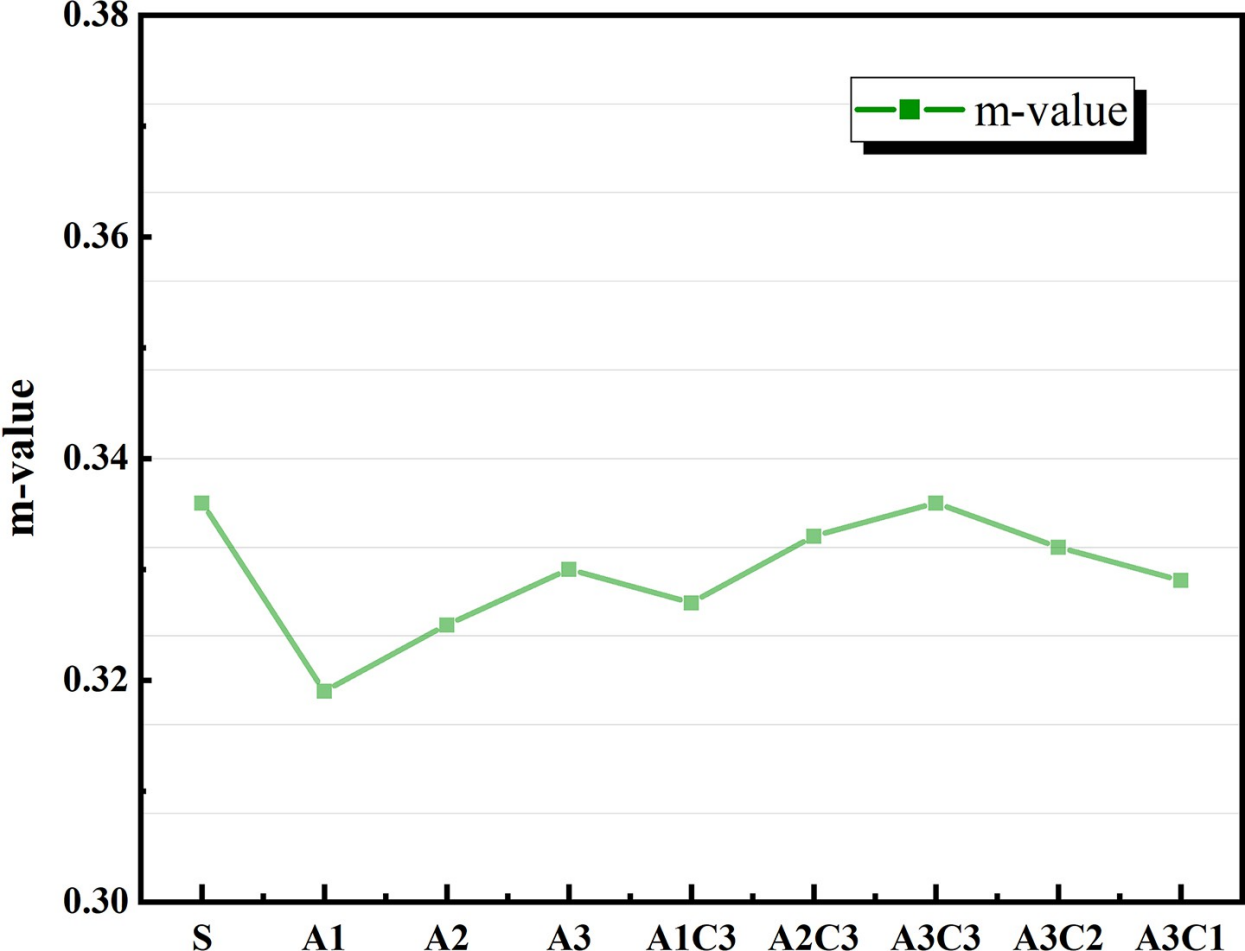
m-value at -18°C.

The variation in asphalt materials’ LT creep modulus (S) and creep rate (m value) is connected to the material’s capacity to withstand LT cracking during pavement usage. When S is large and m is small, the material’s brittle properties are more pronounced, and it is more susceptible to cracking damage [47]. Figs 17 and 18 show that increasing the quantity of SBS alone does not fulfill the performance criteria when compared to SBS-modified asphalt without CRP. The total LT creep stiffness modulus S of the AAT nanomaterial modified asphalt was lowered, and the m value was enhanced with the addition of CRP [48], as shown in Figs 12–15. According to the graph data, AAT-modified asphalts had the lowest LT creep stiffness modulus (S) and the maximum creep rate (m value), and hence the best LT cracking resistance. However, when A3C3 and SBS are similarly compared, SBS-modified asphalt still performs better at an LT than A3C3, whether the temperature is −12°C or −18°C. The BBR test findings for A3C3 and SBS are consistent with the judgments made for both during the LT ductility test.

### 4.5. Rolling Thin-Film Oven Test (RTFOT)

The quality of samples before and after short-term aging was assessed to determine the quality loss in the two modified asphalts during heat and oxygen aging. The less mass loss there is, the higher the aging resistance of the modified asphalt is [49]. The mass loss of A3C3 was smaller than that of SBS, as seen in Table 8. The A3C3-modified asphalt outperformed the SBS-modified asphalt in terms of aging resistance.

**Table 8:** Quality loss after RTFOT aging.

Materials	Mass Loss (%)			
	1#	2#	3#	Average
S	0.39	0.37	0.56	0.44
A3C3	0.23	0.23	0.37	0.28

Note: Superpave specification for maximum mass loss = 1%.

### 4.6. Multiple-Stress Creep Recovery (MSCR)

**Fig 16:**
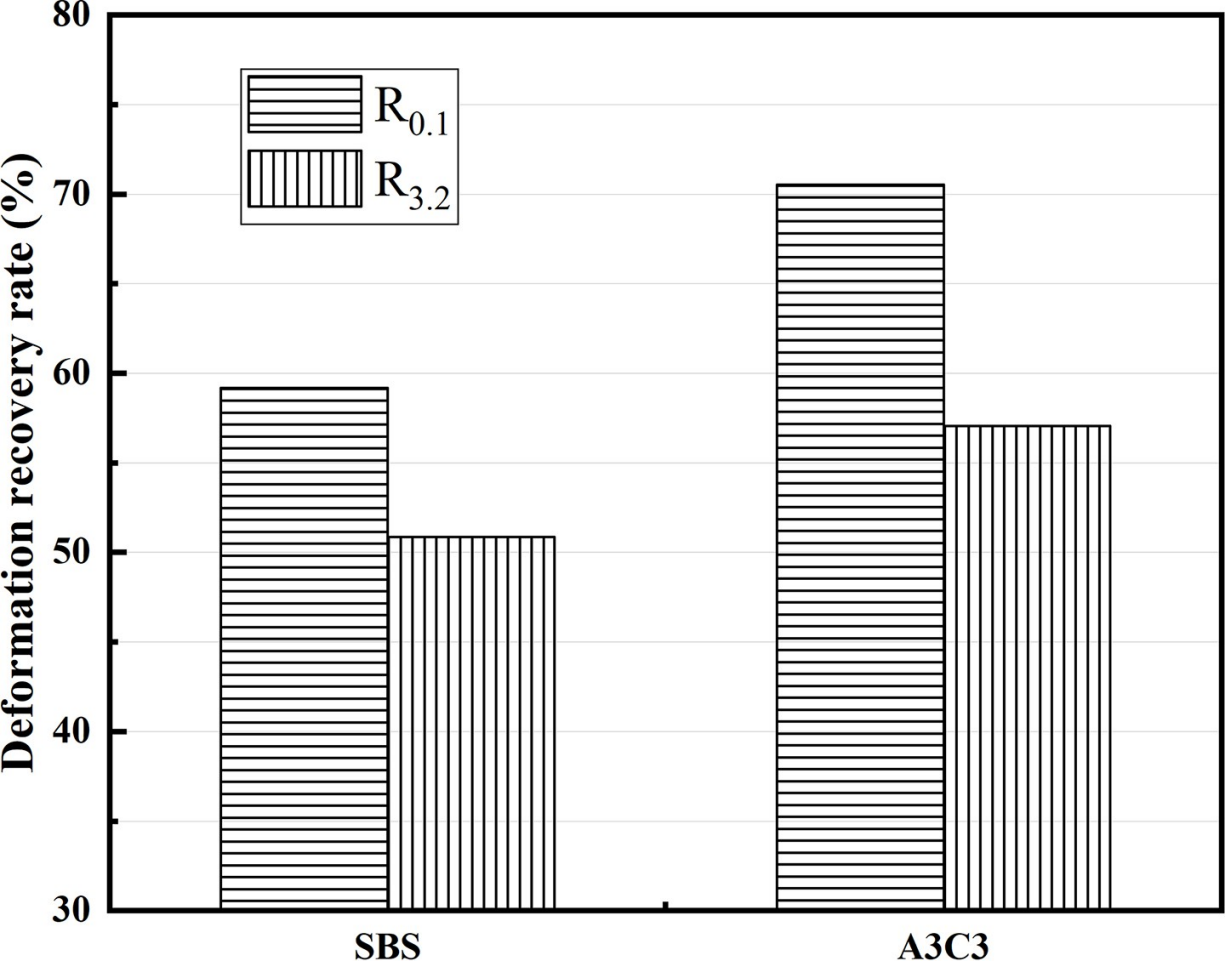
Deformation recovery rate.

**Fig 17:**
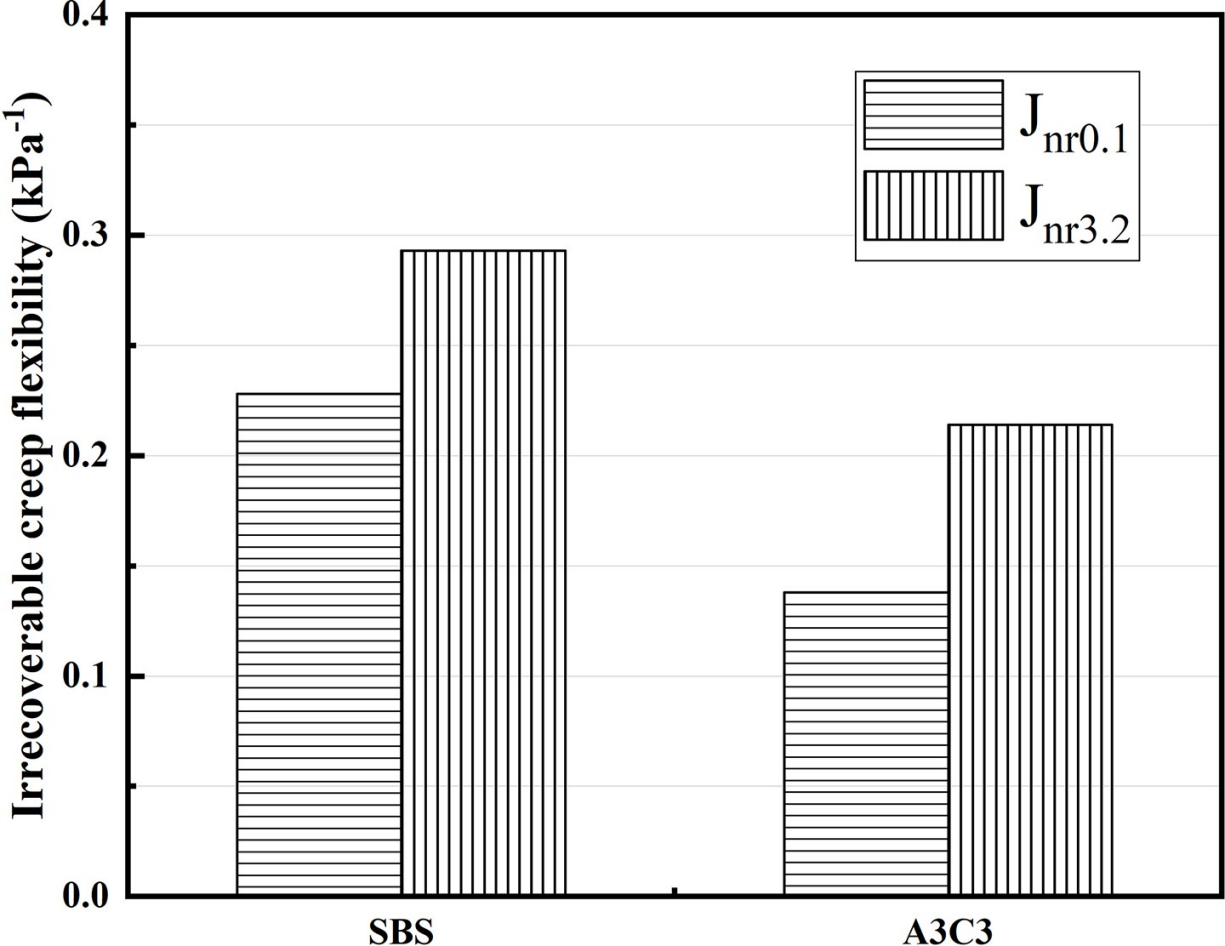
Irrecoverable creep flexibility.

MSCR experiments on SBS-modified asphalt and A3C3-modified asphalt after short-term aging were carried out to investigate their HT creep characteristics. By comparing the deformation recovery rate (R) and irrecoverable creep flexibility (J_nr_) produced under stress conditions of 0.1 kPa and 3.2 kPa, the variations in the viscoelastic properties of the two modified asphalts were explored. As demonstrated in Fig 16, A3C3 had a higher R0.1 and R3.2 than SBS, and as the recovery rate R grew, the elastic properties of this asphalt material became more obvious, demonstrating that it can still maintain superior elastic recovery when exposed to HTs [46]. The J_nr0.1_ and Jnr_3.2_ of A3C3 were both smaller than those of SBS, as illustrated in Fig 17. The lower the irreversible creep flexibility J_nr_, the less irreversible deformation there was in this asphalt material, enhancing its lack of excessive deformation at HT, which implies that A3C3 has superior HT deformation resistance. The R_diff_ of A3C3 was 19.1% and J_nrdiff_ was 55.2%. The R_diff_ of SBS is 14.1% and J_nrdiff_ is 28.2%. The elasticity of A3C3 is more significant than SBS. When it comes to HT stability performance, A3C3 still had the edge, which is in line with what the DSR results show.

### 4.7. Fourier Transform Infrared Spectroscopy (FTIR)

**Fig 18:**
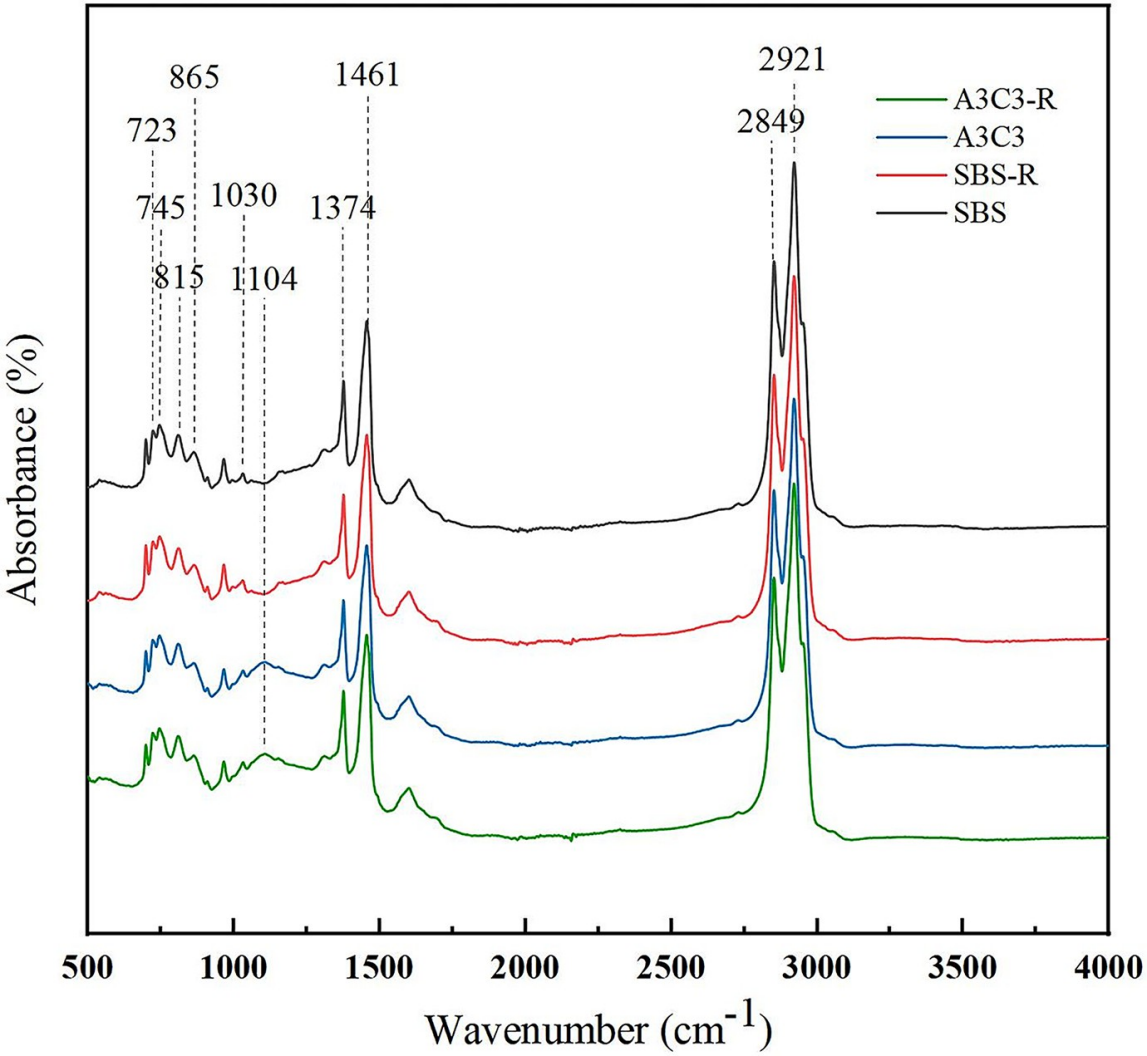
Spectrum of asphalt binder before and after aging.

**Fig 19:**
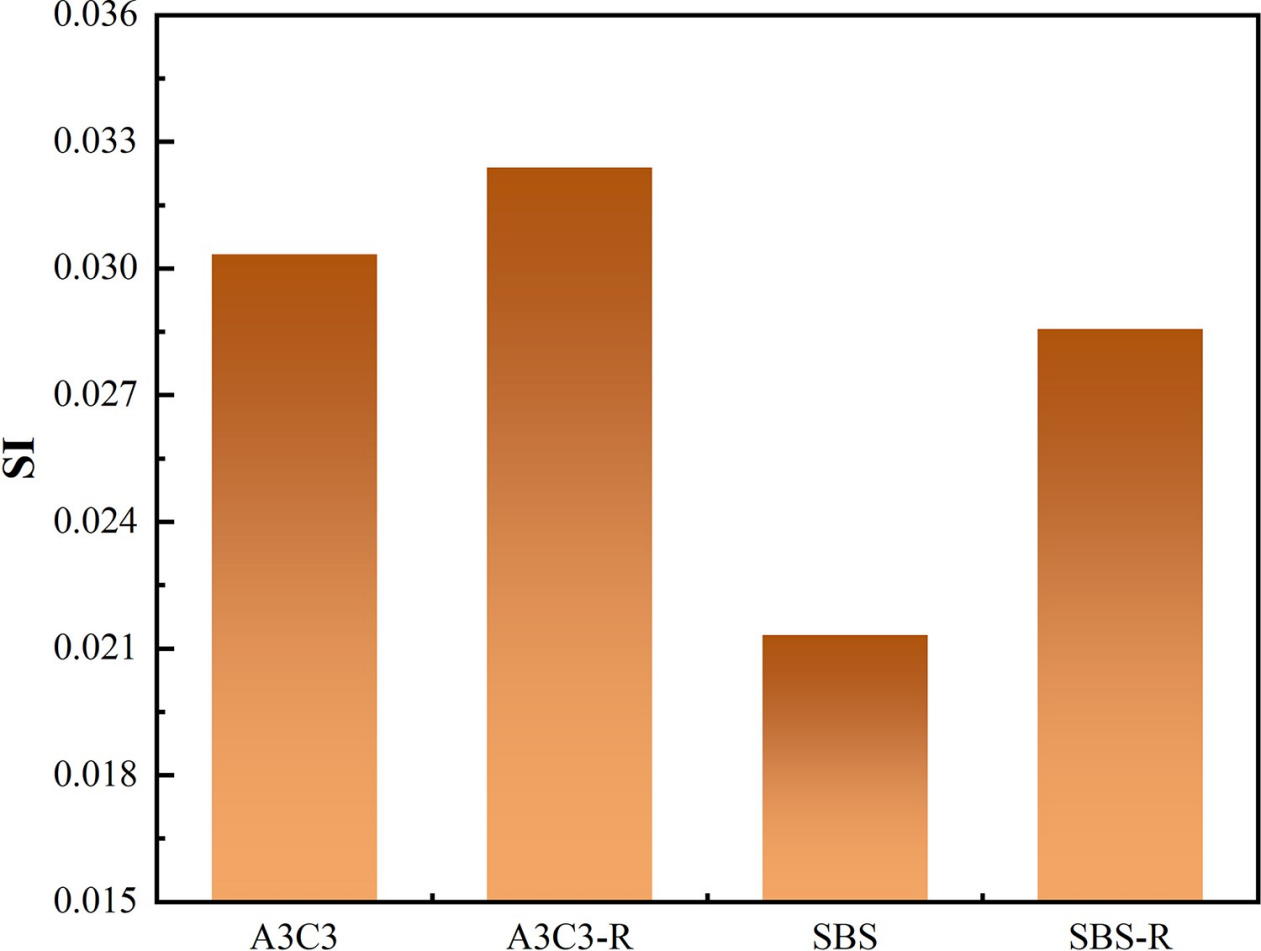
Functional group index of asphalt binder before and after aging.

Both SBS-modified asphalt and A3C3-modified asphalt exhibited strong absorption peaks at 2921 cm^−1^, 2849 cm^−1^, 1600 cm^−1^, 1461 cm^−1^, 1374 cm^−1^, 865 cm^−1^, 815 cm^−1^, 745 cm^−1^ and 723 cm^−1^, as shown in Fig 18. The antisymmetric and symmetric stretching vibrations of the saturated hydrocarbon (-CH_2_-) had significant absorption peaks at 2921 cm^−1^ and 2849 cm^−1^, respectively. The (C = C) stretching vibration of dense-ring aromatic hydrocarbons was 1600 cm^−1^, the shear vibration of methylene (-CH_2_-) was 1461 cm^−1^, and the antisymmetric and symmetric bending vibration of methyl was 1374 cm^−1^ (-CH_3_) [50]. The fingerprint area absorption peaks at 865 cm^−1^ and 815 cm^−1^ are distinctive groups of an aromatic chemical class, generating a (= CH) out-of-plane curved vibrational band. However, the absorption peaks at 745 cm^−1^ and 723 cm^−1^ were caused by the concerted vibration of the methylene chain segment (-(CH_2_)*n*-, (*n* ≥ 4)), confirming the existence of long-chain alkanes [51,52]. Whereas the absorption peak unique to A3C3 with respect to SBS was found at 1104 cm^−1^, the absorption peak of the intensity shift in question was at 1104 cm^−1^. The fingerprint area lies in the low-frequency range of the infrared spectrum, from 1300 cm^−1^ to 650 cm^−1^, and is created by bending vibrations in the (= CH) plane on the aromatic ring. It can be clearly found from Fig 19 that the SI of A3C3 modified asphalt did not change much after thermal oxygen aging. In contrast, the SI index of SBS modified asphalt changes obviously. The SI of A3C3 increased by 6.8% and SBS by 34%. This also shows that the anti-aging performance of A3C3 modified asphalt is better than that of SBS modified asphalt.

## 5. Conclusion

The purpose of this research was to examine the performance changes that occur when CRP is added to AAT nanomaterial modified asphalt. For relevant performance tests, methods for measuring physical properties, testing rheological properties and testing at the microscopic level were used. Both nano-AAT-composite-modified asphalt and SBS-modified asphalt were tested so that the optimal nano-AAT-composite-modified asphalt solution could be chosen. Based on the results of the experimental tests, the following conclusions can be drawn:

1. At the basic physical property level, the addition of CRP to the AAT improved the penetration, softening point and ductility of the nanocomposite-modified asphalt, indicating that CRP improved the hardness, LT tensile capacity, softness and HT properties of the AAT-modified asphalt.
2. After the addition of CRP to the AAT, the complex modulus (G*) of the nanocomposite-modified asphalt increased and the phase angle (δ) was lowered to the HT performance level, and the rutting factor (G*/sinδ) of the nanocomposite-modified asphalt was subsequently enhanced. At the same time, CRP increased the deformation recovery rate (R) of the nanocomposite-modified asphalt and decreased the irrecoverable creep flexibility (J_nr_). This demonstrates that CRP successfully enhances AAT-treated asphalt’s HT rutting resistance and resistance to HT deformation.
3. After CRP was added to the AAT, the LT creep stiffness modulus S of the nanocomposite-modified asphalt decreased and the creep rate m increased. This demonstrates that CRP effectively supplements the cracking resistance of AAT-modified asphalt and increases toughness.
4. By performance comparison, A3C3 was chosen as the superior AAT/CRP-modified asphalt. When compared to SBS-modified asphalt, A3C3 had a higher hardness, softening point, rutting factor (G*/sinδ) and creep recovery, indicating that it performs better at high temperatures. However, A3C3 did not perform as well as SBS in terms of LT cracking resistance. The absorption curves of A3C3- and SBS-modified asphalt were almost identical in FTIR, with A3C3 having a single absorption peak at 1104 cm^−1^. Through the analysis of SI index, it is found that the thermal oxidative aging resistance of A3C3 is better than that of SBS.
5. The A3C3-modified asphalt (AAT-3.5%SBS-3%CRP) screened through the study had a lower price and better performance than that SBS-modified asphalt (4.5%SBS), and enriched and improved the content of modified asphalt, which is a significant finding for the study of modified asphalt. At the same time, there are far too many cost-effective projects to choose from.

## 6. Recommendations

This study combined CRP with AAT nanomaterial modified asphalt. In order to meet the engineering requirements, a better type of CRP can be selected with an optimum amount when added to the asphalt to achieve better LT performance of the modified asphalt.

## Supporting information

S1 Data (PDF)
