# Supplementary material for: Physical, rheological and microscopic properties of AAT nanomaterial/crumb rubber powder composite-modified asphalt and SBS-modified asphalt
Source: PLoS One. 2024 Jan 11;19(1):e0284813. doi: 10.1371/journal.pone.0284813 (PMC10783774; doi:10.1371/journal.pone.0284813)
Supplement: S1 Data — (PDF) [file pone.0284813.s001.pdf]

1、

Materials	25 °C Penetration (dmm)	Softening Point (°C)	5 °C Ductility (mm)	Viscosity (Pa·s)
SBS	50.8	67.7	255	1.547
A1	53.2	54.1	153	1.188
A2	51.5	56.2	185	1.275
A3	50.1	57.3	191	1.417
A3C1	48.9	64.9	196	1.851
A3C2	45.6	67.1	206	2.072
A3C3	42.5	69.2	222	2.208
A2C3	45.7	60.3	207	1.851
A1C3	49.8	57.0	197	1.763

2、DSR

T/G*	SBS	A1	A2	A3
52	19.7	17.9	18.5	19.4
58	9.84	8.35	8.8	9.42
64	5.12	4.07	4.35	4.76
70	2.75	2.04	2.24	2.49
76	1.53	1.05	1.17	1.32
82	0.88	0.554	0.613	0.714

T/G*	SBS	A3C1	A3C2	A3C3
52	19.7	21.6	21.7	21.7
58	9.84	10.7	10.8	10.9
64	5.12	5.51	5.56	5.76
70	2.75	2.94	3.06	3.16
76	1.53	1.62	1.72	1.82
82	0.88	0.924	1.01	1.06

T/G*	SBS	A1C3	A2C3	A3C3
52	19.7	19.3	20.2	21.7
58	9.84	9.38	9.91	10.9
64	5.12	4.76	5.14	5.76
70	2.75	2.53	2.79	3.16
76	1.53	1.38	1.55	1.82
82	0.88	0.77	0.887	1.06

T/ δ	SBS	A1	A2	A3

52	65.8	72	71.2	69.5
58	66.9	73.9	72	70.1
64	68.7	76.2	73.3	71.8
70	70.7	78.6	74.9	73.9
76	72.6	81	77	76
82	73.5	83.3	79.9	78

T/ δ	SBS	A3C1	A3C2	A3C3
52	65.8	67.6	67.1	66.2
58	66.9	68.5	67.4	66.7
64	68.7	70.2	68.3	67.8
70	70.7	72	69.5	69.1
76	72.6	74	70.9	70.2
82	73.5	75.3	72	71.3

T/ δ	SBS	A1C3	A2C3	A3C3
52	65.8	69.9	68.6	66.2
58	66.9	70.9	69	66.7
64	68.7	72.5	70.1	67.8
70	70.7	74.1	71.6	69.1
76	72.6	75.9	73.1	70.2
82	73.5	78	75.3	71.3

	Fail Temperatrue (℃)
S	80.3
A1	76.6
A2	77.6
A3	78.8
A1C3	79.3
A2C3	80.7
A3C3	82.5
A3C2	81.9
A3C1	81

3、BBR

-12℃	Measured Stiffness (MPa)	m-value
S	92	0.451
A1	136	0.427
A2	131	0.433

A3	119	0.434
A1C3	112	0.439
A2C3	101	0.438
A3C3	97	0.441
A3C2	103	0.432
A3C1	109	0.43

-18℃	Measured Stiffness (MPa)	m-value
S	255	0.336
A1	337	0.319
A2	293	0.325
A3	285	0.33
A1C3	311	0.327
A2C3	285	0.333
A3C3	273	0.336
A3C2	277	0.332
A3C1	279	0.329

4、RTFOT

Materials	Mass Loss (%)			
	1#	2#	3#	Average
S	0.39	0.37	0.56	0.44
A3C3	0.23	0.23	0.37	0.28

5、MSCR

Materials	64℃			
	R0.1	R3.2	Jnr0.1	Jnr3.2
SBS	59.19	50.86	0.228	0.293
A3C3	70.53	57.05	0.138	0.214

6、FTIR

Materials	SI
A3C3	0.03034
A3C3-R	0.03239
SBS	0.02132
SBS-R	0.02857

Wavenumber	SBS	SBS-R	A3C3	A3C3-R
4000	1.68	11.78	23.69	33.95
3999	1.67	11.78	23.7	33.94
3998	1.67	11.78	23.7	33.94
3997	1.66	11.78	23.7	33.93

3996	1.65	11.77	23.7	33.93
3995	1.65	11.77	23.7	33.93
3994	1.65	11.76	23.69	33.93
3993	1.65	11.76	23.69	33.93
3992	1.66	11.77	23.68	33.94
3991	1.67	11.77	23.68	33.94
3990	1.67	11.77	23.68	33.94
3989	1.66	11.77	23.69	33.94
3988	1.65	11.77	23.7	33.93
3987	1.64	11.77	23.7	33.93
3986	1.64	11.77	23.7	33.93
3985	1.64	11.77	23.7	33.93
3984	1.65	11.77	23.7	33.93
3983	1.66	11.77	23.7	33.94
3982	1.66	11.77	23.7	33.94
3981	1.67	11.77	23.7	33.95
3980	1.67	11.77	23.69	33.95
3979	1.66	11.76	23.69	33.95
3978	1.66	11.75	23.69	33.94
3977	1.65	11.75	23.69	33.93
3976	1.64	11.75	23.69	33.93
3975	1.63	11.75	23.69	33.93
3974	1.63	11.75	23.69	33.93
3973	1.63	11.75	23.68	33.94
3972	1.64	11.76	23.68	33.94
3971	1.64	11.76	23.69	33.93
3970	1.64	11.76	23.69	33.93
3969	1.64	11.75	23.69	33.92
3968	1.64	11.75	23.69	33.92
3967	1.64	11.75	23.69	33.93
3966	1.64	11.75	23.69	33.93
3965	1.65	11.76	23.69	33.93
3964	1.65	11.76	23.69	33.94
3963	1.65	11.76	23.68	33.94
3962	1.64	11.77	23.68	33.94
3961	1.64	11.77	23.68	33.94
3960	1.63	11.77	23.68	33.94
3959	1.63	11.76	23.68	33.94
3958	1.64	11.75	23.67	33.93
3957	1.64	11.75	23.67	33.93
3956	1.64	11.75	23.67	33.92
3955	1.65	11.75	23.67	33.92
3954	1.65	11.75	23.68	33.92
3953	1.65	11.75	23.68	33.92

3952	1.64	11.75	23.68	33.91
3951	1.64	11.75	23.68	33.91
3950	1.63	11.74	23.68	33.91
3949	1.63	11.73	23.68	33.91
3948	1.63	11.73	23.69	33.91
3947	1.63	11.74	23.69	33.91
3946	1.64	11.74	23.69	33.92
3945	1.64	11.74	23.69	33.93
3944	1.64	11.74	23.68	33.93
3943	1.64	11.74	23.68	33.93
3942	1.64	11.73	23.67	33.92
3941	1.65	11.73	23.67	33.92
3940	1.65	11.73	23.67	33.92
3939	1.65	11.73	23.67	33.92
3938	1.64	11.72	23.67	33.92
3937	1.64	11.72	23.67	33.91
3936	1.64	11.73	23.68	33.91
3935	1.64	11.74	23.68	33.91
3934	1.64	11.75	23.69	33.91
3933	1.63	11.76	23.69	33.92
3932	1.63	11.76	23.69	33.92
3931	1.62	11.76	23.69	33.92
3930	1.62	11.76	23.7	33.93
3929	1.62	11.76	23.71	33.93
3928	1.62	11.76	23.71	33.93
3927	1.62	11.75	23.71	33.93
3926	1.62	11.75	23.7	33.93
3925	1.62	11.74	23.7	33.94
3924	1.62	11.74	23.7	33.94
3923	1.62	11.75	23.7	33.93
3922	1.62	11.76	23.71	33.93
3921	1.62	11.76	23.71	33.92
3920	1.63	11.76	23.71	33.91
3919	1.63	11.76	23.71	33.91
3918	1.64	11.75	23.7	33.9
3917	1.64	11.75	23.69	33.9
3916	1.63	11.74	23.69	33.91
3915	1.63	11.74	23.69	33.91
3914	1.63	11.73	23.69	33.92
3913	1.63	11.73	23.69	33.92
3912	1.63	11.72	23.69	33.92
3911	1.62	11.71	23.69	33.91
3910	1.62	11.71	23.69	33.9
3909	1.62	11.72	23.69	33.9

3908	1.62	11.73	23.7	33.89
3907	1.62	11.75	23.71	33.89
3906	1.61	11.76	23.71	33.89
3905	1.61	11.77	23.71	33.89
3904	1.62	11.77	23.71	33.91
3903	1.62	11.78	23.71	33.92
3902	1.63	11.77	23.72	33.94
3901	1.63	11.77	23.71	33.95
3900	1.62	11.76	23.7	33.95
3899	1.61	11.75	23.69	33.94
3898	1.61	11.74	23.68	33.94
3897	1.61	11.73	23.68	33.93
3896	1.61	11.73	23.67	33.93
3895	1.62	11.73	23.67	33.92
3894	1.62	11.74	23.67	33.91
3893	1.63	11.74	23.67	33.91
3892	1.62	11.74	23.67	33.9
3891	1.62	11.74	23.68	33.89
3890	1.61	11.74	23.69	33.9
3889	1.61	11.75	23.69	33.9
3888	1.61	11.76	23.69	33.91
3887	1.6	11.76	23.67	33.91
3886	1.6	11.75	23.66	33.9
3885	1.6	11.75	23.66	33.91
3884	1.61	11.75	23.67	33.92
3883	1.62	11.76	23.68	33.93
3882	1.62	11.76	23.68	33.93
3881	1.61	11.75	23.67	33.92
3880	1.6	11.73	23.67	33.91
3879	1.6	11.73	23.67	33.91
3878	1.61	11.73	23.68	33.91
3877	1.62	11.73	23.68	33.92
3876	1.63	11.72	23.69	33.92
3875	1.62	11.71	23.69	33.92
3874	1.62	11.71	23.7	33.92
3873	1.62	11.72	23.71	33.92
3872	1.62	11.74	23.71	33.93
3871	1.61	11.75	23.71	33.93
3870	1.6	11.75	23.7	33.92
3869	1.6	11.75	23.7	33.91
3868	1.6	11.76	23.7	33.92
3867	1.61	11.76	23.7	33.92
3866	1.61	11.75	23.69	33.92
3865	1.61	11.73	23.68	33.91

3864	1.6	11.72	23.68	33.9
3863	1.59	11.72	23.69	33.91
3862	1.58	11.72	23.69	33.91
3861	1.57	11.72	23.68	33.9
3860	1.57	11.72	23.68	33.9
3859	1.56	11.72	23.68	33.9
3858	1.56	11.73	23.68	33.9
3857	1.56	11.75	23.69	33.91
3856	1.57	11.77	23.69	33.92
3855	1.57	11.77	23.68	33.91
3854	1.57	11.77	23.67	33.9
3853	1.58	11.77	23.68	33.9
3852	1.58	11.78	23.69	33.92
3851	1.59	11.78	23.68	33.92
3850	1.58	11.76	23.67	33.92
3849	1.58	11.74	23.66	33.91
3848	1.58	11.73	23.65	33.9
3847	1.58	11.72	23.65	33.89
3846	1.58	11.72	23.65	33.88
3845	1.57	11.72	23.65	33.88
3844	1.56	11.72	23.66	33.88
3843	1.56	11.73	23.67	33.88
3842	1.56	11.74	23.68	33.89
3841	1.57	11.74	23.7	33.89
3840	1.57	11.74	23.7	33.89
3839	1.57	11.73	23.71	33.89
3838	1.57	11.71	23.7	33.88
3837	1.57	11.7	23.69	33.89
3836	1.58	11.7	23.68	33.89
3835	1.58	11.7	23.67	33.9
3834	1.58	11.71	23.66	33.9
3833	1.58	11.71	23.65	33.9
3832	1.58	11.71	23.65	33.89
3831	1.58	11.71	23.64	33.88
3830	1.58	11.71	23.64	33.88
3829	1.59	11.71	23.64	33.87
3828	1.59	11.7	23.64	33.86
3827	1.58	11.7	23.64	33.85
3826	1.57	11.69	23.65	33.85
3825	1.57	11.69	23.66	33.85
3824	1.57	11.7	23.67	33.86
3823	1.58	11.7	23.67	33.87
3822	1.57	11.68	23.66	33.87
3821	1.56	11.67	23.67	33.87

3820	1.55	11.68	23.68	33.88
3819	1.56	11.7	23.69	33.9
3818	1.57	11.72	23.69	33.9
3817	1.56	11.72	23.68	33.89
3816	1.56	11.7	23.66	33.87
3815	1.55	11.69	23.65	33.86
3814	1.55	11.69	23.64	33.86
3813	1.56	11.68	23.64	33.86
3812	1.55	11.67	23.63	33.85
3811	1.55	11.67	23.63	33.85
3810	1.54	11.67	23.63	33.86
3809	1.53	11.68	23.64	33.87
3808	1.52	11.68	23.64	33.87
3807	1.51	11.67	23.64	33.86
3806	1.51	11.67	23.65	33.86
3805	1.52	11.68	23.66	33.87
3804	1.53	11.7	23.66	33.88
3803	1.53	11.7	23.65	33.88
3802	1.52	11.7	23.65	33.88
3801	1.51	11.7	23.65	33.88
3800	1.52	11.7	23.65	33.88
3799	1.53	11.71	23.65	33.89
3798	1.53	11.71	23.63	33.88
3797	1.52	11.7	23.62	33.86
3796	1.5	11.68	23.61	33.84
3795	1.5	11.67	23.6	33.83
3794	1.5	11.66	23.6	33.83
3793	1.5	11.65	23.6	33.83
3792	1.51	11.65	23.6	33.83
3791	1.51	11.64	23.59	33.83
3790	1.52	11.63	23.58	33.82
3789	1.52	11.63	23.58	33.82
3788	1.52	11.64	23.59	33.82
3787	1.53	11.64	23.59	33.82
3786	1.53	11.63	23.6	33.82
3785	1.53	11.63	23.6	33.82
3784	1.52	11.62	23.61	33.82
3783	1.52	11.61	23.61	33.82
3782	1.52	11.61	23.61	33.82
3781	1.52	11.62	23.61	33.82
3780	1.51	11.62	23.61	33.82
3779	1.51	11.62	23.6	33.82
3778	1.51	11.62	23.6	33.83
3777	1.51	11.63	23.6	33.83

3776	1.51	11.62	23.6	33.83
3775	1.51	11.62	23.6	33.82
3774	1.5	11.62	23.6	33.82
3773	1.5	11.62	23.6	33.82
3772	1.5	11.62	23.59	33.82
3771	1.49	11.62	23.59	33.82
3770	1.49	11.61	23.59	33.82
3769	1.49	11.61	23.6	33.83
3768	1.5	11.62	23.6	33.84
3767	1.49	11.62	23.59	33.84
3766	1.49	11.62	23.58	33.82
3765	1.48	11.62	23.58	33.81
3764	1.49	11.62	23.58	33.8
3763	1.49	11.62	23.58	33.8
3762	1.5	11.62	23.59	33.8
3761	1.5	11.62	23.59	33.8
3760	1.5	11.63	23.59	33.8
3759	1.5	11.64	23.6	33.8
3758	1.5	11.65	23.61	33.8
3757	1.5	11.65	23.62	33.81
3756	1.5	11.66	23.63	33.81
3755	1.5	11.66	23.65	33.82
3754	1.49	11.65	23.66	33.82
3753	1.49	11.64	23.66	33.82
3752	1.49	11.62	23.66	33.82
3751	1.49	11.62	23.65	33.83
3750	1.48	11.63	23.65	33.83
3749	1.48	11.64	23.65	33.82
3748	1.47	11.66	23.65	33.82
3747	1.48	11.66	23.66	33.83
3746	1.48	11.65	23.66	33.82
3745	1.47	11.62	23.65	33.8
3744	1.47	11.6	23.64	33.78
3743	1.47	11.6	23.63	33.78
3742	1.47	11.61	23.62	33.78
3741	1.47	11.61	23.61	33.78
3740	1.47	11.61	23.6	33.78
3739	1.46	11.62	23.6	33.78
3738	1.47	11.63	23.59	33.78
3737	1.47	11.64	23.59	33.79
3736	1.48	11.64	23.59	33.79
3735	1.48	11.65	23.6	33.79
3734	1.48	11.66	23.61	33.8
3733	1.48	11.65	23.61	33.79

3732	1.47	11.63	23.6	33.78
3731	1.47	11.61	23.59	33.78
3730	1.46	11.6	23.58	33.77
3729	1.46	11.59	23.57	33.77
3728	1.46	11.59	23.56	33.77
3727	1.46	11.58	23.55	33.77
3726	1.46	11.58	23.55	33.76
3725	1.46	11.59	23.55	33.77
3724	1.47	11.59	23.56	33.78
3723	1.47	11.59	23.56	33.79
3722	1.47	11.59	23.56	33.79
3721	1.47	11.59	23.57	33.79
3720	1.47	11.58	23.56	33.78
3719	1.47	11.58	23.56	33.78
3718	1.46	11.57	23.55	33.77
3717	1.45	11.57	23.55	33.76
3716	1.44	11.57	23.55	33.76
3715	1.44	11.57	23.56	33.76
3714	1.44	11.57	23.56	33.75
3713	1.43	11.57	23.56	33.74
3712	1.42	11.56	23.55	33.74
3711	1.42	11.56	23.55	33.76
3710	1.43	11.56	23.54	33.77
3709	1.43	11.56	23.54	33.77
3708	1.43	11.55	23.54	33.76
3707	1.43	11.54	23.54	33.75
3706	1.42	11.53	23.54	33.73
3705	1.42	11.53	23.54	33.73
3704	1.41	11.53	23.54	33.72
3703	1.41	11.53	23.53	33.73
3702	1.4	11.53	23.52	33.74
3701	1.4	11.53	23.52	33.75
3700	1.41	11.54	23.52	33.75
3699	1.41	11.54	23.53	33.75
3698	1.42	11.53	23.52	33.74
3697	1.42	11.53	23.52	33.73
3696	1.42	11.52	23.51	33.72
3695	1.42	11.53	23.51	33.72
3694	1.41	11.54	23.51	33.73
3693	1.4	11.55	23.52	33.74
3692	1.39	11.57	23.53	33.74
3691	1.38	11.58	23.53	33.73
3690	1.39	11.59	23.52	33.73
3689	1.41	11.6	23.52	33.73

3688	1.43	11.58	23.52	33.73
3687	1.44	11.57	23.52	33.73
3686	1.44	11.55	23.52	33.73
3685	1.44	11.54	23.52	33.73
3684	1.44	11.53	23.53	33.73
3683	1.44	11.53	23.53	33.73
3682	1.44	11.53	23.53	33.73
3681	1.43	11.54	23.53	33.72
3680	1.42	11.55	23.54	33.73
3679	1.42	11.57	23.55	33.74
3678	1.42	11.59	23.56	33.76
3677	1.41	11.6	23.56	33.76
3676	1.41	11.6	23.57	33.76
3675	1.4	11.59	23.59	33.75
3674	1.41	11.6	23.6	33.75
3673	1.43	11.6	23.6	33.75
3672	1.43	11.61	23.59	33.74
3671	1.42	11.6	23.57	33.73
3670	1.4	11.58	23.56	33.73
3669	1.39	11.57	23.56	33.74
3668	1.4	11.57	23.55	33.75
3667	1.4	11.55	23.54	33.74
3666	1.41	11.54	23.53	33.73
3665	1.41	11.53	23.52	33.72
3664	1.42	11.52	23.51	33.71
3663	1.42	11.51	23.51	33.71
3662	1.41	11.5	23.5	33.71
3661	1.41	11.5	23.5	33.71
3660	1.4	11.49	23.51	33.71
3659	1.39	11.5	23.51	33.71
3658	1.39	11.5	23.51	33.71
3657	1.39	11.51	23.51	33.7
3656	1.39	11.51	23.52	33.69
3655	1.4	11.52	23.53	33.68
3654	1.41	11.54	23.54	33.69
3653	1.43	11.56	23.55	33.7
3652	1.43	11.58	23.55	33.71
3651	1.42	11.57	23.55	33.71
3650	1.41	11.57	23.55	33.72
3649	1.41	11.57	23.56	33.75
3648	1.41	11.57	23.57	33.77
3647	1.42	11.57	23.57	33.77
3646	1.43	11.56	23.55	33.75
3645	1.43	11.55	23.54	33.73

3644	1.42	11.54	23.53	33.72
3643	1.42	11.52	23.52	33.7
3642	1.41	11.51	23.51	33.7
3641	1.41	11.5	23.51	33.69
3640	1.4	11.49	23.5	33.69
3639	1.4	11.49	23.5	33.68
3638	1.39	11.5	23.5	33.68
3637	1.38	11.5	23.5	33.68
3636	1.38	11.5	23.51	33.68
3635	1.37	11.5	23.51	33.68
3634	1.37	11.5	23.52	33.69
3633	1.37	11.5	23.54	33.69
3632	1.38	11.51	23.55	33.7
3631	1.4	11.52	23.56	33.71
3630	1.4	11.52	23.55	33.71
3629	1.39	11.53	23.54	33.72
3628	1.39	11.54	23.54	33.73
3627	1.39	11.55	23.53	33.74
3626	1.39	11.55	23.52	33.74
3625	1.4	11.54	23.51	33.73
3624	1.4	11.53	23.51	33.71
3623	1.41	11.52	23.51	33.7
3622	1.42	11.53	23.52	33.71
3621	1.43	11.53	23.53	33.72
3620	1.43	11.54	23.53	33.73
3619	1.43	11.54	23.52	33.74
3618	1.43	11.54	23.52	33.74
3617	1.43	11.53	23.52	33.74
3616	1.44	11.52	23.52	33.73
3615	1.44	11.52	23.51	33.73
3614	1.43	11.52	23.51	33.72
3613	1.42	11.53	23.52	33.72
3612	1.42	11.54	23.52	33.73
3611	1.42	11.55	23.53	33.73
3610	1.43	11.56	23.53	33.72
3609	1.43	11.56	23.52	33.71
3608	1.43	11.56	23.52	33.7
3607	1.43	11.55	23.52	33.7
3606	1.42	11.54	23.51	33.7
3605	1.41	11.53	23.51	33.7
3604	1.4	11.52	23.5	33.69
3603	1.4	11.51	23.5	33.69
3602	1.39	11.51	23.5	33.68
3601	1.39	11.5	23.51	33.67

3600	1.39	11.5	23.51	33.68
3599	1.38	11.49	23.51	33.68
3598	1.38	11.49	23.51	33.69
3597	1.39	11.48	23.51	33.69
3596	1.39	11.48	23.51	33.69
3595	1.39	11.49	23.51	33.69
3594	1.39	11.49	23.5	33.69
3593	1.38	11.49	23.5	33.69
3592	1.38	11.5	23.5	33.69
3591	1.37	11.5	23.5	33.69
3590	1.37	11.51	23.5	33.69
3589	1.36	11.51	23.5	33.68
3588	1.34	11.5	23.5	33.68
3587	1.34	11.49	23.49	33.68
3586	1.33	11.49	23.49	33.69
3585	1.34	11.49	23.48	33.68
3584	1.35	11.48	23.48	33.67
3583	1.35	11.48	23.47	33.66
3582	1.35	11.47	23.47	33.66
3581	1.35	11.47	23.47	33.65
3580	1.35	11.47	23.47	33.66
3579	1.34	11.47	23.48	33.66
3578	1.34	11.48	23.48	33.67
3577	1.33	11.48	23.48	33.67
3576	1.33	11.48	23.47	33.67
3575	1.34	11.48	23.46	33.67
3574	1.34	11.48	23.46	33.67
3573	1.35	11.47	23.46	33.68
3572	1.35	11.48	23.47	33.68
3571	1.35	11.49	23.48	33.69
3570	1.35	11.51	23.5	33.69
3569	1.34	11.52	23.5	33.7
3568	1.34	11.52	23.51	33.7
3567	1.34	11.52	23.51	33.72
3566	1.35	11.52	23.51	33.73
3565	1.36	11.52	23.51	33.73
3564	1.36	11.51	23.51	33.72
3563	1.37	11.51	23.51	33.71
3562	1.38	11.5	23.5	33.69
3561	1.38	11.5	23.5	33.68
3560	1.39	11.51	23.5	33.68
3559	1.39	11.51	23.5	33.67
3558	1.39	11.52	23.49	33.68
3557	1.38	11.53	23.49	33.68

3556	1.38	11.53	23.49	33.68
3555	1.38	11.53	23.49	33.69
3554	1.39	11.53	23.49	33.69
3553	1.39	11.53	23.5	33.7
3552	1.4	11.52	23.5	33.71
3551	1.4	11.52	23.51	33.72
3550	1.41	11.53	23.51	33.72
3549	1.41	11.54	23.51	33.72
3548	1.42	11.54	23.51	33.72
3547	1.42	11.55	23.5	33.71
3546	1.42	11.55	23.51	33.71
3545	1.43	11.56	23.51	33.71
3544	1.43	11.56	23.52	33.72
3543	1.43	11.55	23.53	33.73
3542	1.42	11.55	23.53	33.73
3541	1.42	11.55	23.53	33.73
3540	1.41	11.55	23.53	33.72
3539	1.42	11.55	23.52	33.72
3538	1.42	11.55	23.52	33.72
3537	1.42	11.55	23.51	33.72
3536	1.42	11.54	23.51	33.72
3535	1.42	11.54	23.51	33.71
3534	1.42	11.54	23.51	33.71
3533	1.42	11.54	23.51	33.7
3532	1.42	11.54	23.51	33.7
3531	1.43	11.54	23.51	33.69
3530	1.43	11.53	23.51	33.69
3529	1.44	11.53	23.51	33.69
3528	1.44	11.53	23.52	33.7
3527	1.44	11.54	23.52	33.71
3526	1.44	11.55	23.52	33.71
3525	1.44	11.57	23.52	33.72
3524	1.44	11.57	23.53	33.73
3523	1.44	11.57	23.53	33.73
3522	1.44	11.56	23.53	33.73
3521	1.44	11.55	23.53	33.73
3520	1.43	11.54	23.52	33.72
3519	1.43	11.53	23.52	33.72
3518	1.43	11.53	23.52	33.71
3517	1.43	11.53	23.52	33.71
3516	1.43	11.54	23.52	33.71
3515	1.42	11.54	23.52	33.71
3514	1.42	11.54	23.52	33.71
3513	1.41	11.54	23.52	33.72

3512	1.42	11.54	23.53	33.72
3511	1.42	11.55	23.53	33.73
3510	1.43	11.55	23.54	33.73
3509	1.43	11.56	23.54	33.73
3508	1.43	11.56	23.55	33.73
3507	1.43	11.56	23.55	33.73
3506	1.43	11.56	23.54	33.73
3505	1.43	11.56	23.54	33.74
3504	1.43	11.56	23.54	33.74
3503	1.44	11.57	23.54	33.74
3502	1.44	11.57	23.54	33.75
3501	1.46	11.57	23.55	33.75
3500	1.47	11.57	23.55	33.75
3499	1.48	11.57	23.55	33.75
3498	1.49	11.58	23.56	33.75
3497	1.5	11.58	23.56	33.75
3496	1.5	11.59	23.56	33.75
3495	1.5	11.6	23.56	33.75
3494	1.5	11.6	23.56	33.75
3493	1.51	11.61	23.57	33.75
3492	1.52	11.62	23.58	33.75
3491	1.53	11.63	23.58	33.76
3490	1.54	11.65	23.59	33.78
3489	1.56	11.65	23.6	33.79
3488	1.57	11.66	23.61	33.81
3487	1.58	11.67	23.62	33.82
3486	1.6	11.69	23.63	33.83
3485	1.61	11.7	23.64	33.84
3484	1.62	11.71	23.64	33.85
3483	1.64	11.72	23.65	33.85
3482	1.65	11.73	23.66	33.86
3481	1.65	11.74	23.67	33.87
3480	1.66	11.74	23.68	33.88
3479	1.66	11.75	23.68	33.88
3478	1.66	11.75	23.69	33.89
3477	1.67	11.75	23.69	33.89
3476	1.67	11.75	23.7	33.89
3475	1.67	11.75	23.7	33.89
3474	1.67	11.76	23.71	33.89
3473	1.68	11.76	23.7	33.88
3472	1.68	11.77	23.7	33.88
3471	1.67	11.76	23.69	33.87
3470	1.67	11.75	23.69	33.87
3469	1.67	11.74	23.69	33.88

3468	1.66	11.74	23.69	33.88
3467	1.66	11.74	23.7	33.89
3466	1.66	11.75	23.7	33.89
3465	1.66	11.75	23.7	33.88
3464	1.66	11.76	23.7	33.88
3463	1.66	11.76	23.7	33.88
3462	1.66	11.76	23.7	33.88
3461	1.66	11.77	23.7	33.88
3460	1.67	11.77	23.7	33.89
3459	1.67	11.77	23.7	33.9
3458	1.68	11.77	23.7	33.9
3457	1.69	11.77	23.7	33.9
3456	1.69	11.77	23.71	33.89
3455	1.7	11.77	23.71	33.89
3454	1.7	11.77	23.71	33.9
3453	1.69	11.77	23.71	33.9
3452	1.69	11.77	23.71	33.91
3451	1.68	11.78	23.71	33.92
3450	1.69	11.79	23.71	33.92
3449	1.69	11.79	23.72	33.92
3448	1.7	11.8	23.72	33.92
3447	1.7	11.81	23.72	33.92
3446	1.71	11.81	23.72	33.91
3445	1.71	11.81	23.72	33.92
3444	1.72	11.81	23.72	33.92
3443	1.72	11.81	23.73	33.93
3442	1.71	11.8	23.74	33.93
3441	1.71	11.8	23.74	33.93
3440	1.7	11.8	23.74	33.93
3439	1.7	11.8	23.74	33.93
3438	1.7	11.8	23.74	33.93
3437	1.7	11.8	23.74	33.93
3436	1.71	11.81	23.74	33.94
3435	1.71	11.81	23.74	33.94
3434	1.71	11.81	23.74	33.94
3433	1.72	11.81	23.74	33.94
3432	1.72	11.81	23.74	33.94
3431	1.72	11.81	23.75	33.93
3430	1.72	11.81	23.75	33.94
3429	1.72	11.81	23.75	33.94
3428	1.73	11.81	23.75	33.95
3427	1.73	11.81	23.75	33.95
3426	1.74	11.82	23.76	33.95
3425	1.74	11.82	23.76	33.95

3424	1.74	11.82	23.76	33.95
3423	1.74	11.82	23.76	33.95
3422	1.73	11.82	23.76	33.95
3421	1.72	11.81	23.76	33.95
3420	1.71	11.81	23.75	33.94
3419	1.7	11.81	23.75	33.94
3418	1.69	11.81	23.74	33.93
3417	1.69	11.81	23.74	33.93
3416	1.69	11.81	23.73	33.93
3415	1.69	11.81	23.72	33.93
3414	1.69	11.81	23.72	33.93
3413	1.7	11.81	23.72	33.93
3412	1.7	11.81	23.73	33.93
3411	1.7	11.81	23.73	33.93
3410	1.69	11.81	23.73	33.93
3409	1.69	11.81	23.73	33.93
3408	1.68	11.81	23.72	33.93
3407	1.68	11.81	23.72	33.93
3406	1.68	11.8	23.72	33.93
3405	1.68	11.79	23.71	33.93
3404	1.68	11.78	23.71	33.93
3403	1.68	11.78	23.71	33.94
3402	1.68	11.78	23.71	33.94
3401	1.68	11.78	23.71	33.93
3400	1.68	11.79	23.71	33.93
3399	1.67	11.79	23.71	33.93
3398	1.67	11.8	23.71	33.93
3397	1.67	11.81	23.71	33.93
3396	1.67	11.81	23.71	33.93
3395	1.67	11.81	23.71	33.92
3394	1.67	11.8	23.72	33.92
3393	1.67	11.79	23.72	33.92
3392	1.67	11.79	23.73	33.93
3391	1.67	11.79	23.73	33.93
3390	1.67	11.79	23.73	33.93
3389	1.67	11.79	23.73	33.93
3388	1.67	11.79	23.73	33.93
3387	1.67	11.8	23.74	33.93
3386	1.67	11.8	23.74	33.93
3385	1.68	11.8	23.74	33.93
3384	1.68	11.8	23.74	33.93
3383	1.68	11.8	23.74	33.93
3382	1.67	11.8	23.74	33.93
3381	1.67	11.8	23.73	33.94

3380	1.66	11.81	23.73	33.94
3379	1.66	11.81	23.72	33.95
3378	1.66	11.81	23.73	33.95
3377	1.67	11.81	23.73	33.96
3376	1.67	11.82	23.73	33.95
3375	1.68	11.82	23.73	33.95
3374	1.69	11.82	23.74	33.94
3373	1.69	11.82	23.75	33.94
3372	1.69	11.82	23.76	33.93
3371	1.69	11.83	23.77	33.93
3370	1.68	11.83	23.77	33.93
3369	1.68	11.83	23.77	33.93
3368	1.68	11.82	23.77	33.94
3367	1.68	11.83	23.76	33.94
3366	1.68	11.83	23.76	33.95
3365	1.68	11.83	23.75	33.95
3364	1.68	11.84	23.75	33.96
3363	1.68	11.84	23.75	33.96
3362	1.69	11.85	23.75	33.96
3361	1.69	11.85	23.76	33.97
3360	1.7	11.85	23.76	33.97
3359	1.7	11.86	23.77	33.98
3358	1.7	11.86	23.77	33.98
3357	1.7	11.87	23.77	33.98
3356	1.71	11.87	23.77	33.98
3355	1.71	11.87	23.77	33.98
3354	1.71	11.87	23.78	33.99
3353	1.71	11.87	23.78	33.99
3352	1.71	11.87	23.78	33.99
3351	1.71	11.87	23.77	33.98
3350	1.71	11.87	23.77	33.97
3349	1.71	11.88	23.78	33.97
3348	1.71	11.88	23.78	33.97
3347	1.71	11.88	23.79	33.98
3346	1.71	11.88	23.79	33.98
3345	1.72	11.88	23.79	33.98
3344	1.72	11.88	23.79	33.98
3343	1.72	11.88	23.79	33.99
3342	1.72	11.87	23.79	34
3341	1.72	11.87	23.79	34.01
3340	1.72	11.87	23.79	34.01
3339	1.72	11.87	23.79	34.01
3338	1.72	11.87	23.8	34
3337	1.72	11.88	23.8	33.99

3336	1.73	11.88	23.8	33.99
3335	1.74	11.89	23.8	33.99
3334	1.74	11.89	23.8	33.99
3333	1.75	11.89	23.8	33.99
3332	1.75	11.88	23.81	34
3331	1.75	11.88	23.81	34.01
3330	1.74	11.87	23.82	34.01
3329	1.73	11.87	23.82	34.01
3328	1.73	11.87	23.83	34.01
3327	1.73	11.88	23.83	34.01
3326	1.73	11.88	23.83	34.01
3325	1.72	11.89	23.83	34.01
3324	1.73	11.9	23.83	34.01
3323	1.73	11.91	23.84	34.02
3322	1.73	11.91	23.84	34.01
3321	1.74	11.91	23.83	34.01
3320	1.75	11.91	23.83	34.01
3319	1.75	11.91	23.82	34.01
3318	1.75	11.91	23.82	34.01
3317	1.74	11.91	23.82	34.01
3316	1.74	11.92	23.81	34.01
3315	1.74	11.92	23.81	34.02
3314	1.73	11.92	23.81	34.03
3313	1.72	11.91	23.81	34.03
3312	1.72	11.91	23.81	34.02
3311	1.73	11.9	23.8	34.01
3310	1.73	11.9	23.8	34
3309	1.74	11.9	23.8	34
3308	1.74	11.9	23.81	34
3307	1.74	11.9	23.81	34
3306	1.73	11.91	23.82	34.01
3305	1.73	11.91	23.82	34.02
3304	1.73	11.91	23.81	34.02
3303	1.73	11.9	23.81	34.03
3302	1.74	11.9	23.81	34.03
3301	1.75	11.91	23.82	34.03
3300	1.75	11.91	23.82	34.03
3299	1.76	11.92	23.82	34.03
3298	1.75	11.93	23.82	34.02
3297	1.75	11.93	23.82	34.02
3296	1.73	11.93	23.82	34.01
3295	1.73	11.92	23.82	34.01
3294	1.72	11.91	23.82	34.01
3293	1.72	11.9	23.82	34.01

3292	1.72	11.9	23.82	34.01
3291	1.72	11.9	23.81	34.01
3290	1.73	11.91	23.81	34.01
3289	1.74	11.91	23.81	34.01
3288	1.75	11.91	23.81	34.01
3287	1.75	11.92	23.81	34.01
3286	1.75	11.92	23.82	34.01
3285	1.75	11.92	23.82	34.01
3284	1.75	11.92	23.82	34
3283	1.75	11.92	23.82	34
3282	1.75	11.93	23.83	34.01
3281	1.74	11.94	23.82	34.01
3280	1.74	11.94	23.82	34.02
3279	1.75	11.94	23.82	34.02
3278	1.75	11.93	23.82	34.02
3277	1.74	11.93	23.82	34.01
3276	1.74	11.92	23.82	34.01
3275	1.72	11.92	23.81	34.01
3274	1.71	11.91	23.81	34.01
3273	1.71	11.91	23.8	34
3272	1.71	11.91	23.81	34
3271	1.72	11.91	23.81	34
3270	1.72	11.91	23.81	34
3269	1.72	11.91	23.82	34
3268	1.72	11.91	23.82	33.99
3267	1.72	11.91	23.82	33.99
3266	1.71	11.91	23.82	33.99
3265	1.7	11.91	23.82	33.99
3264	1.69	11.9	23.82	33.99
3263	1.69	11.9	23.81	33.99
3262	1.69	11.89	23.81	33.99
3261	1.69	11.89	23.81	33.99
3260	1.69	11.89	23.8	33.99
3259	1.7	11.89	23.79	33.99
3258	1.7	11.88	23.79	33.98
3257	1.7	11.88	23.79	33.97
3256	1.69	11.88	23.79	33.97
3255	1.69	11.89	23.79	33.96
3254	1.68	11.89	23.79	33.96
3253	1.67	11.9	23.79	33.96
3252	1.67	11.89	23.79	33.96
3251	1.67	11.89	23.79	33.96
3250	1.67	11.89	23.79	33.96
3249	1.67	11.88	23.78	33.96

3248	1.68	11.88	23.77	33.96
3247	1.67	11.87	23.76	33.96
3246	1.68	11.86	23.76	33.96
3245	1.68	11.86	23.76	33.96
3244	1.69	11.86	23.76	33.97
3243	1.7	11.87	23.76	33.97
3242	1.7	11.87	23.75	33.97
3241	1.7	11.87	23.75	33.97
3240	1.69	11.87	23.75	33.97
3239	1.69	11.86	23.75	33.97
3238	1.69	11.86	23.75	33.97
3237	1.68	11.85	23.75	33.96
3236	1.68	11.85	23.75	33.95
3235	1.68	11.84	23.75	33.95
3234	1.67	11.84	23.75	33.94
3233	1.67	11.84	23.74	33.94
3232	1.67	11.85	23.74	33.95
3231	1.67	11.85	23.74	33.95
3230	1.67	11.85	23.74	33.95
3229	1.67	11.85	23.75	33.95
3228	1.68	11.86	23.75	33.95
3227	1.68	11.86	23.75	33.94
3226	1.68	11.87	23.75	33.94
3225	1.68	11.87	23.76	33.94
3224	1.68	11.86	23.76	33.94
3223	1.68	11.87	23.76	33.94
3222	1.67	11.87	23.76	33.94
3221	1.67	11.87	23.76	33.94
3220	1.67	11.87	23.75	33.94
3219	1.67	11.86	23.75	33.93
3218	1.67	11.85	23.75	33.93
3217	1.68	11.85	23.75	33.93
3216	1.68	11.85	23.76	33.93
3215	1.68	11.85	23.77	33.92
3214	1.68	11.86	23.77	33.92
3213	1.68	11.87	23.77	33.92
3212	1.69	11.87	23.77	33.92
3211	1.69	11.87	23.76	33.92
3210	1.7	11.87	23.76	33.92
3209	1.7	11.87	23.75	33.93
3208	1.69	11.86	23.75	33.93
3207	1.69	11.86	23.75	33.93
3206	1.69	11.86	23.76	33.92
3205	1.7	11.87	23.76	33.93

3204	1.7	11.87	23.75	33.93
3203	1.71	11.87	23.75	33.94
3202	1.71	11.87	23.74	33.94
3201	1.71	11.87	23.73	33.94
3200	1.71	11.87	23.73	33.93
3199	1.71	11.87	23.73	33.93
3198	1.7	11.87	23.74	33.94
3197	1.7	11.87	23.74	33.94
3196	1.71	11.87	23.75	33.94
3195	1.71	11.88	23.75	33.93
3194	1.71	11.88	23.75	33.93
3193	1.72	11.88	23.75	33.93
3192	1.72	11.88	23.75	33.94
3191	1.72	11.88	23.75	33.94
3190	1.72	11.88	23.74	33.94
3189	1.72	11.88	23.74	33.93
3188	1.71	11.88	23.74	33.91
3187	1.71	11.87	23.74	33.9
3186	1.71	11.87	23.74	33.9
3185	1.71	11.87	23.74	33.9
3184	1.71	11.87	23.74	33.91
3183	1.72	11.86	23.73	33.92
3182	1.72	11.86	23.72	33.92
3181	1.73	11.86	23.72	33.92
3180	1.72	11.86	23.72	33.91
3179	1.72	11.86	23.72	33.91
3178	1.71	11.86	23.72	33.9
3177	1.71	11.85	23.73	33.9
3176	1.7	11.85	23.73	33.9
3175	1.69	11.84	23.73	33.9
3174	1.69	11.84	23.72	33.89
3173	1.68	11.84	23.72	33.89
3172	1.68	11.84	23.71	33.89
3171	1.68	11.84	23.7	33.89
3170	1.68	11.84	23.69	33.89
3169	1.69	11.83	23.69	33.89
3168	1.69	11.82	23.69	33.88
3167	1.69	11.81	23.69	33.87
3166	1.68	11.81	23.69	33.86
3165	1.68	11.81	23.69	33.85
3164	1.68	11.81	23.69	33.84
3163	1.68	11.81	23.69	33.84
3162	1.67	11.81	23.69	33.84
3161	1.67	11.81	23.69	33.84

3160	1.66	11.8	23.68	33.84
3159	1.66	11.79	23.68	33.83
3158	1.66	11.79	23.67	33.82
3157	1.66	11.78	23.66	33.82
3156	1.65	11.78	23.65	33.82
3155	1.64	11.77	23.64	33.81
3154	1.63	11.76	23.64	33.8
3153	1.63	11.75	23.63	33.8
3152	1.63	11.75	23.63	33.8
3151	1.62	11.75	23.63	33.79
3150	1.62	11.75	23.63	33.79
3149	1.63	11.75	23.63	33.79
3148	1.63	11.75	23.62	33.79
3147	1.63	11.75	23.62	33.79
3146	1.63	11.75	23.61	33.78
3145	1.62	11.74	23.61	33.78
3144	1.61	11.74	23.6	33.77
3143	1.6	11.73	23.6	33.76
3142	1.59	11.73	23.6	33.75
3141	1.58	11.72	23.59	33.75
3140	1.59	11.72	23.58	33.74
3139	1.59	11.72	23.58	33.74
3138	1.6	11.71	23.57	33.74
3137	1.6	11.71	23.57	33.74
3136	1.6	11.72	23.57	33.74
3135	1.6	11.72	23.56	33.74
3134	1.59	11.72	23.56	33.74
3133	1.59	11.71	23.55	33.74
3132	1.59	11.71	23.54	33.73
3131	1.59	11.71	23.53	33.73
3130	1.58	11.7	23.53	33.73
3129	1.58	11.7	23.53	33.73
3128	1.58	11.7	23.54	33.72
3127	1.58	11.71	23.54	33.72
3126	1.58	11.71	23.55	33.72
3125	1.58	11.7	23.54	33.71
3124	1.58	11.7	23.54	33.71
3123	1.58	11.69	23.53	33.7
3122	1.59	11.69	23.53	33.69
3121	1.59	11.69	23.53	33.69
3120	1.59	11.69	23.53	33.69
3119	1.59	11.69	23.53	33.68
3118	1.59	11.69	23.54	33.68
3117	1.6	11.7	23.53	33.68

3116	1.6	11.7	23.53	33.69
3115	1.6	11.7	23.53	33.69
3114	1.6	11.71	23.52	33.7
3113	1.6	11.71	23.53	33.7
3112	1.61	11.71	23.53	33.71
3111	1.61	11.72	23.53	33.71
3110	1.61	11.72	23.54	33.71
3109	1.62	11.73	23.54	33.71
3108	1.63	11.73	23.54	33.72
3107	1.64	11.73	23.54	33.72
3106	1.65	11.73	23.55	33.73
3105	1.66	11.73	23.55	33.73
3104	1.67	11.74	23.56	33.74
3103	1.67	11.75	23.56	33.74
3102	1.68	11.76	23.57	33.74
3101	1.68	11.77	23.58	33.74
3100	1.68	11.78	23.58	33.75
3099	1.68	11.78	23.59	33.75
3098	1.69	11.79	23.6	33.76
3097	1.7	11.8	23.6	33.76
3096	1.71	11.81	23.6	33.76
3095	1.73	11.82	23.61	33.77
3094	1.74	11.82	23.62	33.78
3093	1.75	11.83	23.63	33.8
3092	1.76	11.85	23.64	33.81
3091	1.77	11.86	23.65	33.82
3090	1.78	11.88	23.66	33.83
3089	1.79	11.91	23.68	33.84
3088	1.82	11.93	23.69	33.86
3087	1.84	11.97	23.71	33.88
3086	1.87	12	23.73	33.91
3085	1.9	12.03	23.75	33.94
3084	1.93	12.06	23.77	33.97
3083	1.96	12.09	23.79	33.99
3082	1.99	12.11	23.81	34.01
3081	2.01	12.13	23.83	34.03
3080	2.04	12.15	23.86	34.05
3079	2.07	12.17	23.88	34.07
3078	2.1	12.19	23.91	34.1
3077	2.13	12.21	23.93	34.12
3076	2.16	12.24	23.96	34.14
3075	2.19	12.27	23.98	34.17
3074	2.22	12.3	24.01	34.19
3073	2.25	12.33	24.03	34.22

3072	2.28	12.36	24.06	34.24
3071	2.31	12.39	24.08	34.27
3070	2.35	12.42	24.11	34.3
3069	2.38	12.45	24.13	34.33
3068	2.41	12.49	24.16	34.35
3067	2.43	12.53	24.19	34.38
3066	2.46	12.56	24.22	34.41
3065	2.48	12.6	24.26	34.44
3064	2.51	12.63	24.29	34.47
3063	2.55	12.66	24.32	34.5
3062	2.58	12.68	24.35	34.53
3061	2.61	12.7	24.37	34.55
3060	2.64	12.72	24.39	34.58
3059	2.67	12.73	24.41	34.59
3058	2.69	12.75	24.43	34.61
3057	2.71	12.76	24.44	34.62
3056	2.72	12.78	24.45	34.63
3055	2.73	12.78	24.45	34.64
3054	2.74	12.78	24.45	34.65
3053	2.75	12.79	24.45	34.66
3052	2.75	12.79	24.45	34.66
3051	2.75	12.8	24.46	34.66
3050	2.76	12.8	24.46	34.66
3049	2.76	12.8	24.47	34.66
3048	2.77	12.8	24.48	34.66
3047	2.77	12.8	24.49	34.66
3046	2.77	12.8	24.49	34.66
3045	2.78	12.8	24.48	34.66
3044	2.78	12.81	24.48	34.66
3043	2.77	12.81	24.48	34.67
3042	2.77	12.82	24.48	34.67
3041	2.78	12.83	24.49	34.68
3040	2.79	12.84	24.5	34.69
3039	2.81	12.86	24.52	34.7
3038	2.82	12.87	24.53	34.71
3037	2.84	12.89	24.55	34.72
3036	2.86	12.91	24.56	34.74
3035	2.87	12.93	24.57	34.75
3034	2.9	12.96	24.59	34.77
3033	2.92	13	24.61	34.79
3032	2.96	13.04	24.63	34.82
3031	2.99	13.08	24.66	34.85
3030	3.03	13.12	24.68	34.88
3029	3.06	13.17	24.7	34.91

3028	3.08	13.2	24.72	34.93
3027	3.11	13.23	24.74	34.95
3026	3.14	13.25	24.76	34.97
3025	3.16	13.27	24.79	34.98
3024	3.18	13.28	24.8	35
3023	3.2	13.29	24.81	35.01
3022	3.2	13.31	24.82	35.02
3021	3.21	13.32	24.83	35.03
3020	3.22	13.34	24.84	35.04
3019	3.24	13.35	24.85	35.05
3018	3.26	13.37	24.87	35.07
3017	3.29	13.39	24.89	35.09
3016	3.32	13.42	24.9	35.11
3015	3.35	13.45	24.92	35.13
3014	3.38	13.48	24.94	35.16
3013	3.41	13.52	24.97	35.18
3012	3.44	13.55	25	35.21
3011	3.46	13.58	25.02	35.23
3010	3.49	13.61	25.04	35.25
3009	3.52	13.63	25.06	35.27
3008	3.55	13.66	25.08	35.29
3007	3.57	13.68	25.1	35.31
3006	3.6	13.7	25.11	35.33
3005	3.62	13.72	25.13	35.35
3004	3.64	13.74	25.15	35.36
3003	3.66	13.76	25.16	35.38
3002	3.68	13.78	25.18	35.4
3001	3.7	13.8	25.2	35.43
3000	3.73	13.83	25.22	35.45
2999	3.77	13.86	25.25	35.48
2998	3.81	13.9	25.28	35.51
2997	3.86	13.94	25.32	35.55
2996	3.92	13.99	25.37	35.6
2995	3.99	14.06	25.43	35.66
2994	4.07	14.13	25.5	35.74
2993	4.16	14.23	25.58	35.83
2992	4.27	14.33	25.68	35.94
2991	4.39	14.46	25.8	36.06
2990	4.52	14.6	25.93	36.2
2989	4.68	14.76	26.07	36.35
2988	4.86	14.93	26.23	36.52
2987	5.06	15.13	26.41	36.7
2986	5.29	15.34	26.61	36.91
2985	5.54	15.57	26.84	37.15

2984	5.81	15.83	27.1	37.41
2983	6.11	16.12	27.37	37.7
2982	6.44	16.45	27.67	38.02
2981	6.79	16.8	28	38.37
2980	7.18	17.2	28.37	38.75
2979	7.61	17.62	28.76	39.17
2978	8.07	18.08	29.2	39.63
2977	8.58	18.58	29.67	40.11
2976	9.12	19.12	30.18	40.64
2975	9.71	19.7	30.74	41.22
2974	10.34	20.32	31.33	41.84
2973	11.02	20.99	31.98	42.5
2972	11.73	21.69	32.65	43.21
2971	12.48	22.43	33.37	43.95
2970	13.26	23.21	34.13	44.73
2969	14.08	24.01	34.92	45.54
2968	14.92	24.85	35.73	46.38
2967	15.78	25.7	36.57	47.24
2966	16.65	26.56	37.41	48.1
2965	17.52	27.41	38.25	48.95
2964	18.37	28.26	39.08	49.8
2963	19.19	29.08	39.88	50.62
2962	19.98	29.86	40.65	51.4
2961	20.73	30.59	41.37	52.13
2960	21.43	31.28	42.05	52.82
2959	22.08	31.91	42.67	53.46
2958	22.67	32.49	43.24	54.04
2957	23.2	33.01	43.75	54.56
2956	23.66	33.46	44.18	55
2955	24.04	33.83	44.53	55.36
2954	24.33	34.11	44.81	55.64
2953	24.53	34.31	44.99	55.83
2952	24.64	34.42	45.1	55.93
2951	24.67	34.45	45.14	55.96
2950	24.64	34.44	45.13	55.95
2949	24.6	34.39	45.08	55.9
2948	24.55	34.34	45.03	55.86
2947	24.52	34.32	44.99	55.83
2946	24.53	34.35	45	55.85
2945	24.62	34.45	45.08	55.94
2944	24.79	34.63	45.25	56.11
2943	25.07	34.91	45.51	56.38
2942	25.45	35.3	45.87	56.77
2941	25.95	35.81	46.34	57.26

2940	26.56	36.43	46.92	57.87
2939	27.29	37.17	47.61	58.57
2938	28.12	38	48.4	59.38
2937	29.04	38.92	49.28	60.28
2936	30.04	39.91	50.23	61.25
2935	31.09	40.96	51.24	62.29
2934	32.19	42.05	52.29	63.36
2933	33.31	43.17	53.38	64.46
2932	34.44	44.3	54.47	65.57
2931	35.57	45.43	55.57	66.68
2930	36.68	46.54	56.65	67.77
2929	37.75	47.61	57.7	68.83
2928	38.76	48.63	58.69	69.83
2927	39.7	49.58	59.63	70.76
2926	40.56	50.44	60.48	71.62
2925	41.3	51.19	61.24	72.36
2924	41.91	51.82	61.87	72.98
2923	42.37	52.3	62.37	73.46
2922	42.68	52.64	62.72	73.8
2921	42.81	52.81	62.92	73.98
2920	42.78	52.82	62.95	74
2919	42.58	52.66	62.82	73.85
2918	42.21	52.32	62.51	73.52
2917	41.68	51.79	62.03	73
2916	40.98	51.08	61.37	72.32
2915	40.15	50.22	60.58	71.5
2914	39.24	49.29	59.7	70.61
2913	38.32	48.33	58.81	69.71
2912	37.41	47.4	57.92	68.82
2911	36.56	46.52	57.09	67.98
2910	35.75	45.7	56.31	67.2
2909	34.99	44.94	55.58	66.47
2908	34.28	44.23	54.9	65.78
2907	33.61	43.56	54.26	65.13
2906	32.98	42.92	53.65	64.52
2905	32.38	42.32	53.08	63.94
2904	31.82	41.75	52.54	63.38
2903	31.27	41.2	52.02	62.85
2902	30.75	40.67	51.52	62.35
2901	30.24	40.16	51.03	61.85
2900	29.73	39.66	50.55	61.37
2899	29.24	39.17	50.08	60.89
2898	28.75	38.67	49.61	60.41
2897	28.26	38.18	49.15	59.94

2896	27.76	37.69	48.68	59.45
2895	27.26	37.19	48.21	58.97
2894	26.76	36.68	47.73	58.48
2893	26.25	36.16	47.24	57.98
2892	25.73	35.64	46.75	57.48
2891	25.21	35.12	46.26	56.97
2890	24.68	34.6	45.76	56.47
2889	24.16	34.08	45.27	55.96
2888	23.65	33.57	44.79	55.46
2887	23.16	33.08	44.31	54.98
2886	22.69	32.61	43.86	54.52
2885	22.25	32.18	43.44	54.1
2884	21.86	31.79	43.05	53.71
2883	21.52	31.45	42.72	53.38
2882	21.24	31.17	42.45	53.11
2881	21.04	30.97	42.25	52.91
2880	20.93	30.86	42.13	52.79
2879	20.91	30.84	42.1	52.76
2878	21	30.92	42.17	52.83
2877	21.19	31.1	42.33	52.99
2876	21.47	31.37	42.58	53.23
2875	21.83	31.72	42.9	53.56
2874	22.23	32.12	43.28	53.94
2873	22.66	32.54	43.68	54.34
2872	23.07	32.95	44.06	54.74
2871	23.44	33.32	44.42	55.1
2870	23.75	33.63	44.72	55.41
2869	24.01	33.87	44.96	55.65
2868	24.21	34.08	45.16	55.86
2867	24.39	34.26	45.34	56.04
2866	24.58	34.44	45.52	56.22
2865	24.79	34.66	45.73	56.43
2864	25.07	34.93	45.99	56.7
2863	25.42	35.28	46.32	57.03
2862	25.85	35.72	46.74	57.46
2861	26.38	36.26	47.25	57.98
2860	27	36.89	47.84	58.59
2859	27.69	37.58	48.5	59.27
2858	28.43	38.32	49.21	59.99
2857	29.17	39.06	49.94	60.73
2856	29.89	39.78	50.65	61.45
2855	30.53	40.44	51.3	62.1
2854	31.06	41	51.85	62.65
2853	31.44	41.41	52.28	63.07

2852	31.63	41.65	52.54	63.32
2851	31.61	41.67	52.58	63.36
2850	31.32	41.42	52.38	63.14
2849	30.76	40.88	51.89	62.63
2848	29.94	40.06	51.12	61.85
2847	28.92	39.02	50.15	60.85
2846	27.77	37.85	49.05	59.73
2845	26.58	36.63	47.9	58.57
2844	25.38	35.42	46.75	57.4
2843	24.2	34.24	45.63	56.25
2842	23.05	33.08	44.53	55.14
2841	21.92	31.96	43.47	54.06
2840	20.83	30.88	42.43	53
2839	19.77	29.82	41.43	51.98
2838	18.75	28.81	40.46	51
2837	17.78	27.85	39.54	50.06
2836	16.87	26.94	38.68	49.18
2835	16.02	26.09	37.87	48.35
2834	15.23	25.3	37.11	47.58
2833	14.5	24.57	36.42	46.88
2832	13.83	23.9	35.79	46.23
2831	13.21	23.29	35.21	45.63
2830	12.64	22.73	34.68	45.08
2829	12.12	22.22	34.19	44.59
2828	11.65	21.76	33.74	44.13
2827	11.22	21.33	33.33	43.72
2826	10.83	20.93	32.95	43.33
2825	10.47	20.57	32.61	42.98
2824	10.13	20.24	32.29	42.66
2823	9.83	19.93	31.99	42.36
2822	9.54	19.65	31.72	42.08
2821	9.27	19.39	31.47	41.83
2820	9.03	19.14	31.24	41.59
2819	8.79	18.91	31.02	41.36
2818	8.58	18.7	30.82	41.15
2817	8.37	18.5	30.63	40.95
2816	8.18	18.31	30.44	40.76
2815	8.01	18.14	30.27	40.59
2814	7.84	17.97	30.11	40.43
2813	7.68	17.81	29.96	40.28
2812	7.53	17.66	29.82	40.13
2811	7.39	17.51	29.68	39.99
2810	7.25	17.38	29.56	39.87
2809	7.12	17.25	29.44	39.75

2808	7	17.14	29.32	39.63
2807	6.88	17.02	29.21	39.51
2806	6.77	16.92	29.1	39.4
2805	6.66	16.82	29	39.29
2804	6.56	16.72	28.9	39.19
2803	6.46	16.62	28.81	39.09
2802	6.36	16.52	28.71	39
2801	6.27	16.43	28.62	38.91
2800	6.17	16.33	28.53	38.82
2799	6.09	16.24	28.44	38.73
2798	6	16.16	28.36	38.65
2797	5.92	16.08	28.29	38.57
2796	5.85	16.01	28.22	38.5
2795	5.78	15.94	28.16	38.43
2794	5.71	15.88	28.09	38.37
2793	5.64	15.81	28.03	38.31
2792	5.57	15.75	27.97	38.25
2791	5.51	15.68	27.91	38.18
2790	5.44	15.62	27.85	38.11
2789	5.39	15.56	27.79	38.05
2788	5.33	15.5	27.74	37.99
2787	5.27	15.44	27.68	37.94
2786	5.21	15.38	27.63	37.89
2785	5.16	15.33	27.58	37.84
2784	5.11	15.28	27.53	37.79
2783	5.05	15.23	27.48	37.74
2782	5	15.18	27.43	37.69
2781	4.95	15.13	27.38	37.64
2780	4.91	15.08	27.33	37.59
2779	4.86	15.03	27.28	37.54
2778	4.81	14.98	27.24	37.49
2777	4.76	14.93	27.2	37.44
2776	4.72	14.88	27.16	37.4
2775	4.67	14.84	27.12	37.36
2774	4.63	14.8	27.08	37.33
2773	4.6	14.77	27.05	37.29
2772	4.57	14.74	27.02	37.26
2771	4.54	14.71	26.98	37.23
2770	4.51	14.67	26.95	37.2
2769	4.48	14.64	26.92	37.17
2768	4.44	14.61	26.9	37.14
2767	4.41	14.59	26.87	37.11
2766	4.39	14.56	26.84	37.08
2765	4.37	14.54	26.81	37.05

2764	4.35	14.52	26.79	37.03
2763	4.32	14.5	26.77	37.01
2762	4.3	14.48	26.74	36.98
2761	4.27	14.45	26.72	36.96
2760	4.24	14.43	26.7	36.94
2759	4.22	14.41	26.68	36.92
2758	4.21	14.39	26.66	36.9
2757	4.2	14.37	26.65	36.89
2756	4.19	14.35	26.63	36.87
2755	4.18	14.33	26.62	36.85
2754	4.17	14.31	26.61	36.82
2753	4.16	14.3	26.59	36.81
2752	4.14	14.28	26.58	36.79
2751	4.12	14.27	26.56	36.78
2750	4.09	14.25	26.53	36.76
2749	4.07	14.24	26.52	36.74
2748	4.06	14.24	26.5	36.73
2747	4.06	14.23	26.5	36.72
2746	4.06	14.23	26.49	36.71
2745	4.05	14.23	26.49	36.7
2744	4.05	14.23	26.48	36.7
2743	4.05	14.23	26.46	36.71
2742	4.06	14.23	26.46	36.71
2741	4.07	14.23	26.46	36.71
2740	4.08	14.24	26.47	36.71
2739	4.08	14.24	26.48	36.71
2738	4.09	14.26	26.5	36.72
2737	4.09	14.27	26.51	36.73
2736	4.11	14.29	26.52	36.74
2735	4.14	14.3	26.54	36.76
2734	4.17	14.32	26.55	36.78
2733	4.2	14.34	26.56	36.8
2732	4.22	14.35	26.57	36.81
2731	4.23	14.36	26.58	36.82
2730	4.23	14.37	26.58	36.82
2729	4.22	14.37	26.58	36.82
2728	4.21	14.36	26.58	36.81
2727	4.19	14.35	26.58	36.8
2726	4.18	14.34	26.56	36.79
2725	4.17	14.32	26.55	36.77
2724	4.15	14.31	26.53	36.75
2723	4.13	14.28	26.51	36.72
2722	4.1	14.26	26.49	36.7
2721	4.08	14.23	26.47	36.68

2720	4.06	14.2	26.45	36.66
2719	4.04	14.18	26.43	36.64
2718	4.01	14.15	26.41	36.62
2717	3.99	14.13	26.38	36.59
2716	3.96	14.11	26.36	36.56
2715	3.93	14.08	26.33	36.54
2714	3.9	14.06	26.3	36.51
2713	3.87	14.03	26.27	36.49
2712	3.85	14	26.25	36.46
2711	3.82	13.97	26.22	36.44
2710	3.8	13.95	26.2	36.42
2709	3.78	13.92	26.18	36.4
2708	3.76	13.9	26.16	36.38
2707	3.74	13.88	26.15	36.37
2706	3.73	13.87	26.13	36.35
2705	3.71	13.86	26.12	36.34
2704	3.69	13.85	26.1	36.33
2703	3.68	13.84	26.09	36.32
2702	3.68	13.83	26.08	36.31
2701	3.67	13.82	26.07	36.3
2700	3.66	13.81	26.06	36.29
2699	3.65	13.81	26.05	36.28
2698	3.64	13.8	26.05	36.27
2697	3.63	13.8	26.05	36.26
2696	3.63	13.79	26.04	36.25
2695	3.63	13.78	26.04	36.24
2694	3.63	13.78	26.03	36.24
2693	3.63	13.78	26.03	36.24
2692	3.63	13.78	26.03	36.24
2691	3.63	13.78	26.03	36.24
2690	3.63	13.78	26.03	36.24
2689	3.63	13.78	26.02	36.23
2688	3.63	13.77	26.01	36.23
2687	3.63	13.77	26.01	36.22
2686	3.62	13.76	26.01	36.22
2685	3.62	13.76	26.01	36.21
2684	3.61	13.76	26.01	36.21
2683	3.6	13.76	26.01	36.21
2682	3.6	13.75	26	36.2
2681	3.59	13.75	26	36.2
2680	3.59	13.74	25.99	36.19
2679	3.59	13.74	25.98	36.19
2678	3.59	13.73	25.98	36.19
2677	3.58	13.73	25.98	36.19

2676	3.58	13.73	25.98	36.19
2675	3.58	13.73	25.98	36.18
2674	3.58	13.73	25.97	36.18
2673	3.57	13.73	25.96	36.18
2672	3.57	13.73	25.96	36.17
2671	3.57	13.73	25.95	36.17
2670	3.56	13.72	25.95	36.16
2669	3.56	13.72	25.95	36.16
2668	3.56	13.71	25.96	36.15
2667	3.56	13.71	25.95	36.14
2666	3.55	13.7	25.95	36.14
2665	3.55	13.69	25.94	36.14
2664	3.55	13.69	25.93	36.13
2663	3.54	13.68	25.93	36.13
2662	3.53	13.68	25.92	36.13
2661	3.52	13.67	25.91	36.12
2660	3.52	13.67	25.9	36.11
2659	3.51	13.66	25.89	36.1
2658	3.5	13.64	25.88	36.1
2657	3.48	13.62	25.87	36.09
2656	3.47	13.61	25.86	36.08
2655	3.46	13.6	25.85	36.07
2654	3.45	13.59	25.84	36.06
2653	3.44	13.58	25.83	36.06
2652	3.43	13.58	25.82	36.05
2651	3.43	13.57	25.8	36.04
2650	3.42	13.56	25.79	36.02
2649	3.41	13.56	25.78	36.01
2648	3.4	13.55	25.77	36
2647	3.38	13.55	25.76	35.99
2646	3.37	13.54	25.75	35.98
2645	3.36	13.53	25.75	35.97
2644	3.35	13.52	25.74	35.96
2643	3.34	13.5	25.74	35.95
2642	3.34	13.49	25.73	35.94
2641	3.33	13.48	25.72	35.94
2640	3.32	13.47	25.71	35.93
2639	3.3	13.46	25.7	35.92
2638	3.29	13.46	25.68	35.91
2637	3.28	13.45	25.67	35.89
2636	3.27	13.44	25.66	35.88
2635	3.27	13.44	25.65	35.87
2634	3.26	13.42	25.64	35.86
2633	3.26	13.41	25.63	35.85

2632	3.25	13.4	25.63	35.84
2631	3.24	13.4	25.62	35.83
2630	3.23	13.39	25.6	35.82
2629	3.23	13.39	25.59	35.82
2628	3.22	13.38	25.58	35.81
2627	3.21	13.38	25.57	35.8
2626	3.2	13.37	25.57	35.79
2625	3.19	13.36	25.57	35.79
2624	3.18	13.34	25.58	35.79
2623	3.17	13.33	25.57	35.78
2622	3.16	13.31	25.57	35.77
2621	3.15	13.3	25.56	35.76
2620	3.14	13.3	25.55	35.75
2619	3.14	13.3	25.54	35.75
2618	3.14	13.3	25.53	35.74
2617	3.14	13.29	25.52	35.74
2616	3.14	13.28	25.51	35.73
2615	3.13	13.27	25.5	35.72
2614	3.11	13.26	25.5	35.71
2613	3.08	13.26	25.49	35.7
2612	3.07	13.26	25.48	35.69
2611	3.06	13.25	25.47	35.68
2610	3.06	13.24	25.47	35.66
2609	3.06	13.23	25.47	35.65
2608	3.06	13.23	25.47	35.65
2607	3.06	13.22	25.46	35.64
2606	3.06	13.22	25.45	35.64
2605	3.05	13.22	25.43	35.63
2604	3.04	13.21	25.42	35.63
2603	3.03	13.2	25.41	35.62
2602	3.01	13.19	25.41	35.61
2601	3	13.17	25.4	35.6
2600	2.99	13.15	25.39	35.59
2599	2.98	13.13	25.38	35.58
2598	2.97	13.12	25.37	35.57
2597	2.96	13.11	25.36	35.57
2596	2.95	13.1	25.35	35.56
2595	2.94	13.1	25.34	35.55
2594	2.93	13.09	25.33	35.55
2593	2.93	13.08	25.32	35.54
2592	2.93	13.06	25.31	35.53
2591	2.92	13.05	25.31	35.52
2590	2.92	13.04	25.31	35.51
2589	2.91	13.04	25.31	35.5

2588	2.9	13.04	25.3	35.49
2587	2.89	13.03	25.29	35.48
2586	2.87	13.03	25.27	35.48
2585	2.86	13.02	25.26	35.48
2584	2.85	13	25.25	35.47
2583	2.84	12.99	25.24	35.47
2582	2.83	12.98	25.23	35.46
2581	2.82	12.97	25.22	35.45
2580	2.82	12.95	25.22	35.43
2579	2.81	12.94	25.21	35.42
2578	2.8	12.92	25.21	35.41
2577	2.79	12.9	25.2	35.4
2576	2.77	12.89	25.2	35.4
2575	2.76	12.88	25.19	35.39
2574	2.75	12.87	25.17	35.38
2573	2.74	12.87	25.16	35.37
2572	2.73	12.88	25.15	35.35
2571	2.73	12.88	25.14	35.34
2570	2.71	12.87	25.13	35.32
2569	2.7	12.86	25.12	35.32
2568	2.69	12.85	25.12	35.31
2567	2.68	12.84	25.11	35.31
2566	2.67	12.82	25.11	35.31
2565	2.66	12.81	25.1	35.31
2564	2.66	12.8	25.1	35.31
2563	2.66	12.79	25.09	35.3
2562	2.66	12.79	25.08	35.29
2561	2.65	12.8	25.08	35.28
2560	2.64	12.8	25.07	35.26
2559	2.63	12.79	25.07	35.25
2558	2.62	12.78	25.06	35.25
2557	2.61	12.78	25.06	35.24
2556	2.6	12.77	25.05	35.23
2555	2.59	12.76	25.04	35.21
2554	2.58	12.75	25.03	35.2
2553	2.58	12.75	25.02	35.19
2552	2.57	12.74	25	35.19
2551	2.56	12.73	24.99	35.19
2550	2.55	12.73	24.98	35.19
2549	2.55	12.73	24.98	35.19
2548	2.55	12.72	24.98	35.19
2547	2.55	12.71	24.97	35.19
2546	2.54	12.7	24.97	35.19
2545	2.54	12.68	24.97	35.19

2544	2.53	12.67	24.96	35.18
2543	2.53	12.67	24.96	35.17
2542	2.53	12.67	24.95	35.15
2541	2.53	12.67	24.95	35.15
2540	2.52	12.68	24.95	35.14
2539	2.51	12.67	24.95	35.14
2538	2.5	12.67	24.94	35.14
2537	2.5	12.66	24.94	35.13
2536	2.5	12.65	24.93	35.12
2535	2.5	12.63	24.93	35.12
2534	2.5	12.62	24.93	35.12
2533	2.49	12.62	24.92	35.12
2532	2.48	12.62	24.91	35.12
2531	2.47	12.62	24.91	35.12
2530	2.47	12.61	24.91	35.11
2529	2.46	12.61	24.91	35.1
2528	2.45	12.6	24.91	35.09
2527	2.44	12.6	24.9	35.08
2526	2.43	12.59	24.9	35.07
2525	2.42	12.58	24.9	35.07
2524	2.42	12.56	24.9	35.07
2523	2.41	12.55	24.89	35.06
2522	2.4	12.54	24.88	35.05
2521	2.4	12.54	24.87	35.04
2520	2.39	12.54	24.86	35.03
2519	2.38	12.54	24.86	35.03
2518	2.38	12.54	24.85	35.03
2517	2.37	12.53	24.85	35.03
2516	2.36	12.52	24.85	35.03
2515	2.36	12.51	24.84	35.03
2514	2.35	12.5	24.83	35.03
2513	2.35	12.5	24.82	35.02
2512	2.35	12.5	24.81	35.01
2511	2.35	12.5	24.8	35.01
2510	2.35	12.49	24.79	35
2509	2.35	12.48	24.79	34.99
2508	2.35	12.47	24.79	34.98
2507	2.35	12.46	24.79	34.98
2506	2.34	12.46	24.79	34.97
2505	2.34	12.47	24.78	34.97
2504	2.33	12.48	24.79	34.98
2503	2.33	12.49	24.79	34.98
2502	2.32	12.49	24.79	34.98
2501	2.31	12.47	24.79	34.98

2500	2.3	12.46	24.79	34.97
2499	2.3	12.44	24.78	34.95
2498	2.3	12.43	24.78	34.94
2497	2.31	12.42	24.78	34.93
2496	2.31	12.41	24.78	34.93
2495	2.31	12.4	24.78	34.93
2494	2.3	12.41	24.77	34.94
2493	2.29	12.41	24.76	34.95
2492	2.28	12.42	24.75	34.95
2491	2.28	12.41	24.74	34.95
2490	2.28	12.4	24.74	34.94
2489	2.28	12.39	24.74	34.92
2488	2.28	12.39	24.74	34.91
2487	2.26	12.38	24.74	34.9
2486	2.26	12.38	24.74	34.9
2485	2.25	12.37	24.74	34.9
2484	2.26	12.38	24.74	34.9
2483	2.26	12.38	24.75	34.9
2482	2.27	12.38	24.75	34.9
2481	2.27	12.38	24.74	34.9
2480	2.27	12.37	24.74	34.9
2479	2.27	12.37	24.72	34.9
2478	2.26	12.36	24.71	34.9
2477	2.26	12.36	24.71	34.9
2476	2.25	12.36	24.71	34.9
2475	2.25	12.36	24.7	34.89
2474	2.24	12.36	24.7	34.88
2473	2.23	12.35	24.69	34.87
2472	2.23	12.35	24.69	34.88
2471	2.23	12.36	24.68	34.88
2470	2.23	12.35	24.69	34.88
2469	2.22	12.35	24.7	34.89
2468	2.21	12.35	24.7	34.89
2467	2.21	12.34	24.7	34.89
2466	2.21	12.33	24.69	34.88
2465	2.21	12.33	24.7	34.88
2464	2.22	12.33	24.7	34.88
2463	2.22	12.34	24.71	34.88
2462	2.22	12.35	24.71	34.88
2461	2.23	12.36	24.7	34.88
2460	2.23	12.36	24.7	34.89
2459	2.23	12.37	24.69	34.88
2458	2.22	12.37	24.69	34.87
2457	2.21	12.37	24.68	34.86

2456	2.2	12.36	24.68	34.86
2455	2.2	12.36	24.67	34.85
2454	2.2	12.34	24.67	34.85
2453	2.2	12.32	24.66	34.84
2452	2.19	12.3	24.66	34.84
2451	2.19	12.29	24.66	34.84
2450	2.18	12.29	24.67	34.83
2449	2.18	12.29	24.67	34.83
2448	2.18	12.3	24.67	34.82
2447	2.18	12.31	24.67	34.82
2446	2.17	12.3	24.66	34.82
2445	2.17	12.3	24.65	34.82
2444	2.16	12.29	24.65	34.82
2443	2.16	12.29	24.65	34.83
2442	2.15	12.29	24.65	34.83
2441	2.16	12.28	24.65	34.84
2440	2.16	12.28	24.66	34.84
2439	2.17	12.28	24.66	34.83
2438	2.17	12.28	24.66	34.81
2437	2.16	12.28	24.66	34.8
2436	2.17	12.27	24.65	34.79
2435	2.17	12.27	24.64	34.79
2434	2.17	12.26	24.64	34.79
2433	2.17	12.27	24.65	34.8
2432	2.16	12.28	24.65	34.8
2431	2.16	12.28	24.65	34.8
2430	2.15	12.28	24.64	34.8
2429	2.14	12.28	24.63	34.8
2428	2.14	12.27	24.62	34.8
2427	2.14	12.26	24.61	34.79
2426	2.14	12.25	24.61	34.79
2425	2.13	12.25	24.61	34.78
2424	2.12	12.26	24.61	34.78
2423	2.12	12.27	24.61	34.77
2422	2.12	12.27	24.61	34.77
2421	2.13	12.26	24.61	34.77
2420	2.14	12.26	24.61	34.78
2419	2.14	12.26	24.61	34.78
2418	2.15	12.26	24.6	34.78
2417	2.15	12.26	24.59	34.77
2416	2.15	12.26	24.59	34.77
2415	2.14	12.25	24.59	34.76
2414	2.14	12.25	24.59	34.76
2413	2.14	12.24	24.6	34.76

2412	2.13	12.24	24.61	34.76
2411	2.13	12.24	24.61	34.76
2410	2.12	12.25	24.6	34.75
2409	2.11	12.24	24.59	34.76
2408	2.11	12.24	24.58	34.77
2407	2.11	12.23	24.57	34.77
2406	2.11	12.22	24.57	34.77
2405	2.11	12.21	24.58	34.76
2404	2.12	12.2	24.59	34.76
2403	2.12	12.21	24.61	34.76
2402	2.12	12.21	24.62	34.76
2401	2.12	12.22	24.62	34.76
2400	2.12	12.22	24.62	34.75
2399	2.12	12.23	24.62	34.74
2398	2.11	12.23	24.61	34.74
2397	2.1	12.22	24.61	34.73
2396	2.09	12.22	24.61	34.73
2395	2.09	12.21	24.6	34.73
2394	2.08	12.2	24.6	34.73
2393	2.09	12.2	24.6	34.73
2392	2.09	12.2	24.6	34.73
2391	2.1	12.2	24.59	34.73
2390	2.1	12.2	24.58	34.74
2389	2.09	12.21	24.57	34.74
2388	2.09	12.21	24.57	34.74
2387	2.08	12.22	24.56	34.74
2386	2.08	12.21	24.56	34.73
2385	2.08	12.21	24.57	34.72
2384	2.09	12.2	24.57	34.71
2383	2.09	12.19	24.57	34.71
2382	2.08	12.19	24.57	34.7
2381	2.07	12.18	24.56	34.69
2380	2.06	12.18	24.56	34.69
2379	2.05	12.17	24.54	34.69
2378	2.05	12.17	24.52	34.7
2377	2.04	12.17	24.51	34.71
2376	2.04	12.17	24.51	34.71
2375	2.04	12.18	24.52	34.72
2374	2.04	12.18	24.54	34.71
2373	2.05	12.18	24.55	34.7
2372	2.06	12.18	24.56	34.7
2371	2.06	12.17	24.56	34.69
2370	2.06	12.16	24.54	34.7
2369	2.07	12.15	24.53	34.7

2368	2.06	12.15	24.52	34.71
2367	2.05	12.16	24.53	34.7
2366	2.04	12.17	24.53	34.69
2365	2.03	12.18	24.54	34.69
2364	2.03	12.18	24.55	34.7
2363	2.03	12.19	24.56	34.7
2362	2.04	12.19	24.56	34.71
2361	2.05	12.18	24.57	34.72
2360	2.06	12.18	24.57	34.74
2359	2.07	12.17	24.57	34.74
2358	2.07	12.18	24.56	34.74
2357	2.08	12.18	24.56	34.73
2356	2.08	12.19	24.56	34.71
2355	2.08	12.18	24.57	34.71
2354	2.08	12.18	24.58	34.71
2353	2.07	12.18	24.58	34.72
2352	2.07	12.18	24.57	34.73
2351	2.07	12.18	24.57	34.73
2350	2.07	12.18	24.58	34.72
2349	2.07	12.18	24.59	34.71
2348	2.07	12.19	24.6	34.7
2347	2.06	12.19	24.6	34.7
2346	2.05	12.19	24.59	34.7
2345	2.06	12.18	24.58	34.72
2344	2.08	12.19	24.59	34.73
2343	2.1	12.19	24.6	34.74
2342	2.12	12.19	24.61	34.75
2341	2.12	12.19	24.62	34.76
2340	2.12	12.19	24.61	34.77
2339	2.11	12.2	24.61	34.77
2338	2.09	12.21	24.6	34.76
2337	2.09	12.22	24.6	34.76
2336	2.09	12.22	24.61	34.76
2335	2.09	12.21	24.6	34.76
2334	2.09	12.2	24.6	34.76
2333	2.1	12.19	24.58	34.75
2332	2.1	12.19	24.58	34.75
2331	2.11	12.19	24.58	34.75
2330	2.11	12.19	24.59	34.76
2329	2.12	12.2	24.61	34.77
2328	2.13	12.22	24.63	34.8
2327	2.16	12.26	24.67	34.83
2326	2.18	12.3	24.7	34.86
2325	2.2	12.32	24.72	34.89

2324	2.21	12.32	24.72	34.9
2323	2.19	12.3	24.69	34.88
2322	2.17	12.28	24.65	34.86
2321	2.14	12.25	24.62	34.84
2320	2.12	12.23	24.61	34.83
2319	2.1	12.22	24.6	34.82
2318	2.09	12.21	24.59	34.81
2317	2.08	12.2	24.58	34.8
2316	2.08	12.2	24.58	34.79
2315	2.08	12.2	24.58	34.78
2314	2.07	12.2	24.57	34.76
2313	2.07	12.19	24.56	34.75
2312	2.06	12.18	24.55	34.74
2311	2.06	12.16	24.54	34.73
2310	2.05	12.14	24.54	34.73
2309	2.05	12.14	24.53	34.73
2308	2.05	12.14	24.54	34.74
2307	2.05	12.14	24.54	34.74
2306	2.05	12.14	24.55	34.74
2305	2.05	12.14	24.55	34.73
2304	2.05	12.14	24.55	34.73
2303	2.04	12.14	24.55	34.72
2302	2.03	12.13	24.54	34.71
2301	2.02	12.12	24.53	34.7
2300	2.02	12.12	24.53	34.69
2299	2.02	12.13	24.52	34.68
2298	2.01	12.14	24.52	34.67
2297	2.01	12.14	24.52	34.66
2296	2	12.15	24.52	34.65
2295	2	12.14	24.53	34.65
2294	2	12.14	24.53	34.65
2293	2.01	12.14	24.55	34.65
2292	2.02	12.14	24.56	34.67
2291	2.03	12.14	24.56	34.68
2290	2.03	12.15	24.55	34.69
2289	2.03	12.15	24.54	34.69
2288	2.03	12.15	24.52	34.69
2287	2.03	12.15	24.52	34.68
2286	2.03	12.14	24.52	34.67
2285	2.02	12.13	24.51	34.66
2284	2.01	12.11	24.5	34.66
2283	2	12.1	24.49	34.65
2282	1.97	12.08	24.47	34.64
2281	1.96	12.07	24.46	34.63

2280	1.94	12.06	24.45	34.62
2279	1.93	12.06	24.45	34.62
2278	1.93	12.05	24.44	34.62
2277	1.93	12.05	24.43	34.62
2276	1.93	12.05	24.42	34.62
2275	1.93	12.03	24.41	34.6
2274	1.92	12	24.4	34.58
2273	1.9	11.98	24.39	34.56
2272	1.88	11.97	24.38	34.56
2271	1.87	11.97	24.38	34.56
2270	1.85	11.97	24.38	34.56
2269	1.84	11.98	24.38	34.55
2268	1.83	11.96	24.39	34.54
2267	1.82	11.94	24.39	34.51
2266	1.81	11.92	24.38	34.48
2265	1.81	11.91	24.38	34.46
2264	1.81	11.92	24.38	34.46
2263	1.81	11.92	24.38	34.46
2262	1.82	11.93	24.37	34.47
2261	1.83	11.93	24.34	34.49
2260	1.84	11.93	24.32	34.5
2259	1.84	11.92	24.32	34.5
2258	1.83	11.9	24.33	34.5
2257	1.82	11.9	24.35	34.49
2256	1.81	11.9	24.36	34.48
2255	1.79	11.9	24.34	34.47
2254	1.76	11.89	24.31	34.46
2253	1.74	11.88	24.28	34.44
2252	1.72	11.87	24.27	34.43
2251	1.71	11.86	24.26	34.43
2250	1.71	11.86	24.26	34.44
2249	1.73	11.86	24.26	34.45
2248	1.76	11.86	24.28	34.46
2247	1.79	11.84	24.28	34.46
2246	1.82	11.82	24.28	34.46
2245	1.82	11.81	24.28	34.45
2244	1.82	11.83	24.29	34.44
2243	1.8	11.85	24.29	34.42
2242	1.78	11.86	24.28	34.4
2241	1.76	11.87	24.28	34.39
2240	1.75	11.85	24.27	34.4
2239	1.75	11.83	24.27	34.41
2238	1.74	11.81	24.27	34.44
2237	1.74	11.81	24.26	34.47

2236	1.75	11.81	24.25	34.48
2235	1.75	11.8	24.24	34.48
2234	1.74	11.78	24.24	34.45
2233	1.73	11.78	24.25	34.42
2232	1.72	11.79	24.25	34.39
2231	1.69	11.8	24.25	34.38
2230	1.66	11.8	24.23	34.36
2229	1.63	11.8	24.23	34.36
2228	1.62	11.79	24.22	34.37
2227	1.6	11.79	24.21	34.37
2226	1.6	11.77	24.2	34.38
2225	1.61	11.74	24.2	34.39
2224	1.63	11.73	24.19	34.39
2223	1.64	11.73	24.18	34.38
2222	1.63	11.74	24.16	34.38
2221	1.63	11.76	24.13	34.37
2220	1.64	11.79	24.13	34.37
2219	1.64	11.8	24.13	34.36
2218	1.63	11.79	24.15	34.34
2217	1.61	11.77	24.18	34.32
2216	1.6	11.76	24.2	34.3
2215	1.6	11.75	24.21	34.29
2214	1.6	11.74	24.21	34.29
2213	1.61	11.73	24.22	34.31
2212	1.61	11.72	24.23	34.34
2211	1.6	11.71	24.23	34.35
2210	1.6	11.72	24.23	34.34
2209	1.6	11.72	24.21	34.32
2208	1.61	11.74	24.19	34.32
2207	1.62	11.75	24.17	34.32
2206	1.64	11.77	24.16	34.31
2205	1.67	11.79	24.15	34.32
2204	1.69	11.79	24.15	34.32
2203	1.69	11.76	24.13	34.31
2202	1.66	11.73	24.11	34.29
2201	1.61	11.72	24.1	34.27
2200	1.58	11.74	24.11	34.26
2199	1.56	11.75	24.12	34.25
2198	1.56	11.76	24.13	34.25
2197	1.57	11.77	24.14	34.28
2196	1.58	11.79	24.16	34.32
2195	1.59	11.78	24.16	34.34
2194	1.59	11.77	24.15	34.34
2193	1.6	11.75	24.15	34.34

2192	1.63	11.73	24.14	34.33
2191	1.66	11.71	24.13	34.32
2190	1.69	11.72	24.12	34.3
2189	1.7	11.74	24.13	34.28
2188	1.7	11.75	24.16	34.27
2187	1.67	11.74	24.17	34.28
2186	1.63	11.71	24.17	34.3
2185	1.6	11.7	24.16	34.32
2184	1.59	11.7	24.15	34.31
2183	1.56	11.69	24.13	34.29
2182	1.51	11.65	24.09	34.27
2181	1.47	11.62	24.06	34.28
2180	1.46	11.6	24.06	34.31
2179	1.46	11.59	24.06	34.33
2178	1.48	11.56	24.05	34.31
2177	1.5	11.54	24.03	34.25
2176	1.49	11.52	24.03	34.17
2175	1.46	11.51	24.04	34.09
2174	1.43	11.51	24.07	34.04
2173	1.42	11.55	24.1	34.06
2172	1.43	11.59	24.13	34.14
2171	1.46	11.62	24.13	34.23
2170	1.49	11.61	24.11	34.29
2169	1.52	11.6	24.08	34.3
2168	1.53	11.62	24.07	34.29
2167	1.52	11.65	24.07	34.29
2166	1.5	11.69	24.08	34.3
2165	1.5	11.74	24.1	34.35
2164	1.53	11.8	24.12	34.39
2163	1.54	11.82	24.12	34.41
2162	1.53	11.77	24.08	34.38
2161	1.49	11.64	24.01	34.27
2160	1.41	11.49	23.91	34.09
2159	1.31	11.35	23.81	33.87
2158	1.22	11.26	23.74	33.67
2157	1.19	11.24	23.74	33.58
2156	1.22	11.24	23.79	33.58
2155	1.27	11.24	23.83	33.65
2154	1.28	11.23	23.85	33.75
2153	1.29	11.26	23.87	33.85
2152	1.28	11.33	23.87	33.92
2151	1.27	11.39	23.87	33.95
2150	1.27	11.42	23.85	33.94
2149	1.29	11.42	23.84	33.94

2148	1.31	11.41	23.86	33.94
2147	1.34	11.38	23.87	33.93
2146	1.35	11.35	23.88	33.92
2145	1.37	11.32	23.9	33.91
2144	1.38	11.31	23.91	33.92
2143	1.36	11.31	23.9	33.95
2142	1.33	11.32	23.89	33.98
2141	1.32	11.35	23.88	34.03
2140	1.32	11.38	23.89	34.07
2139	1.31	11.4	23.9	34.09
2138	1.3	11.4	23.89	34.08
2137	1.29	11.39	23.89	34.04
2136	1.29	11.35	23.89	33.99
2135	1.29	11.31	23.88	33.94
2134	1.29	11.28	23.88	33.91
2133	1.3	11.29	23.89	33.91
2132	1.33	11.33	23.9	33.96
2131	1.36	11.36	23.91	34.01
2130	1.39	11.39	23.9	34.05
2129	1.41	11.43	23.91	34.07
2128	1.41	11.47	23.93	34.08
2127	1.39	11.52	23.95	34.1
2126	1.37	11.55	23.98	34.11
2125	1.35	11.57	24	34.13
2124	1.37	11.57	24.02	34.14
2123	1.38	11.55	24.03	34.15
2122	1.4	11.53	24.04	34.14
2121	1.42	11.5	24.05	34.14
2120	1.44	11.5	24.06	34.14
2119	1.46	11.5	24.07	34.14
2118	1.47	11.52	24.08	34.14
2117	1.49	11.56	24.09	34.17
2116	1.5	11.6	24.11	34.2
2115	1.5	11.62	24.11	34.23
2114	1.49	11.63	24.1	34.23
2113	1.48	11.63	24.08	34.22
2112	1.47	11.61	24.08	34.21
2111	1.47	11.59	24.07	34.2
2110	1.47	11.56	24.07	34.18
2109	1.48	11.54	24.06	34.17
2108	1.49	11.53	24.06	34.16
2107	1.49	11.54	24.07	34.16
2106	1.48	11.55	24.08	34.16
2105	1.47	11.57	24.08	34.18

2104	1.48	11.59	24.1	34.19
2103	1.49	11.59	24.1	34.2
2102	1.5	11.58	24.1	34.2
2101	1.52	11.58	24.09	34.21
2100	1.53	11.59	24.08	34.22
2099	1.53	11.59	24.08	34.23
2098	1.52	11.57	24.07	34.21
2097	1.49	11.55	24.06	34.18
2096	1.46	11.53	24.04	34.14
2095	1.44	11.52	24	34.11
2094	1.42	11.52	23.98	34.1
2093	1.43	11.54	23.97	34.1
2092	1.44	11.56	23.98	34.12
2091	1.45	11.57	23.98	34.14
2090	1.46	11.57	23.98	34.15
2089	1.47	11.57	24	34.17
2088	1.49	11.57	24.02	34.18
2087	1.5	11.58	24.03	34.18
2086	1.49	11.58	24.02	34.17
2085	1.49	11.58	24.01	34.17
2084	1.48	11.58	24	34.18
2083	1.48	11.58	24.01	34.19
2082	1.47	11.58	24.03	34.2
2081	1.48	11.57	24.05	34.21
2080	1.48	11.57	24.06	34.21
2079	1.48	11.56	24.05	34.19
2078	1.45	11.55	24.05	34.17
2077	1.43	11.56	24.06	34.16
2076	1.43	11.56	24.07	34.17
2075	1.43	11.57	24.07	34.18
2074	1.43	11.56	24.05	34.18
2073	1.44	11.56	24.02	34.18
2072	1.45	11.55	23.99	34.17
2071	1.46	11.55	23.98	34.16
2070	1.47	11.55	24	34.14
2069	1.47	11.57	24.02	34.13
2068	1.47	11.58	24.04	34.12
2067	1.46	11.58	24.03	34.1
2066	1.44	11.57	24	34.09
2065	1.42	11.57	23.98	34.1
2064	1.42	11.56	23.98	34.13
2063	1.41	11.56	23.99	34.14
2062	1.4	11.55	24.01	34.13
2061	1.4	11.54	24.02	34.11

2060	1.42	11.53	24.02	34.11
2059	1.45	11.51	24	34.12
2058	1.47	11.51	23.98	34.14
2057	1.49	11.51	23.96	34.15
2056	1.5	11.51	23.96	34.15
2055	1.5	11.51	23.96	34.14
2054	1.49	11.5	23.99	34.13
2053	1.5	11.53	24.04	34.17
2052	1.52	11.59	24.11	34.22
2051	1.53	11.63	24.14	34.25
2050	1.51	11.62	24.12	34.22
2049	1.49	11.59	24.07	34.16
2048	1.46	11.56	24.02	34.12
2047	1.43	11.54	23.98	34.08
2046	1.41	11.55	23.96	34.04
2045	1.38	11.57	23.96	34.01
2044	1.37	11.59	23.97	34.02
2043	1.35	11.58	23.98	34.06
2042	1.34	11.56	23.97	34.09
2041	1.36	11.53	23.97	34.1
2040	1.39	11.51	23.97	34.11
2039	1.42	11.48	23.97	34.1
2038	1.44	11.46	23.98	34.08
2037	1.44	11.45	24.01	34.08
2036	1.42	11.43	24.02	34.07
2035	1.36	11.39	23.98	34.03
2034	1.31	11.33	23.9	33.98
2033	1.29	11.31	23.85	33.93
2032	1.3	11.34	23.82	33.91
2031	1.31	11.37	23.8	33.91
2030	1.31	11.39	23.78	33.91
2029	1.33	11.39	23.78	33.94
2028	1.35	11.38	23.8	33.96
2027	1.37	11.35	23.81	33.95
2026	1.38	11.3	23.81	33.94
2025	1.38	11.27	23.82	33.95
2024	1.37	11.3	23.83	33.96
2023	1.31	11.34	23.83	33.95
2022	1.23	11.37	23.81	33.93
2021	1.17	11.38	23.81	33.91
2020	1.15	11.38	23.8	33.92
2019	1.17	11.37	23.77	33.92
2018	1.2	11.36	23.72	33.92
2017	1.25	11.35	23.7	33.93

2016	1.29	11.33	23.72	33.94
2015	1.28	11.31	23.75	33.93
2014	1.22	11.31	23.75	33.91
2013	1.17	11.32	23.75	33.9
2012	1.16	11.34	23.74	33.92
2011	1.17	11.33	23.72	33.94
2010	1.16	11.31	23.69	33.94
2009	1.14	11.29	23.67	33.94
2008	1.1	11.26	23.68	33.93
2007	1.05	11.21	23.7	33.89
2006	1.03	11.16	23.72	33.84
2005	1.06	11.16	23.74	33.82
2004	1.12	11.2	23.74	33.82
2003	1.18	11.25	23.74	33.82
2002	1.2	11.28	23.74	33.82
2001	1.21	11.29	23.77	33.85
2000	1.21	11.29	23.82	33.9
1999	1.2	11.28	23.86	33.94
1998	1.19	11.28	23.87	33.94
1997	1.21	11.3	23.86	33.94
1996	1.25	11.33	23.86	33.93
1995	1.25	11.33	23.85	33.92
1994	1.23	11.32	23.85	33.9
1993	1.2	11.33	23.87	33.91
1992	1.2	11.36	23.9	33.95
1991	1.22	11.36	23.89	33.99
1990	1.27	11.35	23.86	34.01
1989	1.33	11.35	23.83	34.03
1988	1.36	11.36	23.83	34.04
1987	1.33	11.39	23.83	34.05
1986	1.27	11.41	23.82	34.04
1985	1.24	11.43	23.82	34.05
1984	1.27	11.46	23.84	34.05
1983	1.34	11.46	23.87	34.05
1982	1.4	11.46	23.91	34.03
1981	1.44	11.47	23.95	34.03
1980	1.43	11.49	23.95	34.02
1979	1.36	11.47	23.91	33.97
1978	1.24	11.41	23.83	33.89
1977	1.14	11.33	23.76	33.84
1976	1.09	11.25	23.73	33.81
1975	1.07	11.19	23.7	33.77
1974	1.05	11.16	23.69	33.72
1973	1.05	11.18	23.69	33.69

1972	1.08	11.22	23.7	33.7
1971	1.12	11.24	23.7	33.73
1970	1.16	11.23	23.7	33.76
1969	1.19	11.19	23.71	33.79
1968	1.22	11.17	23.75	33.82
1967	1.22	11.15	23.76	33.84
1966	1.2	11.18	23.76	33.85
1965	1.18	11.26	23.77	33.87
1964	1.18	11.34	23.77	33.9
1963	1.17	11.35	23.75	33.9
1962	1.15	11.31	23.71	33.89
1961	1.14	11.25	23.68	33.88
1960	1.13	11.21	23.68	33.89
1959	1.11	11.19	23.7	33.89
1958	1.11	11.18	23.72	33.88
1957	1.13	11.21	23.75	33.87
1956	1.17	11.27	23.78	33.86
1955	1.19	11.31	23.8	33.84
1954	1.19	11.33	23.78	33.81
1953	1.2	11.34	23.78	33.8
1952	1.22	11.34	23.78	33.8
1951	1.24	11.33	23.79	33.81
1950	1.25	11.3	23.8	33.82
1949	1.27	11.28	23.82	33.84
1948	1.29	11.29	23.85	33.87
1947	1.29	11.3	23.85	33.89
1946	1.28	11.32	23.85	33.9
1945	1.28	11.35	23.84	33.91
1944	1.31	11.39	23.86	33.93
1943	1.34	11.42	23.87	33.93
1942	1.37	11.42	23.88	33.93
1941	1.39	11.41	23.88	33.95
1940	1.39	11.4	23.88	33.98
1939	1.38	11.39	23.88	34
1938	1.34	11.38	23.88	33.99
1937	1.32	11.39	23.89	33.98
1936	1.31	11.4	23.9	33.98
1935	1.32	11.4	23.89	33.99
1934	1.34	11.4	23.87	33.98
1933	1.38	11.4	23.87	33.98
1932	1.4	11.42	23.9	33.97
1931	1.4	11.44	23.92	33.96
1930	1.38	11.44	23.93	33.96
1929	1.38	11.45	23.92	33.98

1928	1.39	11.46	23.92	34.01
1927	1.4	11.48	23.92	34.03
1926	1.41	11.5	23.94	34.04
1925	1.42	11.53	23.96	34.04
1924	1.45	11.56	23.98	34.04
1923	1.47	11.58	23.99	34.04
1922	1.47	11.59	23.99	34.05
1921	1.48	11.6	24	34.07
1920	1.49	11.61	24.01	34.09
1919	1.5	11.62	24.03	34.1
1918	1.5	11.62	24.04	34.09
1917	1.5	11.63	24.06	34.1
1916	1.51	11.65	24.08	34.11
1915	1.52	11.66	24.08	34.11
1914	1.53	11.65	24.07	34.11
1913	1.55	11.63	24.06	34.12
1912	1.57	11.63	24.07	34.14
1911	1.57	11.62	24.08	34.16
1910	1.57	11.62	24.1	34.17
1909	1.58	11.64	24.11	34.18
1908	1.6	11.66	24.12	34.19
1907	1.6	11.67	24.11	34.19
1906	1.59	11.67	24.1	34.18
1905	1.58	11.67	24.1	34.18
1904	1.58	11.66	24.11	34.18
1903	1.58	11.65	24.11	34.17
1902	1.58	11.65	24.1	34.17
1901	1.58	11.66	24.1	34.17
1900	1.59	11.69	24.1	34.18
1899	1.59	11.69	24.1	34.19
1898	1.6	11.69	24.1	34.2
1897	1.6	11.69	24.1	34.21
1896	1.61	11.69	24.1	34.22
1895	1.61	11.69	24.11	34.22
1894	1.61	11.7	24.11	34.21
1893	1.61	11.7	24.13	34.2
1892	1.62	11.71	24.14	34.19
1891	1.62	11.71	24.15	34.2
1890	1.62	11.71	24.14	34.2
1889	1.61	11.71	24.14	34.21
1888	1.61	11.71	24.13	34.23
1887	1.6	11.7	24.12	34.23
1886	1.59	11.69	24.12	34.23
1885	1.59	11.69	24.12	34.23

1884	1.6	11.71	24.12	34.23
1883	1.61	11.71	24.13	34.23
1882	1.61	11.71	24.13	34.21
1881	1.61	11.71	24.14	34.19
1880	1.61	11.72	24.14	34.17
1879	1.6	11.72	24.14	34.16
1878	1.6	11.71	24.13	34.16
1877	1.59	11.7	24.12	34.16
1876	1.6	11.71	24.11	34.18
1875	1.6	11.71	24.1	34.19
1874	1.61	11.71	24.09	34.2
1873	1.63	11.73	24.09	34.21
1872	1.64	11.74	24.1	34.23
1871	1.65	11.75	24.11	34.23
1870	1.63	11.75	24.12	34.23
1869	1.61	11.74	24.12	34.24
1868	1.59	11.74	24.13	34.24
1867	1.59	11.74	24.13	34.23
1866	1.59	11.73	24.14	34.21
1865	1.59	11.71	24.13	34.21
1864	1.6	11.69	24.13	34.21
1863	1.6	11.67	24.12	34.21
1862	1.59	11.66	24.11	34.21
1861	1.59	11.66	24.1	34.2
1860	1.59	11.67	24.11	34.2
1859	1.59	11.68	24.11	34.19
1858	1.59	11.69	24.11	34.18
1857	1.59	11.7	24.11	34.18
1856	1.6	11.71	24.12	34.18
1855	1.6	11.71	24.12	34.17
1854	1.61	11.7	24.11	34.16
1853	1.61	11.68	24.1	34.16
1852	1.61	11.67	24.11	34.16
1851	1.61	11.67	24.11	34.16
1850	1.59	11.66	24.11	34.16
1849	1.58	11.66	24.11	34.17
1848	1.58	11.67	24.11	34.18
1847	1.58	11.68	24.11	34.19
1846	1.58	11.68	24.11	34.18
1845	1.58	11.68	24.12	34.17
1844	1.58	11.68	24.13	34.16
1843	1.58	11.69	24.13	34.16
1842	1.57	11.69	24.12	34.16
1841	1.56	11.7	24.12	34.16

1840	1.56	11.7	24.12	34.16
1839	1.56	11.69	24.13	34.17
1838	1.56	11.68	24.12	34.17
1837	1.56	11.67	24.12	34.17
1836	1.57	11.67	24.12	34.17
1835	1.58	11.68	24.12	34.17
1834	1.59	11.68	24.12	34.16
1833	1.59	11.68	24.13	34.16
1832	1.6	11.69	24.14	34.17
1831	1.6	11.69	24.15	34.19
1830	1.59	11.69	24.14	34.19
1829	1.58	11.69	24.13	34.2
1828	1.58	11.7	24.13	34.21
1827	1.58	11.71	24.12	34.2
1826	1.59	11.7	24.12	34.2
1825	1.59	11.7	24.12	34.2
1824	1.59	11.7	24.13	34.21
1823	1.59	11.7	24.13	34.21
1822	1.59	11.69	24.12	34.2
1821	1.59	11.69	24.12	34.19
1820	1.58	11.69	24.12	34.18
1819	1.57	11.68	24.12	34.17
1818	1.56	11.67	24.11	34.15
1817	1.55	11.67	24.1	34.14
1816	1.56	11.68	24.09	34.15
1815	1.58	11.69	24.09	34.17
1814	1.59	11.7	24.09	34.19
1813	1.6	11.71	24.1	34.2
1812	1.61	11.72	24.11	34.21
1811	1.61	11.72	24.12	34.22
1810	1.61	11.72	24.12	34.21
1809	1.62	11.73	24.12	34.21
1808	1.63	11.73	24.13	34.22
1807	1.64	11.74	24.14	34.23
1806	1.64	11.75	24.14	34.24
1805	1.65	11.76	24.15	34.25
1804	1.66	11.78	24.17	34.27
1803	1.67	11.8	24.18	34.28
1802	1.68	11.8	24.18	34.28
1801	1.68	11.8	24.19	34.29
1800	1.68	11.8	24.2	34.29
1799	1.68	11.8	24.2	34.29
1798	1.68	11.8	24.2	34.28
1797	1.69	11.81	24.21	34.28

1796	1.7	11.83	24.22	34.29
1795	1.71	11.84	24.23	34.29
1794	1.72	11.85	24.24	34.29
1793	1.72	11.85	24.25	34.3
1792	1.73	11.86	24.27	34.31
1791	1.73	11.88	24.27	34.32
1790	1.73	11.88	24.27	34.33
1789	1.75	11.88	24.26	34.34
1788	1.77	11.89	24.27	34.35
1787	1.78	11.89	24.27	34.35
1786	1.79	11.9	24.27	34.35
1785	1.79	11.91	24.28	34.36
1784	1.8	11.93	24.3	34.38
1783	1.8	11.95	24.31	34.39
1782	1.81	11.96	24.31	34.4
1781	1.82	11.97	24.31	34.41
1780	1.84	11.99	24.32	34.43
1779	1.85	11.99	24.32	34.45
1778	1.86	11.99	24.33	34.45
1777	1.87	12	24.35	34.45
1776	1.89	12.02	24.38	34.47
1775	1.91	12.04	24.4	34.48
1774	1.93	12.06	24.42	34.49
1773	1.95	12.07	24.43	34.5
1772	1.97	12.1	24.45	34.53
1771	1.98	12.11	24.46	34.55
1770	1.97	12.12	24.46	34.55
1769	1.98	12.13	24.47	34.55
1768	1.98	12.14	24.48	34.56
1767	1.99	12.15	24.48	34.56
1766	2	12.16	24.49	34.57
1765	2.02	12.17	24.51	34.59
1764	2.05	12.19	24.53	34.61
1763	2.07	12.2	24.54	34.63
1762	2.08	12.21	24.55	34.64
1761	2.09	12.22	24.56	34.65
1760	2.1	12.24	24.57	34.66
1759	2.11	12.27	24.58	34.66
1758	2.11	12.28	24.58	34.65
1757	2.12	12.28	24.58	34.66
1756	2.13	12.29	24.59	34.67
1755	2.13	12.3	24.6	34.69
1754	2.13	12.3	24.61	34.71
1753	2.14	12.31	24.62	34.73

1752	2.15	12.33	24.63	34.75
1751	2.15	12.34	24.64	34.76
1750	2.16	12.34	24.65	34.76
1749	2.17	12.36	24.66	34.76
1748	2.19	12.37	24.67	34.76
1747	2.19	12.38	24.67	34.77
1746	2.19	12.39	24.67	34.77
1745	2.2	12.4	24.68	34.78
1744	2.21	12.41	24.7	34.8
1743	2.22	12.43	24.72	34.82
1742	2.22	12.44	24.73	34.84
1741	2.24	12.45	24.74	34.86
1740	2.27	12.47	24.76	34.88
1739	2.29	12.48	24.77	34.89
1738	2.3	12.5	24.79	34.91
1737	2.31	12.53	24.81	34.92
1736	2.32	12.56	24.83	34.93
1735	2.32	12.58	24.84	34.93
1734	2.3	12.58	24.84	34.94
1733	2.29	12.6	24.85	34.96
1732	2.28	12.62	24.87	34.98
1731	2.27	12.62	24.87	34.99
1730	2.26	12.62	24.87	35.01
1729	2.26	12.63	24.88	35.02
1728	2.26	12.63	24.89	35.03
1727	2.27	12.64	24.9	35.03
1726	2.27	12.64	24.91	35.03
1725	2.28	12.65	24.92	35.04
1724	2.29	12.67	24.94	35.06
1723	2.31	12.7	24.97	35.09
1722	2.33	12.72	24.99	35.11
1721	2.35	12.76	25.02	35.14
1720	2.39	12.8	25.06	35.18
1719	2.44	12.83	25.08	35.21
1718	2.48	12.85	25.1	35.25
1717	2.52	12.9	25.14	35.31
1716	2.57	12.96	25.19	35.37
1715	2.61	13	25.23	35.4
1714	2.65	13.03	25.25	35.43
1713	2.71	13.06	25.28	35.45
1712	2.76	13.1	25.32	35.49
1711	2.8	13.15	25.37	35.53
1710	2.84	13.2	25.41	35.57
1709	2.89	13.26	25.46	35.63

1708	2.94	13.33	25.51	35.68
1707	2.98	13.4	25.55	35.74
1706	3.02	13.46	25.59	35.8
1705	3.05	13.52	25.64	35.87
1704	3.08	13.57	25.69	35.93
1703	3.1	13.62	25.72	35.96
1702	3.12	13.66	25.74	35.98
1701	3.13	13.7	25.76	36
1700	3.16	13.74	25.78	36.05
1699	3.18	13.76	25.81	36.1
1698	3.19	13.77	25.82	36.12
1697	3.21	13.78	25.83	36.13
1696	3.23	13.8	25.85	36.15
1695	3.24	13.81	25.87	36.16
1694	3.24	13.81	25.88	36.17
1693	3.25	13.81	25.89	36.18
1692	3.27	13.81	25.9	36.19
1691	3.28	13.81	25.91	36.2
1690	3.29	13.82	25.91	36.2
1689	3.3	13.82	25.92	36.21
1688	3.31	13.83	25.94	36.23
1687	3.32	13.84	25.95	36.23
1686	3.31	13.85	25.95	36.22
1685	3.31	13.86	25.95	36.22
1684	3.32	13.87	25.95	36.23
1683	3.34	13.87	25.95	36.24
1682	3.34	13.85	25.95	36.23
1681	3.35	13.83	25.95	36.23
1680	3.36	13.83	25.95	36.23
1679	3.37	13.83	25.96	36.24
1678	3.39	13.84	25.97	36.25
1677	3.41	13.85	25.99	36.25
1676	3.44	13.88	26.01	36.26
1675	3.46	13.89	26.02	36.27
1674	3.48	13.9	26.03	36.28
1673	3.49	13.92	26.06	36.29
1672	3.52	13.95	26.08	36.3
1671	3.55	13.97	26.11	36.32
1670	3.58	13.99	26.13	36.36
1669	3.62	14.01	26.15	36.4
1668	3.65	14.03	26.17	36.43
1667	3.67	14.03	26.19	36.45
1666	3.68	14.05	26.21	36.46
1665	3.71	14.07	26.23	36.47

1664	3.74	14.11	26.27	36.5
1663	3.78	14.14	26.3	36.53
1662	3.82	14.17	26.33	36.56
1661	3.86	14.19	26.36	36.6
1660	3.9	14.22	26.4	36.63
1659	3.93	14.25	26.44	36.66
1658	3.97	14.29	26.48	36.69
1657	4.01	14.34	26.53	36.73
1656	4.06	14.4	26.59	36.77
1655	4.1	14.45	26.64	36.8
1654	4.15	14.48	26.67	36.82
1653	4.23	14.52	26.72	36.89
1652	4.28	14.56	26.75	36.96
1651	4.3	14.57	26.76	36.99
1650	4.31	14.58	26.77	37
1649	4.33	14.61	26.79	37.01
1648	4.36	14.64	26.82	37.04
1647	4.4	14.68	26.86	37.06
1646	4.44	14.72	26.89	37.09
1645	4.47	14.74	26.92	37.11
1644	4.51	14.76	26.94	37.13
1643	4.53	14.77	26.97	37.15
1642	4.56	14.8	26.99	37.18
1641	4.59	14.83	27.03	37.21
1640	4.62	14.87	27.07	37.25
1639	4.65	14.9	27.1	37.27
1638	4.67	14.92	27.12	37.28
1637	4.69	14.93	27.14	37.3
1636	4.73	14.96	27.17	37.34
1635	4.76	14.98	27.19	37.38
1634	4.78	14.98	27.21	37.4
1633	4.8	15	27.23	37.42
1632	4.83	15.03	27.26	37.45
1631	4.87	15.07	27.3	37.48
1630	4.92	15.13	27.35	37.53
1629	4.98	15.2	27.4	37.59
1628	5.06	15.28	27.48	37.66
1627	5.12	15.35	27.55	37.73
1626	5.17	15.41	27.61	37.79
1625	5.23	15.47	27.67	37.85
1624	5.32	15.55	27.75	37.93
1623	5.42	15.63	27.83	38.02
1622	5.5	15.69	27.89	38.1
1621	5.57	15.74	27.95	38.16

1620	5.62	15.79	28	38.23
1619	5.68	15.85	28.06	38.29
1618	5.75	15.92	28.12	38.36
1617	5.85	16.02	28.21	38.46
1616	5.97	16.14	28.32	38.57
1615	6.06	16.22	28.4	38.65
1614	6.11	16.28	28.46	38.71
1613	6.17	16.34	28.51	38.76
1612	6.24	16.42	28.58	38.83
1611	6.31	16.49	28.65	38.91
1610	6.38	16.56	28.72	38.99
1609	6.46	16.64	28.79	39.07
1608	6.54	16.72	28.86	39.16
1607	6.61	16.8	28.94	39.23
1606	6.68	16.88	29	39.3
1605	6.74	16.95	29.07	39.36
1604	6.81	17.03	29.14	39.43
1603	6.87	17.09	29.2	39.49
1602	6.91	17.13	29.25	39.53
1601	6.93	17.15	29.27	39.55
1600	6.93	17.15	29.28	39.56
1599	6.92	17.13	29.27	39.54
1598	6.88	17.09	29.24	39.52
1597	6.83	17.05	29.2	39.49
1596	6.79	17.01	29.16	39.46
1595	6.74	16.96	29.12	39.42
1594	6.69	16.9	29.06	39.36
1593	6.64	16.84	29.01	39.32
1592	6.6	16.8	28.97	39.28
1591	6.56	16.76	28.94	39.24
1590	6.51	16.72	28.9	39.2
1589	6.47	16.68	28.86	39.16
1588	6.43	16.65	28.82	39.12
1587	6.39	16.62	28.79	39.09
1586	6.35	16.58	28.75	39.05
1585	6.31	16.53	28.72	39.01
1584	6.28	16.49	28.69	38.98
1583	6.24	16.45	28.66	38.94
1582	6.2	16.41	28.62	38.9
1581	6.15	16.37	28.58	38.86
1580	6.12	16.34	28.56	38.83
1579	6.09	16.3	28.53	38.8
1578	6.05	16.26	28.5	38.77
1577	6.01	16.2	28.45	38.72

1576	5.97	16.16	28.4	38.67
1575	5.92	16.11	28.36	38.63
1574	5.87	16.07	28.31	38.58
1573	5.83	16.04	28.28	38.54
1572	5.79	16.01	28.26	38.51
1571	5.73	15.97	28.21	38.45
1570	5.65	15.89	28.14	38.37
1569	5.57	15.79	28.06	38.29
1568	5.5	15.72	27.99	38.23
1567	5.45	15.65	27.93	38.17
1566	5.38	15.58	27.86	38.1
1565	5.32	15.51	27.79	38.04
1564	5.26	15.45	27.74	37.98
1563	5.22	15.41	27.7	37.94
1562	5.18	15.39	27.67	37.9
1561	5.14	15.39	27.66	37.86
1560	5.07	15.36	27.61	37.78
1559	4.93	15.24	27.5	37.65
1558	4.79	15.1	27.36	37.54
1557	4.74	15.01	27.29	37.5
1556	4.72	14.95	27.25	37.47
1555	4.68	14.89	27.2	37.43
1554	4.62	14.82	27.14	37.37
1553	4.57	14.78	27.1	37.33
1552	4.55	14.75	27.07	37.3
1551	4.52	14.72	27.03	37.26
1550	4.48	14.67	26.98	37.21
1549	4.44	14.63	26.94	37.17
1548	4.42	14.61	26.92	37.14
1547	4.4	14.59	26.91	37.12
1546	4.37	14.58	26.89	37.09
1545	4.35	14.57	26.88	37.07
1544	4.34	14.56	26.88	37.06
1543	4.32	14.55	26.88	37.05
1542	4.3	14.52	26.86	37.03
1541	4.28	14.49	26.84	37.02
1540	4.29	14.48	26.82	37.03
1539	4.29	14.47	26.79	37.03
1538	4.27	14.46	26.77	37
1537	4.28	14.46	26.77	37
1536	4.29	14.47	26.79	37
1535	4.3	14.48	26.8	37
1534	4.29	14.47	26.79	36.98
1533	4.29	14.47	26.79	36.96

1532	4.29	14.47	26.78	36.96
1531	4.29	14.46	26.78	36.95
1530	4.29	14.45	26.77	36.93
1529	4.3	14.45	26.78	36.93
1528	4.3	14.47	26.8	36.94
1527	4.29	14.48	26.8	36.94
1526	4.27	14.47	26.78	36.93
1525	4.26	14.47	26.77	36.94
1524	4.28	14.49	26.78	36.95
1523	4.28	14.51	26.78	36.96
1522	4.29	14.51	26.78	36.95
1521	4.31	14.53	26.8	36.97
1520	4.35	14.57	26.84	37
1519	4.37	14.6	26.86	37.02
1518	4.4	14.61	26.86	37.04
1517	4.44	14.64	26.89	37.07
1516	4.51	14.68	26.94	37.13
1515	4.54	14.7	26.97	37.15
1514	4.56	14.72	26.99	37.16
1513	4.59	14.75	27.02	37.18
1512	4.63	14.8	27.06	37.22
1511	4.66	14.84	27.08	37.25
1510	4.67	14.87	27.1	37.29
1509	4.7	14.92	27.14	37.34
1508	4.78	15	27.22	37.42
1507	4.9	15.09	27.3	37.5
1506	4.99	15.15	27.35	37.54
1505	5.06	15.19	27.4	37.59
1504	5.12	15.24	27.45	37.63
1503	5.17	15.28	27.49	37.68
1502	5.22	15.33	27.54	37.72
1501	5.3	15.41	27.61	37.79
1500	5.39	15.51	27.69	37.88
1499	5.48	15.61	27.77	37.95
1498	5.57	15.71	27.85	38.04
1497	5.72	15.88	27.98	38.18
1496	5.91	16.1	28.16	38.36
1495	6.07	16.27	28.29	38.5
1494	6.17	16.39	28.39	38.6
1493	6.25	16.49	28.47	38.68
1492	6.33	16.57	28.54	38.75
1491	6.36	16.59	28.58	38.78
1490	6.37	16.58	28.59	38.78
1489	6.42	16.6	28.64	38.83

1488	6.51	16.68	28.72	38.91
1487	6.58	16.74	28.78	38.97
1486	6.67	16.8	28.85	39.04
1485	6.8	16.91	28.95	39.15
1484	6.95	17.05	29.08	39.29
1483	7.12	17.2	29.22	39.44
1482	7.32	17.39	29.4	39.62
1481	7.58	17.63	29.63	39.85
1480	7.86	17.91	29.89	40.11
1479	8.13	18.18	30.14	40.36
1478	8.43	18.48	30.42	40.64
1477	8.84	18.89	30.81	41.03
1476	9.33	19.39	31.28	41.52
1475	9.87	19.94	31.8	42.05
1474	10.58	20.66	32.48	42.75
1473	11.74	21.81	33.54	43.85
1472	13.18	23.23	34.87	45.2
1471	14.5	24.53	36.09	46.45
1470	15.65	25.66	37.2	47.58
1469	16.8	26.8	38.31	48.7
1468	17.96	27.95	39.44	49.85
1467	19.1	29.09	40.56	50.98
1466	20.24	30.23	41.69	52.12
1465	21.38	31.36	42.81	53.26
1464	22.3	32.28	43.72	54.2
1463	22.87	32.85	44.3	54.79
1462	23.21	33.19	44.66	55.15
1461	23.46	33.45	44.93	55.42
1460	23.68	33.67	45.17	55.64
1459	23.89	33.87	45.38	55.85
1458	24.21	34.18	45.7	56.16
1457	24.64	34.62	46.12	56.61
1456	24.87	34.86	46.36	56.87
1455	24.8	34.81	46.34	56.85
1454	24.62	34.64	46.2	56.69
1453	24.36	34.4	45.99	56.48
1452	24.02	34.08	45.69	56.18
1451	23.61	33.68	45.34	55.82
1450	23.21	33.29	44.98	55.46
1449	22.82	32.9	44.63	55.1
1448	22.4	32.48	44.26	54.72
1447	21.97	32.06	43.88	54.33
1446	21.59	31.69	43.54	53.99
1445	21.25	31.35	43.22	53.67

1444	20.9	30.99	42.88	53.33
1443	20.51	30.6	42.52	52.96
1442	20.12	30.2	42.16	52.59
1441	19.75	29.84	41.82	52.24
1440	19.39	29.48	41.49	51.9
1439	19.02	29.13	41.15	51.55
1438	18.61	28.73	40.76	51.15
1437	18.09	28.22	40.28	50.67
1436	17.48	27.61	39.72	50.11
1435	16.91	27.03	39.19	49.58
1434	16.4	26.53	38.72	49.11
1433	15.9	26.03	38.24	48.62
1432	15.32	25.47	37.7	48.07
1431	14.69	24.85	37.11	47.46
1430	14.04	24.2	36.49	46.84
1429	13.42	23.57	35.9	46.24
1428	12.88	23.02	35.38	45.7
1427	12.43	22.57	34.94	45.26
1426	12.05	22.19	34.57	44.88
1425	11.68	21.81	34.2	44.5
1424	11.31	21.43	33.83	44.14
1423	11.01	21.14	33.54	43.84
1422	10.79	20.94	33.34	43.62
1421	10.62	20.77	33.16	43.44
1420	10.43	20.6	32.98	43.24
1419	10.25	20.43	32.78	43.05
1418	10.08	20.27	32.61	42.88
1417	9.91	20.11	32.44	42.72
1416	9.76	19.94	32.29	42.56
1415	9.63	19.8	32.16	42.43
1414	9.53	19.68	32.05	42.31
1413	9.42	19.56	31.94	42.2
1412	9.31	19.46	31.83	42.09
1411	9.22	19.37	31.74	42
1410	9.15	19.31	31.67	41.92
1409	9.08	19.24	31.6	41.85
1408	9.01	19.17	31.54	41.78
1407	8.96	19.12	31.49	41.73
1406	8.93	19.09	31.45	41.68
1405	8.88	19.04	31.4	41.63
1404	8.83	18.98	31.34	41.57
1403	8.79	18.93	31.29	41.52
1402	8.77	18.9	31.25	41.48
1401	8.74	18.86	31.21	41.44

1400	8.69	18.81	31.15	41.4
1399	8.66	18.77	31.11	41.36
1398	8.64	18.74	31.08	41.34
1397	8.61	18.71	31.06	41.31
1396	8.58	18.69	31.03	41.28
1395	8.59	18.7	31.03	41.28
1394	8.64	18.74	31.06	41.32
1393	8.7	18.8	31.1	41.36
1392	8.79	18.89	31.18	41.44
1391	8.94	19.05	31.32	41.58
1390	9.17	19.29	31.53	41.79
1389	9.49	19.62	31.83	42.1
1388	9.97	20.1	32.27	42.55
1387	10.59	20.72	32.84	43.13
1386	11.24	21.36	33.44	43.75
1385	11.85	21.96	34	44.33
1384	12.47	22.56	34.57	44.91
1383	13.16	23.25	35.22	45.58
1382	13.97	24.04	35.99	46.36
1381	14.86	24.93	36.85	47.23
1380	15.81	25.87	37.79	48.18
1379	16.74	26.79	38.71	49.11
1378	17.49	27.53	39.47	49.87
1377	17.96	27.99	39.96	50.37
1376	18.1	28.13	40.15	50.55
1375	17.91	27.93	40	50.4
1374	17.38	27.42	39.53	49.93
1373	16.67	26.74	38.87	49.27
1372	15.97	26.06	38.21	48.6
1371	15.35	25.45	37.6	48
1370	14.8	24.91	37.07	47.46
1369	14.36	24.48	36.64	47.03
1368	14.05	24.17	36.34	46.73
1367	13.85	23.98	36.16	46.54
1366	13.69	23.83	36.02	46.39
1365	13.49	23.65	35.84	46.21
1364	13.2	23.38	35.57	45.94
1363	12.8	22.99	35.2	45.56
1362	12.36	22.55	34.78	45.13
1361	11.97	22.15	34.4	44.75
1360	11.65	21.83	34.09	44.44
1359	11.4	21.58	33.85	44.19
1358	11.21	21.39	33.66	44
1357	11.06	21.25	33.51	43.86

1356	10.95	21.15	33.41	43.74
1355	10.87	21.08	33.34	43.67
1354	10.82	21.03	33.29	43.61
1353	10.78	21	33.26	43.57
1352	10.75	20.98	33.22	43.54
1351	10.73	20.95	33.19	43.5
1350	10.7	20.92	33.15	43.47
1349	10.66	20.88	33.11	43.43
1348	10.62	20.84	33.07	43.38
1347	10.58	20.8	33.03	43.34
1346	10.54	20.76	33	43.3
1345	10.51	20.73	32.96	43.27
1344	10.49	20.71	32.94	43.25
1343	10.47	20.69	32.92	43.24
1342	10.46	20.69	32.91	43.23
1341	10.46	20.68	32.9	43.23
1340	10.44	20.66	32.88	43.22
1339	10.42	20.63	32.86	43.19
1338	10.38	20.59	32.84	43.15
1337	10.33	20.55	32.8	43.11
1336	10.28	20.5	32.76	43.06
1335	10.24	20.45	32.72	43.02
1334	10.2	20.41	32.69	42.99
1333	10.18	20.38	32.66	42.96
1332	10.15	20.36	32.64	42.94
1331	10.14	20.35	32.63	42.92
1330	10.13	20.34	32.63	42.92
1329	10.12	20.33	32.63	42.92
1328	10.12	20.33	32.62	42.92
1327	10.12	20.33	32.62	42.92
1326	10.12	20.33	32.62	42.93
1325	10.13	20.34	32.63	42.94
1324	10.14	20.35	32.63	42.95
1323	10.15	20.37	32.65	42.96
1322	10.16	20.38	32.66	42.98
1321	10.17	20.4	32.68	42.99
1320	10.19	20.42	32.7	43.02
1319	10.22	20.44	32.73	43.04
1318	10.25	20.47	32.76	43.07
1317	10.28	20.49	32.79	43.09
1316	10.3	20.51	32.81	43.11
1315	10.31	20.52	32.82	43.13
1314	10.32	20.53	32.83	43.14
1313	10.32	20.55	32.84	43.15

1312	10.32	20.55	32.84	43.15
1311	10.33	20.56	32.85	43.16
1310	10.33	20.56	32.85	43.16
1309	10.33	20.55	32.85	43.15
1308	10.32	20.54	32.84	43.14
1307	10.3	20.53	32.82	43.12
1306	10.28	20.51	32.8	43.1
1305	10.25	20.48	32.78	43.08
1304	10.22	20.45	32.74	43.05
1303	10.18	20.41	32.71	43.02
1302	10.14	20.37	32.67	42.99
1301	10.09	20.33	32.63	42.94
1300	10.03	20.28	32.58	42.89
1299	9.97	20.22	32.52	42.83
1298	9.9	20.16	32.46	42.77
1297	9.83	20.09	32.4	42.7
1296	9.76	20.02	32.33	42.64
1295	9.69	19.95	32.27	42.58
1294	9.62	19.88	32.21	42.52
1293	9.55	19.81	32.14	42.46
1292	9.48	19.74	32.08	42.4
1291	9.41	19.69	32.03	42.33
1290	9.35	19.63	31.98	42.28
1289	9.29	19.58	31.93	42.22
1288	9.23	19.52	31.88	42.17
1287	9.17	19.47	31.83	42.13
1286	9.11	19.42	31.79	42.08
1285	9.06	19.37	31.75	42.04
1284	9.02	19.33	31.71	42
1283	8.98	19.3	31.67	41.97
1282	8.95	19.27	31.64	41.94
1281	8.91	19.24	31.61	41.92
1280	8.87	19.21	31.58	41.89
1279	8.84	19.18	31.56	41.87
1278	8.8	19.15	31.53	41.84
1277	8.77	19.12	31.5	41.82
1276	8.73	19.09	31.47	41.79
1275	8.7	19.07	31.45	41.77
1274	8.67	19.04	31.43	41.75
1273	8.63	19.02	31.42	41.73
1272	8.59	18.99	31.41	41.71
1271	8.56	18.97	31.39	41.7
1270	8.54	18.95	31.38	41.69
1269	8.52	18.93	31.38	41.68

1268	8.51	18.92	31.37	41.67
1267	8.51	18.91	31.37	41.67
1266	8.51	18.9	31.38	41.68
1265	8.52	18.89	31.38	41.68
1264	8.52	18.88	31.38	41.69
1263	8.53	18.88	31.39	41.7
1262	8.53	18.88	31.4	41.71
1261	8.54	18.88	31.41	41.72
1260	8.54	18.88	31.42	41.73
1259	8.54	18.87	31.44	41.73
1258	8.54	18.86	31.45	41.74
1257	8.52	18.86	31.45	41.75
1256	8.5	18.84	31.46	41.76
1255	8.48	18.82	31.47	41.77
1254	8.47	18.81	31.47	41.77
1253	8.45	18.8	31.47	41.78
1252	8.44	18.78	31.47	41.78
1251	8.42	18.77	31.47	41.78
1250	8.41	18.76	31.47	41.78
1249	8.39	18.75	31.48	41.78
1248	8.38	18.74	31.48	41.78
1247	8.38	18.73	31.48	41.78
1246	8.38	18.72	31.48	41.79
1245	8.38	18.71	31.49	41.8
1244	8.37	18.7	31.49	41.81
1243	8.37	18.7	31.5	41.83
1242	8.37	18.7	31.51	41.84
1241	8.37	18.7	31.53	41.85
1240	8.36	18.7	31.55	41.87
1239	8.35	18.69	31.56	41.88
1238	8.34	18.68	31.57	41.89
1237	8.33	18.66	31.58	41.9
1236	8.32	18.64	31.59	41.9
1235	8.3	18.63	31.6	41.91
1234	8.29	18.61	31.61	41.91
1233	8.28	18.6	31.62	41.92
1232	8.26	18.58	31.62	41.93
1231	8.25	18.56	31.62	41.93
1230	8.23	18.54	31.63	41.94
1229	8.21	18.52	31.63	41.95
1228	8.19	18.5	31.63	41.95
1227	8.17	18.49	31.64	41.95
1226	8.16	18.48	31.65	41.95
1225	8.15	18.47	31.66	41.95

1224	8.14	18.45	31.66	41.95
1223	8.13	18.44	31.67	41.96
1222	8.12	18.43	31.68	41.97
1221	8.11	18.42	31.69	41.98
1220	8.09	18.41	31.69	41.99
1219	8.08	18.41	31.7	42
1218	8.07	18.4	31.71	42.01
1217	8.06	18.39	31.72	42.01
1216	8.05	18.37	31.72	42.02
1215	8.05	18.37	31.73	42.03
1214	8.04	18.36	31.75	42.05
1213	8.04	18.36	31.76	42.06
1212	8.03	18.36	31.78	42.08
1211	8.04	18.35	31.79	42.09
1210	8.04	18.35	31.8	42.11
1209	8.04	18.35	31.81	42.12
1208	8.03	18.34	31.83	42.13
1207	8.03	18.34	31.84	42.15
1206	8.02	18.34	31.86	42.15
1205	8.01	18.33	31.87	42.16
1204	8	18.32	31.88	42.16
1203	7.99	18.31	31.88	42.16
1202	7.97	18.3	31.88	42.17
1201	7.95	18.29	31.89	42.17
1200	7.93	18.27	31.89	42.17
1199	7.91	18.26	31.89	42.17
1198	7.89	18.24	31.89	42.17
1197	7.88	18.22	31.9	42.18
1196	7.87	18.21	31.9	42.18
1195	7.86	18.2	31.91	42.18
1194	7.86	18.2	31.92	42.18
1193	7.86	18.19	31.92	42.19
1192	7.86	18.18	31.92	42.19
1191	7.85	18.17	31.92	42.19
1190	7.84	18.16	31.93	42.21
1189	7.82	18.16	31.94	42.22
1188	7.81	18.15	31.94	42.23
1187	7.8	18.15	31.95	42.24
1186	7.81	18.14	31.96	42.25
1185	7.81	18.14	31.97	42.26
1184	7.8	18.13	31.98	42.27
1183	7.8	18.12	32	42.28
1182	7.79	18.11	32.01	42.29
1181	7.77	18.1	32.02	42.29

1180	7.75	18.09	32.02	42.3
1179	7.73	18.08	32.02	42.31
1178	7.71	18.06	32.03	42.32
1177	7.7	18.05	32.04	42.33
1176	7.69	18.04	32.05	42.34
1175	7.7	18.04	32.08	42.36
1174	7.71	18.05	32.11	42.39
1173	7.74	18.07	32.15	42.43
1172	7.77	18.1	32.19	42.47
1171	7.8	18.14	32.24	42.53
1170	7.84	18.18	32.29	42.58
1169	7.86	18.21	32.33	42.61
1168	7.88	18.22	32.35	42.64
1167	7.89	18.22	32.37	42.66
1166	7.89	18.21	32.39	42.68
1165	7.88	18.2	32.4	42.69
1164	7.87	18.19	32.42	42.71
1163	7.85	18.17	32.43	42.72
1162	7.84	18.16	32.45	42.73
1161	7.83	18.16	32.47	42.75
1160	7.83	18.16	32.49	42.76
1159	7.83	18.17	32.5	42.78
1158	7.84	18.18	32.53	42.81
1157	7.84	18.18	32.55	42.84
1156	7.84	18.18	32.58	42.86
1155	7.83	18.17	32.61	42.89
1154	7.82	18.16	32.62	42.9
1153	7.79	18.14	32.63	42.91
1152	7.75	18.09	32.62	42.9
1151	7.71	18.04	32.61	42.89
1150	7.66	17.98	32.59	42.87
1149	7.6	17.93	32.58	42.85
1148	7.54	17.87	32.57	42.84
1147	7.48	17.82	32.56	42.82
1146	7.43	17.78	32.56	42.81
1145	7.38	17.73	32.55	42.81
1144	7.34	17.68	32.55	42.8
1143	7.3	17.64	32.54	42.79
1142	7.27	17.6	32.55	42.79
1141	7.23	17.56	32.55	42.79
1140	7.2	17.52	32.56	42.8
1139	7.17	17.49	32.57	42.81
1138	7.14	17.46	32.58	42.82
1137	7.1	17.44	32.6	42.84

1136	7.07	17.41	32.62	42.85
1135	7.03	17.4	32.64	42.88
1134	7.01	17.38	32.66	42.9
1133	6.98	17.36	32.68	42.93
1132	6.97	17.34	32.7	42.96
1131	6.95	17.31	32.73	42.99
1130	6.93	17.29	32.76	43.02
1129	6.91	17.27	32.79	43.05
1128	6.88	17.24	32.82	43.08
1127	6.85	17.22	32.85	43.1
1126	6.83	17.2	32.88	43.13
1125	6.8	17.17	32.89	43.15
1124	6.78	17.14	32.91	43.17
1123	6.76	17.12	32.92	43.18
1122	6.74	17.09	32.94	43.2
1121	6.72	17.07	32.96	43.22
1120	6.7	17.05	32.98	43.23
1119	6.67	17.03	32.99	43.24
1118	6.65	17.03	33	43.25
1117	6.63	17.02	33.01	43.27
1116	6.61	17	33.03	43.29
1115	6.6	16.98	33.05	43.31
1114	6.58	16.96	33.07	43.33
1113	6.57	16.95	33.1	43.35
1112	6.55	16.94	33.12	43.37
1111	6.55	16.93	33.14	43.38
1110	6.55	16.92	33.15	43.4
1109	6.55	16.91	33.16	43.41
1108	6.55	16.9	33.17	43.42
1107	6.55	16.9	33.17	43.42
1106	6.54	16.9	33.17	43.41
1105	6.53	16.9	33.16	43.41
1104	6.52	16.9	33.15	43.4
1103	6.52	16.9	33.14	43.39
1102	6.51	16.89	33.13	43.38
1101	6.5	16.88	33.12	43.36
1100	6.49	16.87	33.12	43.34
1099	6.5	16.86	33.11	43.32
1098	6.51	16.87	33.1	43.31
1097	6.52	16.87	33.09	43.31
1096	6.53	16.88	33.07	43.31
1095	6.55	16.88	33.06	43.29
1094	6.56	16.89	33.05	43.28
1093	6.57	16.91	33.03	43.26

1092	6.58	16.93	33.02	43.24
1091	6.6	16.95	33	43.23
1090	6.61	16.96	32.98	43.21
1089	6.62	16.96	32.96	43.19
1088	6.63	16.95	32.92	43.16
1087	6.63	16.94	32.89	43.13
1086	6.62	16.94	32.86	43.1
1085	6.62	16.94	32.83	43.07
1084	6.62	16.95	32.8	43.03
1083	6.62	16.96	32.77	42.99
1082	6.62	16.98	32.75	42.96
1081	6.64	16.99	32.72	42.93
1080	6.66	17	32.69	42.9
1079	6.68	17.01	32.67	42.87
1078	6.7	17.02	32.64	42.85
1077	6.7	17.03	32.62	42.82
1076	6.7	17.04	32.6	42.8
1075	6.69	17.04	32.56	42.77
1074	6.69	17.05	32.53	42.75
1073	6.69	17.05	32.49	42.71
1072	6.69	17.06	32.45	42.67
1071	6.69	17.06	32.42	42.64
1070	6.7	17.07	32.39	42.6
1069	6.71	17.08	32.37	42.56
1068	6.72	17.09	32.34	42.53
1067	6.73	17.1	32.32	42.51
1066	6.75	17.13	32.3	42.5
1065	6.78	17.15	32.28	42.48
1064	6.8	17.18	32.26	42.47
1063	6.82	17.21	32.25	42.45
1062	6.84	17.22	32.23	42.44
1061	6.84	17.23	32.2	42.41
1060	6.84	17.23	32.17	42.37
1059	6.83	17.22	32.13	42.33
1058	6.82	17.22	32.08	42.28
1057	6.79	17.2	32.03	42.23
1056	6.77	17.18	31.98	42.18
1055	6.73	17.16	31.93	42.12
1054	6.7	17.14	31.88	42.06
1053	6.67	17.12	31.83	42
1052	6.64	17.11	31.78	41.95
1051	6.62	17.1	31.73	41.9
1050	6.6	17.09	31.69	41.85
1049	6.59	17.09	31.65	41.82

1048	6.57	17.1	31.61	41.79
1047	6.57	17.12	31.57	41.77
1046	6.58	17.15	31.56	41.76
1045	6.61	17.19	31.55	41.76
1044	6.64	17.24	31.56	41.77
1043	6.68	17.28	31.57	41.78
1042	6.72	17.34	31.59	41.81
1041	6.77	17.4	31.62	41.85
1040	6.84	17.49	31.66	41.89
1039	6.92	17.59	31.72	41.95
1038	7.02	17.71	31.79	42.02
1037	7.12	17.83	31.87	42.1
1036	7.24	17.96	31.95	42.19
1035	7.37	18.08	32.04	42.29
1034	7.48	18.2	32.12	42.37
1033	7.57	18.3	32.17	42.43
1032	7.62	18.37	32.19	42.46
1031	7.62	18.41	32.18	42.45
1030	7.6	18.41	32.15	42.41
1029	7.55	18.39	32.09	42.35
1028	7.48	18.34	32.02	42.28
1027	7.41	18.28	31.94	42.2
1026	7.33	18.23	31.86	42.12
1025	7.26	18.18	31.78	42.04
1024	7.19	18.14	31.72	41.97
1023	7.14	18.11	31.66	41.9
1022	7.1	18.08	31.6	41.84
1021	7.06	18.05	31.54	41.78
1020	7.03	18.03	31.49	41.72
1019	6.99	17.99	31.44	41.67
1018	6.96	17.96	31.39	41.62
1017	6.94	17.93	31.34	41.57
1016	6.91	17.89	31.28	41.51
1015	6.89	17.86	31.22	41.46
1014	6.87	17.83	31.17	41.4
1013	6.84	17.8	31.11	41.35
1012	6.81	17.77	31.05	41.29
1011	6.78	17.74	31	41.23
1010	6.75	17.7	30.95	41.18
1009	6.74	17.67	30.9	41.13
1008	6.72	17.64	30.86	41.09
1007	6.72	17.62	30.82	41.05
1006	6.72	17.61	30.79	41.02
1005	6.73	17.6	30.77	40.99

1004	6.73	17.61	30.75	40.96
1003	6.75	17.62	30.73	40.94
1002	6.76	17.63	30.71	40.93
1001	6.78	17.64	30.69	40.92
1000	6.8	17.66	30.68	40.92
999	6.82	17.67	30.68	40.91
998	6.84	17.68	30.67	40.9
997	6.86	17.69	30.67	40.89
996	6.87	17.69	30.66	40.88
995	6.89	17.68	30.65	40.86
994	6.89	17.67	30.62	40.84
993	6.88	17.63	30.57	40.79
992	6.85	17.58	30.51	40.73
991	6.8	17.52	30.44	40.66
990	6.74	17.45	30.37	40.59
989	6.68	17.38	30.3	40.53
988	6.63	17.32	30.24	40.47
987	6.6	17.27	30.2	40.42
986	6.6	17.24	30.18	40.38
985	6.61	17.23	30.16	40.36
984	6.63	17.23	30.15	40.35
983	6.66	17.26	30.16	40.36
982	6.71	17.3	30.19	40.39
981	6.77	17.35	30.23	40.43
980	6.86	17.43	30.29	40.49
979	6.96	17.53	30.36	40.57
978	7.07	17.65	30.44	40.65
977	7.2	17.79	30.54	40.75
976	7.34	17.96	30.66	40.87
975	7.5	18.15	30.8	41
974	7.69	18.37	30.96	41.17
973	7.89	18.62	31.14	41.36
972	8.12	18.89	31.35	41.57
971	8.37	19.18	31.57	41.79
970	8.62	19.48	31.79	42
969	8.85	19.75	31.99	42.21
968	9.05	19.98	32.17	42.38
967	9.19	20.13	32.3	42.5
966	9.26	20.2	32.36	42.56
965	9.24	20.16	32.33	42.53
964	9.14	20.01	32.23	42.43
963	8.96	19.78	32.06	42.27
962	8.72	19.5	31.85	42.06
961	8.46	19.19	31.62	41.81

960	8.19	18.87	31.39	41.57
959	7.94	18.57	31.17	41.34
958	7.72	18.3	30.98	41.13
957	7.53	18.07	30.81	40.96
956	7.37	17.87	30.67	40.81
955	7.23	17.71	30.55	40.68
954	7.11	17.58	30.44	40.56
953	7.02	17.47	30.35	40.47
952	6.94	17.37	30.26	40.39
951	6.88	17.27	30.18	40.32
950	6.83	17.19	30.12	40.24
949	6.77	17.11	30.06	40.18
948	6.71	17.03	30.02	40.13
947	6.65	16.97	29.98	40.09
946	6.61	16.92	29.94	40.05
945	6.57	16.88	29.9	40.01
944	6.53	16.84	29.85	39.96
943	6.49	16.81	29.81	39.92
942	6.46	16.77	29.77	39.88
941	6.43	16.73	29.73	39.85
940	6.42	16.69	29.69	39.82
939	6.4	16.66	29.66	39.79
938	6.38	16.63	29.64	39.76
937	6.36	16.6	29.61	39.73
936	6.33	16.58	29.57	39.7
935	6.3	16.55	29.54	39.67
934	6.27	16.52	29.52	39.65
933	6.24	16.49	29.5	39.62
932	6.2	16.46	29.47	39.6
931	6.16	16.43	29.44	39.56
930	6.12	16.4	29.4	39.52
929	6.08	16.37	29.37	39.48
928	6.05	16.33	29.34	39.45
927	6.02	16.3	29.31	39.42
926	6.01	16.29	29.29	39.4
925	6.01	16.28	29.28	39.4
924	6.02	16.29	29.29	39.41
923	6.04	16.33	29.31	39.43
922	6.07	16.37	29.34	39.45
921	6.12	16.44	29.37	39.48
920	6.19	16.51	29.4	39.53
919	6.26	16.6	29.43	39.58
918	6.34	16.7	29.49	39.65
917	6.42	16.82	29.56	39.73

916	6.52	16.95	29.65	39.8
915	6.65	17.09	29.76	39.9
914	6.79	17.25	29.88	40
913	6.93	17.4	29.99	40.11
912	7.05	17.54	30.09	40.2
911	7.14	17.64	30.17	40.27
910	7.19	17.69	30.21	40.3
909	7.18	17.68	30.21	40.31
908	7.13	17.62	30.17	40.28
907	7.06	17.53	30.11	40.24
906	6.98	17.42	30.04	40.18
905	6.91	17.32	29.97	40.12
904	6.85	17.24	29.91	40.07
903	6.8	17.19	29.88	40.05
902	6.78	17.17	29.88	40.06
901	6.78	17.17	29.9	40.08
900	6.82	17.19	29.94	40.12
899	6.89	17.24	30	40.18
898	6.98	17.3	30.08	40.25
897	7.07	17.39	30.17	40.34
896	7.16	17.49	30.27	40.45
895	7.26	17.6	30.38	40.55
894	7.38	17.69	30.48	40.66
893	7.5	17.79	30.59	40.77
892	7.62	17.88	30.7	40.87
891	7.73	17.98	30.81	40.97
890	7.83	18.08	30.91	41.08
889	7.92	18.18	31	41.18
888	8	18.28	31.08	41.28
887	8.08	18.38	31.16	41.38
886	8.18	18.47	31.24	41.46
885	8.29	18.56	31.33	41.54
884	8.4	18.66	31.43	41.63
883	8.5	18.76	31.52	41.72
882	8.61	18.86	31.62	41.82
881	8.71	18.96	31.73	41.93
880	8.82	19.06	31.84	42.04
879	8.92	19.16	31.96	42.15
878	9.03	19.28	32.07	42.26
877	9.13	19.39	32.17	42.36
876	9.23	19.49	32.26	42.45
875	9.33	19.59	32.36	42.55
874	9.43	19.67	32.45	42.65
873	9.52	19.75	32.54	42.75

872	9.6	19.82	32.62	42.83
871	9.68	19.89	32.7	42.9
870	9.76	19.95	32.78	42.96
869	9.83	20	32.85	43.02
868	9.89	20.05	32.91	43.08
867	9.94	20.1	32.96	43.13
866	9.99	20.13	33	43.18
865	10.02	20.15	33.02	43.21
864	10.04	20.16	33.04	43.23
863	10.05	20.17	33.05	43.24
862	10.04	20.16	33.05	43.23
861	10.02	20.13	33.04	43.22
860	9.98	20.08	33.02	43.19
859	9.93	20.03	32.98	43.15
858	9.88	19.98	32.94	43.11
857	9.84	19.94	32.9	43.07
856	9.8	19.9	32.86	43.02
855	9.77	19.85	32.84	42.99
854	9.72	19.8	32.82	42.96
853	9.66	19.75	32.8	42.94
852	9.59	19.7	32.79	42.91
851	9.52	19.65	32.77	42.89
850	9.47	19.6	32.76	42.87
849	9.42	19.56	32.74	42.85
848	9.38	19.51	32.72	42.83
847	9.34	19.47	32.7	42.82
846	9.29	19.43	32.67	42.8
845	9.24	19.38	32.65	42.78
844	9.2	19.33	32.62	42.76
843	9.17	19.28	32.61	42.74
842	9.14	19.24	32.6	42.72
841	9.11	19.2	32.59	42.7
840	9.06	19.17	32.57	42.68
839	9.03	19.16	32.55	42.67
838	9.02	19.16	32.54	42.68
837	9.03	19.19	32.55	42.71
836	9.07	19.24	32.58	42.74
835	9.12	19.3	32.63	42.79
834	9.18	19.37	32.7	42.86
833	9.26	19.45	32.77	42.95
832	9.36	19.55	32.85	43.04
831	9.46	19.65	32.94	43.13
830	9.58	19.76	33.05	43.23
829	9.71	19.87	33.16	43.34

828	9.85	19.98	33.29	43.45
827	9.99	20.09	33.41	43.56
826	10.14	20.22	33.54	43.69
825	10.27	20.36	33.67	43.83
824	10.4	20.51	33.81	43.98
823	10.53	20.67	33.94	44.12
822	10.67	20.82	34.08	44.27
821	10.83	20.98	34.22	44.41
820	10.99	21.14	34.36	44.55
819	11.15	21.3	34.5	44.68
818	11.3	21.46	34.64	44.81
817	11.45	21.62	34.78	44.93
816	11.59	21.76	34.9	45.05
815	11.72	21.87	35	45.16
814	11.81	21.96	35.08	45.26
813	11.88	22.01	35.13	45.33
812	11.93	22.03	35.17	45.38
811	11.95	22.04	35.19	45.42
810	11.97	22.03	35.21	45.43
809	11.97	22.01	35.21	45.42
808	11.94	21.97	35.2	45.4
807	11.89	21.92	35.16	45.35
806	11.82	21.85	35.09	45.29
805	11.73	21.76	35	45.19
804	11.62	21.65	34.89	45.07
803	11.48	21.52	34.76	44.94
802	11.33	21.38	34.63	44.8
801	11.18	21.23	34.5	44.66
800	11.02	21.08	34.36	44.51
799	10.87	20.94	34.22	44.37
798	10.73	20.8	34.08	44.24
797	10.58	20.67	33.95	44.11
796	10.42	20.53	33.82	43.98
795	10.28	20.4	33.7	43.86
794	10.14	20.28	33.59	43.75
793	10.02	20.16	33.48	43.64
792	9.91	20.06	33.37	43.52
791	9.82	19.97	33.26	43.4
790	9.74	19.88	33.17	43.3
789	9.68	19.81	33.08	43.22
788	9.63	19.75	33.02	43.15
787	9.6	19.72	32.97	43.1
786	9.58	19.72	32.94	43.06
785	9.58	19.75	32.92	43.05

784	9.58	19.78	32.93	43.04
783	9.6	19.81	32.95	43.04
782	9.62	19.84	32.98	43.06
781	9.65	19.87	32.99	43.09
780	9.68	19.89	32.99	43.13
779	9.71	19.93	33	43.15
778	9.76	19.97	33.02	43.18
777	9.81	20.02	33.05	43.21
776	9.87	20.07	33.1	43.25
775	9.94	20.15	33.16	43.3
774	10.02	20.24	33.24	43.38
773	10.12	20.34	33.33	43.47
772	10.24	20.46	33.42	43.57
771	10.37	20.59	33.52	43.67
770	10.5	20.75	33.64	43.78
769	10.64	20.91	33.76	43.9
768	10.78	21.08	33.88	44.03
767	10.93	21.24	34.01	44.17
766	11.08	21.39	34.16	44.31
765	11.23	21.54	34.3	44.45
764	11.37	21.68	34.45	44.59
763	11.52	21.81	34.59	44.73
762	11.67	21.95	34.73	44.88
761	11.82	22.08	34.87	45.02
760	11.96	22.21	34.99	45.15
759	12.07	22.34	35.11	45.26
758	12.17	22.46	35.21	45.36
757	12.24	22.57	35.29	45.45
756	12.31	22.66	35.36	45.54
755	12.38	22.75	35.44	45.63
754	12.46	22.83	35.52	45.72
753	12.55	22.91	35.61	45.8
752	12.64	22.99	35.71	45.87
751	12.73	23.07	35.8	45.94
750	12.82	23.16	35.87	46.01
749	12.91	23.23	35.95	46.09
748	12.99	23.29	36.03	46.17
747	13.06	23.34	36.1	46.24
746	13.11	23.38	36.15	46.29
745	13.11	23.38	36.16	46.3
744	13.07	23.34	36.12	46.27
743	12.99	23.25	36.05	46.19
742	12.86	23.11	35.94	46.07
741	12.7	22.95	35.81	45.92

740	12.5	22.78	35.65	45.74
739	12.29	22.6	35.46	45.56
738	12.09	22.43	35.28	45.39
737	11.93	22.28	35.11	45.24
736	11.81	22.16	34.98	45.13
735	11.74	22.09	34.91	45.06
734	11.72	22.05	34.89	45.03
733	11.75	22.07	34.93	45.07
732	11.81	22.13	35.02	45.15
731	11.89	22.22	35.13	45.25
730	11.98	22.32	35.24	45.34
729	12.08	22.42	35.33	45.42
728	12.17	22.49	35.4	45.47
727	12.26	22.56	35.46	45.52
726	12.35	22.64	35.51	45.58
725	12.41	22.72	35.57	45.65
724	12.45	22.78	35.63	45.72
723	12.47	22.82	35.67	45.78
722	12.44	22.79	35.67	45.8
721	12.33	22.68	35.6	45.73
720	12.1	22.47	35.43	45.55
719	11.77	22.16	35.15	45.25
718	11.36	21.76	34.76	44.85
717	10.92	21.31	34.33	44.42
716	10.48	20.87	33.89	44
715	10.09	20.47	33.5	43.62
714	9.74	20.12	33.17	43.28
713	9.45	19.84	32.88	42.99
712	9.2	19.59	32.63	42.73
711	9	19.39	32.43	42.53
710	8.85	19.23	32.29	42.38
709	8.76	19.13	32.19	42.27
708	8.74	19.12	32.15	42.22
707	8.8	19.21	32.17	42.24
706	8.95	19.41	32.28	42.36
705	9.19	19.73	32.48	42.57
704	9.54	20.16	32.76	42.86
703	9.97	20.66	33.12	43.21
702	10.46	21.22	33.53	43.6
701	10.93	21.75	33.94	43.99
700	11.3	22.18	34.25	44.29
699	11.48	22.39	34.4	44.45
698	11.38	22.3	34.31	44.39
697	10.97	21.87	33.97	44.07

696	10.33	21.17	33.44	43.55
695	9.6	20.34	32.84	42.94
694	8.9	19.53	32.26	42.35
693	8.34	18.85	31.79	41.86
692	7.93	18.35	31.44	41.49
691	7.65	18.01	31.19	41.23
690	7.46	17.79	31.01	41.04
689	7.31	17.64	30.86	40.9
688	7.18	17.52	30.75	40.78
687	7.09	17.42	30.67	40.68
686	7.01	17.33	30.61	40.61
685	6.96	17.26	30.55	40.54
684	6.91	17.2	30.5	40.49
683	6.87	17.16	30.45	40.44
682	6.82	17.12	30.41	40.41
681	6.76	17.08	30.36	40.38
680	6.7	17.05	30.33	40.35
679	6.64	17.01	30.29	40.33
678	6.6	16.97	30.24	40.31
677	6.58	16.93	30.2	40.29
676	6.56	16.88	30.17	40.25
675	6.54	16.82	30.14	40.2
674	6.52	16.78	30.12	40.14
673	6.49	16.73	30.09	40.09
672	6.46	16.7	30.06	40.05
671	6.42	16.68	30.03	40.01
670	6.37	16.67	29.99	39.99
669	6.3	16.63	29.92	39.97
668	6.24	16.57	29.85	39.96
667	6.23	16.52	29.84	39.95
666	6.25	16.49	29.87	39.92
665	6.25	16.48	29.88	39.9
664	6.24	16.47	29.87	39.88
663	6.22	16.46	29.85	39.87
662	6.2	16.44	29.83	39.87
661	6.17	16.41	29.82	39.86
660	6.16	16.38	29.8	39.86
659	6.15	16.37	29.79	39.84
658	6.15	16.36	29.77	39.83
657	6.15	16.35	29.74	39.8
656	6.14	16.34	29.71	39.77
655	6.12	16.31	29.68	39.75
654	6.11	16.29	29.68	39.74
653	6.1	16.27	29.69	39.74

652	6.08	16.26	29.71	39.74
651	6.07	16.26	29.73	39.75
650	6.08	16.27	29.74	39.74
649	6.08	16.29	29.74	39.74
648	6.08	16.3	29.74	39.73
647	6.07	16.3	29.74	39.74
646	6.08	16.29	29.74	39.76
645	6.09	16.27	29.74	39.78
644	6.11	16.26	29.75	39.8
643	6.12	16.27	29.76	39.81
642	6.14	16.29	29.78	39.82
641	6.15	16.29	29.8	39.82
640	6.16	16.29	29.82	39.83
639	6.16	16.27	29.85	39.84
638	6.15	16.27	29.86	39.85
637	6.14	16.28	29.86	39.85
636	6.12	16.29	29.84	39.85
635	6.11	16.3	29.82	39.84
634	6.11	16.3	29.81	39.83
633	6.1	16.3	29.81	39.82
632	6.1	16.3	29.8	39.82
631	6.1	16.31	29.79	39.82
630	6.11	16.32	29.79	39.82
629	6.12	16.33	29.8	39.83
628	6.13	16.33	29.81	39.84
627	6.13	16.33	29.82	39.85
626	6.13	16.33	29.83	39.86
625	6.13	16.34	29.83	39.87
624	6.12	16.36	29.83	39.87
623	6.13	16.37	29.83	39.87
622	6.16	16.38	29.85	39.87
621	6.19	16.38	29.86	39.88
620	6.21	16.38	29.87	39.88
619	6.2	16.38	29.88	39.88
618	6.18	16.39	29.89	39.88
617	6.15	16.4	29.89	39.91
616	6.13	16.41	29.89	39.93
615	6.13	16.41	29.88	39.93
614	6.14	16.41	29.87	39.92
613	6.15	16.4	29.87	39.91
612	6.17	16.4	29.87	39.91
611	6.17	16.41	29.88	39.91
610	6.18	16.42	29.91	39.93
609	6.2	16.42	29.94	39.95

608	6.21	16.41	29.95	39.98
607	6.21	16.41	29.95	40
606	6.22	16.41	29.94	40.02
605	6.24	16.42	29.94	40.03
604	6.24	16.44	29.96	40.04
603	6.25	16.48	29.98	40.06
602	6.25	16.52	30.01	40.08
601	6.26	16.54	30.04	40.11
600	6.27	16.54	30.08	40.13
599	6.3	16.53	30.11	40.15
598	6.32	16.53	30.14	40.18
597	6.34	16.55	30.15	40.2
596	6.35	16.59	30.16	40.21
595	6.36	16.62	30.17	40.21
594	6.37	16.64	30.19	40.22
593	6.39	16.64	30.2	40.23
592	6.41	16.64	30.21	40.23
591	6.42	16.65	30.21	40.23
590	6.44	16.67	30.23	40.24
589	6.47	16.69	30.25	40.26
588	6.49	16.71	30.27	40.28
587	6.5	16.7	30.29	40.3
586	6.5	16.7	30.32	40.31
585	6.49	16.7	30.35	40.32
584	6.48	16.71	30.39	40.34
583	6.48	16.73	30.42	40.36
582	6.49	16.75	30.44	40.4
581	6.51	16.79	30.46	40.44
580	6.53	16.83	30.49	40.46
579	6.56	16.86	30.5	40.49
578	6.6	16.88	30.52	40.52
577	6.63	16.88	30.53	40.55
576	6.65	16.87	30.54	40.55
575	6.64	16.88	30.54	40.53
574	6.62	16.9	30.52	40.51
573	6.6	16.91	30.49	40.5
572	6.6	16.91	30.48	40.51
571	6.61	16.89	30.47	40.53
570	6.61	16.86	30.49	40.54
569	6.61	16.84	30.52	40.55
568	6.6	16.87	30.55	40.56
567	6.62	16.91	30.58	40.57
566	6.64	16.94	30.59	40.59
565	6.67	16.94	30.59	40.6

564	6.69	16.94	30.59	40.61
563	6.7	16.96	30.59	40.62
562	6.71	16.98	30.6	40.62
561	6.72	16.99	30.61	40.63
560	6.73	16.99	30.59	40.63
559	6.76	16.98	30.57	40.64
558	6.78	16.95	30.55	40.63
557	6.79	16.91	30.55	40.62
556	6.78	16.86	30.58	40.6
555	6.76	16.84	30.61	40.58
554	6.75	16.83	30.63	40.58
553	6.75	16.84	30.62	40.58
552	6.76	16.85	30.6	40.59
551	6.76	16.86	30.58	40.58
550	6.75	16.87	30.57	40.58
549	6.73	16.9	30.57	40.58
548	6.72	16.94	30.58	40.59
547	6.73	16.99	30.61	40.61
546	6.76	17.03	30.63	40.64
545	6.8	17.04	30.65	40.66
544	6.82	17.03	30.65	40.68
543	6.84	17.03	30.65	40.7
542	6.85	17.04	30.66	40.73
541	6.87	17.08	30.69	40.75
540	6.89	17.11	30.71	40.75
539	6.91	17.12	30.7	40.72
538	6.89	17.1	30.68	40.69
537	6.83	17.05	30.64	40.68
536	6.77	17	30.61	40.67
535	6.72	16.95	30.58	40.66
534	6.68	16.91	30.55	40.63
533	6.63	16.86	30.52	40.59
532	6.56	16.79	30.48	40.54
531	6.51	16.72	30.43	40.5
530	6.47	16.66	30.39	40.45
529	6.45	16.62	30.36	40.43
528	6.44	16.58	30.35	40.4
527	6.44	16.55	30.37	40.37
526	6.44	16.51	30.38	40.33
525	6.42	16.47	30.36	40.28
524	6.39	16.41	30.34	40.25
523	6.37	16.38	30.33	40.23
522	6.34	16.36	30.31	40.23
521	6.31	16.36	30.27	40.27

520	6.25	16.36	30.22	40.32
519	6.18	16.35	30.17	40.36
518	6.15	16.33	30.16	40.39
517	6.17	16.3	30.18	40.37
516	6.2	16.26	30.21	40.34
515	6.22	16.24	30.25	40.31
514	6.22	16.23	30.29	40.3
513	6.2	16.23	30.34	40.32
512	6.17	16.21	30.38	40.36
511	6.16	16.2	30.44	40.37
510	6.15	16.19	30.5	40.37
509	6.15	16.19	30.54	40.37
508	6.12	16.19	30.54	40.39
507	6.09	16.18	30.54	40.44
506	6.08	16.16	30.55	40.53
505	6.09	16.14	30.58	40.62
504	6.1	16.12	30.63	40.71
503	6.11	16.12	30.67	40.76
502	6.11	16.12	30.73	40.79
501	6.09	16.12	30.79	40.83
500	6.08	16.11	30.85	40.9
